# Population attributable fractions for risk factors for spontaneous preterm births in 81 low- and middle-income countries: A systematic analysis

**DOI:** 10.7189/jogh.12.04013

**Published:** 2022-03-26

**Authors:** Emily Bryce, Sabi Gurung, Hannah Tong, Joanne Katz, Anne CC Lee, Robert E Black, Neff Walker

**Affiliations:** 1 Department of International Health, Bloomberg School of Public Health, Johns Hopkins University, Baltimore, Maryland, USA.; 2 Department of Pediatric Newborn Medicine, Brigham and Women’s Hospital, Boston, Massachusetts, USA

## Abstract

**Background:**

Complications associated with preterm birth (PTB) are the largest contributor to under-five mortality globally. Success in reaching the Sustainable Development Goal target requires identifying potentially modifiable risk factors for PTB, estimating the relative importance of these risk factors, and identifying/implementing effective prevention strategies to address them.

**Methods:**

We conducted a literature review to define risk relationships and estimate prevalence for established risk factors for spontaneous PTB (sPTB). We then estimated population attributable fractions (PAF) for the sPTB risk factors identified in the review as statistically significant for the 81 low- and middle-income (LMIC) countries included in the Countdown 2030 initiative. We summed country-level findings to produce PAFs for each risk factor and regional estimates for sub-Saharan Africa and South Asia.

**Results:**

Forty-four potential sPTB risk factors were identified. and the final analysis included twenty-four risk factors with evidence of significant associations with sPTB. A second model with three additional risk factors with borderline insignificant associations was also run. Taken together, the twenty-four risk factors had a total PAF of 73% for all 81 countries and 77% and 72% of sPTB in sub-Saharan Africa and South Asia, respectively. For all countries, maternal undernutrition had the highest PAF (17.5%), followed by maternal infections (16.6%), environmental exposures (16%) and pregnancy history (8.7%).

**Conclusions:**

While multiple risk factors contribute to sPTB, no single risk factor addresses a predominant fraction, and 27% of spontaneous preterm births are not associated with risk factors that we identified. Despite the significant role of preterm birth in child survival, there are major data gaps in LMIC settings. Furthermore, there is a paucity of evidence for effective interventions to prevent preterm birth. Preventing sPTB requires understanding underlying mechanisms leading to sPTB in different populations, and the identification/implementation of effective interventions.

The third Sustainable Development Goal (SDG) has a target of reducing the under-five mortality rate to 25 deaths in children under the age of five per 1000 live births by 2030 [1]. Complications of preterm birth (PTB), are the leading cause of death among children under the age of five years [2]. Over 80 percent (81.1%) of PTB deaths occur in Asia and sub-Saharan Africa [3]. Preterm infants that survive may experience lifelong disability such as hearing, vision or cognitive impairment, as well as higher rates of chronic disease and mortality than full-term infants [4,5]. A better understanding of the risks for and causes of preterm births is essential if we are to prevent preterm birth and reduce preterm-related deaths and morbidities in low- and middle-income countries (LMIC).

Preterm birth is classified as provider initiated or spontaneous [6]. Spontaneous preterm birth (sPTB) is defined as the start of labor prior to 37 weeks of gestation with either intact or pre-labor ruptured membranes [7]. In LMICs, over 75% of PTB are spontaneous [8]. Spontaneous PTB usually results from a combination of risk factors, rather than a single risk factor [6,9]. PTB with a single risk factor are more common for provider-initiated preterm births [6].

Available evidence on the population-attributable risk of various risk factors for sPTB is incomplete for several reasons. First, much of the available evidence on sPTB risk relationships has been generated through disease- or condition-specific studies. The examination of a single risk factor often overestimates its attributable fraction because the analysis may not account for the contribution of other correlated sPTB risk factors. On the other hand, disease-specific studies may be rigorous trials of antenatal interventions, providing causal data on the relationship between the presence of a single condition by the reduction in sPTB risk. The outcome variable reported is more commonly low birthweight than PTB, given the challenges of measuring gestational age in LMIC [10]. Furthermore, for interventions that have now become standard of care, trials to re-examine the intervention impact on sPTB would be unethical due to lack of equipoise.

Despite these limitations in the available data, there is an urgent need to understand the relative public health importance of various risk factors for spontaneous preterm births. The purpose of this study was to reexamine existing data to estimate the relative contribution of previously identified risk factors for sPTB, as a basis for developing and/or implementing interventions to reduce the incidence of preterm births. We use the available evidence to identify risk factors for sPTB and their prevalence, to quantify the risk relationships and to assess the quality of the evidence supporting them. We use population-attributable fractions (PAFs) to estimate the contribution of individual risk factors to sPTB in LMIC as a guide to identification of possible interventions.

## METHODS

### Countries

We ran the analyses for the 81 Countdown to 2030 countries [11]. Together, these LMIC countries accounted for over 90% of child (under-five) mortality and 95% of maternal mortality in 2015, [12] and roughly 90% percent of the estimated global preterm births in 2014 (the most recent estimate available). Additionally, we present results for two regions: sub-Saharan Africa (41 countries) and South Asia (Bangladesh, Bhutan, India, Nepal, Pakistan).

### Estimation of PAF procedures

Overall, we had four steps in developing estimates of PAF’s for risk factors for preterm births. First, we conducted a literature review to identify possible risk factors for PTB and to determine its relationship with sPTB. Next, we reviewed the literature to identify studies to estimate the effects of each identified risk factor and included measures of study quality to determine which risk factors should be included in the PAF model. Third, for each factor found to be related to risk of sPTB we also searched for estimates of prevalence of these factors in low- and middle-income countries. These estimates were also judged for quality and adjusted for current levels of treatment or interventions that would eliminate or reduce risk. Finally, using standard techniques we computed country-specific estimates of sPAFs for 81 low- and middle-income countries.

### Identifying potential risk factors for preterm birth

This analysis focuses specifically on risk factors for spontaneous preterm birth, where delivery occurs prior to 37-weeks gestational age and labor is not induced. We chose to focus solely on spontaneous preterm births because in the 81 Countdown countries that are the focus of our analysis, over 75% of preterm births are spontaneous [8]. The initial list of risk factors for spontaneous preterm birth were identified by reviewing two syntheses, [4,13], which were developed by experts in the field and confirmed by comparing risk factors identified in the published literature [6,14-17]. Additionally, findings were presented to experts in the field during analysis for an informal expert-checking process. To ensure that the risk factors in our model were for spontaneous preterm birth, the Preterm Prediction Study, which analyzed over 40 risk factors for iatrogenic (provider-initiated) preterm birth, was referenced. In this analysis pre-eclampsia, fetal distress, IUGR, placental abruption, and fetal death were the most frequent (%) causes of iatrogenic preterm birth. These authors compared the risk factors for iatrogenic preterm birth to their analysis of risk factors for spontaneous preterm birth and found little overlap [18]. In our analysis we have done our best to account for the few risk factors that do overlap by including a risk estimate specifically for spontaneous preterm birth. If the primary risk estimate is from a meta-analysis (as these were given preference over single studies in our quality assessment) that does not make this distinction, individual studies that do disaggregate by type of preterm birth were identified to ensure that the risk relationship was not overturned when the distinction is made. This process yielded 44 possible risk factors for sPTB. We excluded risk factors for three reasons: (1) adequate evidence of the *absence* of a risk relationship with sPTB; (2) insufficient evidence of a relationship between the risk factor and sPTB; or (3) the risk factor is not independent of other sPTB risk factors, ie, the risk factor is either an intermediate outcome between another risk factor and sPTB, or a composite of other risk factors already considered. The search strategy and evidence supporting decisions on inclusion of a risk factor in the modeling process are described in detail in Appendices S2 and S4 in the **Online Supplementary Document**.

*Quality of risk estimate:* We conducted a literature search on each of the 44 potential risk factors for sPTB. Interventions implemented during pregnancy were included; those delivered pre-conception were not considered. Priority was given to meta-analyses. We used PRISMA and GRADE checklists as references and created two revised checklists (Table S4a and Table S4b in the **Online Supplementary Document**) to score studies based on factors such as design; study site; definition of predictor and outcome variables; adjustment of confounders; heterogeneity; magnitude of the association. There were two possible quality scores produced for each risk factor: one quality score of the sources for observational risk and, if applicable, one for the intervention. For observational risk estimates, the maximum possible score was 14. The maximum possible score for intervention impact estimates was 17. For observational risk sources, a score greater than 10 was deemed “high” quality, those with a score of 8 to 10 were “medium” quality and those with a score less than 8 were “low” quality. The corresponding cut-offs for high, medium and low quality for intervention evidence were greater than 13, 10 to 13 and below 10, respectively. For those risk factors that do not have an intervention delivered during the antenatal period, there was only one quality score for the observational risk estimate. We graded the overall quality of evidence for each risk factor using a six-level categorization (high, medium-high, medium, medium-low, low, and very low) that considered both observational and intervention evidence quality. The rules for assigning the overall quality are presented in Table S4c in the **Online Supplementary Document**. The risk estimates used in the model were from observational data for all risk factors except for low zinc and low calcium intake. In these two cases, the inverse of the intervention impact is used to represent the risk estimate and the quality score is strictly based on the intervention estimate.

### Risk estimates included in the analysis models

A total of 24 risk factors are included in the final model, which is comprised of risk factors with evidence with adequate quality and a statistically significant relationship with the outcome. We also developed an extended model with 27 risk factors including three additional risk factors that have medium/high quality evidence but a borderline insignificant effect. The three risk factors included in the extended model are low calcium intake, low zinc intake and Group B Streptococcus infection.

### Estimates of prevalence of risk.

The estimates of risk factor prevalence from recent national household surveys (eg, Demographic and Health Survey (DHS) or Multiple Indicator Cluster Survey (MICS)) were used when available. For countries without these nationally representative surveys, we estimated country-specific prevalence as the regional average of those with national data, using WHO regions [19]. For risk factors not measured in most household surveys, the next preferred option was those produced by the Institute for Health Metrics and Evaluation (IHME), or UNICEF [20,21]. When these data were not available, meta-analyses that produced regional prevalence estimates for the risk factors were used. Only regional data, or in the case of short cervical length a global average, were available for some risk factors. The weighted prevalence of the risk factors by World Health Organization region can be found Table S3 in the **Online Supplementary Document**).

### Quality of prevalence estimates.

We defined three categories for the quality of prevalence estimates, which were reflective of the source of data. A “high” quality source is country-specific prevalence data collected via nationally representative surveys (eg, DHS or MICS). A “medium” quality source is country-specific prevalence data estimated from a model (eg, estimates generated by IHME). A “low” quality source is regional data generated by any method (meta-analysis of primary sources, summarized survey data, etc.) and applied to the country level or if the prevalence estimate is not specific to pregnant women or women of reproductive age. There are five risk factors where there are both country-level and regional-level prevalence data (DHS or MICS data where regional estimates are imputed for those countries in a region without country-specific data), depending on the country. These received a “medium” quality grade as they combine high- and low-quality prevalence estimates.

### Treatment adjustment of prevalence estimates.

For certain risk factors, there are interventions delivered during pregnancy that significantly reduce the associated risk of preterm birth (**Table 1**). For these factors, the prevalence was adjusted to account for this treatment effect using the following equation: *Prevalenceadjusted = (Prevalence unadjusted × Coverage of treatment × Efficacy of treatment).* In cases where coverage data were not available for a treatment, we estimated coverage using an adjusted “receipt of ANC4+” estimate, calculated by multiplying the ANC4+ coverage estimate by an estimate of ANC quality (specifically, the percentage of facilities prepared to deliver syphilis test and treatment). Details on data sources for and adjustments to prevalence estimates for each risk factor are available in Appendices S2 and S3 of the **Online Supplemental Document**.

**Table 1:** Estimated relative risk for factors associated with preterm birth factors in final & extended model*

Risk factor	Relative risk (95% CI)	RR source	Risk quality*^a^	Intervention	Intervention quality	Overall quality	Agreement on effects	Prevalence	Prevalence quality
**Pregnancy history:**									
Short birth interval	1.49 (1.17-1.89) [22]	Obs	High	RF not amenable to intervention during pregnancy	NA	Medium	NA	Country specific	High
Young maternal age (<18 years) & primiparity	1.46 (1.36-1.58) [23]	Obs	High	RF not amenable to intervention during pregnancy	NA	Medium	NA	Country specific	High
Maternal age 18-35 years & parity >3	1.17 (1.05-1.29) [23]	Obs	High	RF not amenable to intervention during pregnancy	NA	Medium	NA	Country specific	High
Older maternal age (>35 years) & parity >3	1.39 (1.19-1.62) [23]	Obs	High	RF not amenable to intervention during pregnancy	NA	Medium	NA	Country specific	High
**Maternal nutritional status:**									
Low zinc intake*	1.15 (0.97-1.35) [24]	RCT	Medium	Zinc Supplementation (inverse is risk estimate)	NA	Medium	NA	Country specific	Low
Maternal anemia	1.63 (1.33-2.01) [25]	Obs	Medium	Iron supplementation	Medium	Medium	No	Country specific	High
Low calcium Intake*	1.23 (0.98-1.56) [26]	RCT	Medium	Calcium supplementation (inverse is risk estimate)	NA	Medium	NA	Country specific	Low
Short height (<145 cm)	1.42 (1.10-1.83) [27]	Obs	High	RF not amenable to intervention during pregnancy	NA	Medium	NA	Country specific & regional	Medium
Low BMI (<18.5 kg/m^2^)	1.32 (1.10-1.57) [28]	Obs	Medium	Nutrition education and balanced protein energy	Medium	Medium	Yes (NEC), No (BPE)	Country specific	High
**Maternal morbidity:**									
Chronic hypertension	1.54 (1.28-1.93) [29]	Obs	High	Antihypertensive drugs	Low	Medium	No	Regional	Low
Fetal characteristics:									
Fetal gender (male)	1.06 (1.04-1.07) [30]	Obs	Medium	RF not amenable to intervention during pregnancy	NA	Low	NA	Global	Low
Twin pregnancy	3.65 (not reported) [31]	Obs	Medium	RF not amenable to intervention during pregnancy	NA	Low	NA	Regional	Low
**Environmental exposures during pregnancy:**									
Ambient air pollution	1.11 (1.03-1.19) [32]	Obs	Medium	Residential green and blue space	Low	Medium-Low	Yes	Country specific	Medium
Indoor air pollution	1.30 (1.06 -1.59) [33]	Obs	Medium	Cookstoves	Low	Medium-Low	Yes	Country specific	Low
Intimate partner violence	1.89 (1.43-2.48) [34]	Obs	Medium	Integrated cognitive behavioral intervention	Low	Medium-Low	Yes	Country specific & regional	Medium
Tobacco smoking	1.27 (1.21-1.32) [35]	Obs	Medium	Smoking cessation programs	Low	Medium-Low	Yes	Regional	Low
**Uterine, placental and cervical factors:**									
Short cervical length (<25mm)	6.19 (3.84-9.97) [36]	Obs	Low	Pessary	Low	Low	Yes	Global	Low
**Pregnancy related morbidity:**									
Gestational diabetes	1.42 (1.15-1.77) [37]	Obs	Medium	Insulin, diet and exercise, anti-diabetic medications	Medium	Medium	No	Regional	Low
Pre-eclampsia	1.89 (1.73-2.06) [29] reduced to 1.40 (1.32-2.02)^c^	Obs	Medium	Calcium supplementation	Medium	Medium	Yes	Regional	Low
**Maternal infection:**									
HIV	1.49 (1.43-1.55) [38]	Obs	Medium	ARV	Low	Medium-Low	No	Country specific	High
Malaria	1.56 (1.28-1.90) [8]	Obs	High	Iptp	Low (LBW as surrogate outcome)	Medium	Yes	Country specific	Medium
Syphilis	3.22 (3.15-3.23) [39]	Obs	Medium	Penicillin	Medium	Medium	Yes	Regional^b^	Low
Chlamydia	1.26 (1.15-1.38) [40]	Obs	Medium	Erythromycin	Low	Medium-Low	No	Regional	Low
Asymptomatic bacteriuria	1.96 (1.45-2.77) [41]	Obs	Low	Screen and treat	Low	Low	No	Country specific	Medium
Periodontal Infection	1.61 (1.33-1.95) [42]	Obs	Medium	Varying interventions	Medium	Medium	No	Country specific	Medium
Bacterial vaginosis	1.64 (1.36-2.33) [43]	Obs	Medium	Screen and treat	Low	Medium-Low	No	Country specific & regional	Medium
Group B Strep colonization*	1.21 (0.99-1.48) [44]	Obs	Medium	Penicillin	Low	Medium-Low	No	Regional	Low

ARV – antiretrovirals, BMI – body mass index, BPE – balanced protein energy, IPTp – intermittent preventative treatment for malaria in pregnancy, HIV – human immunodeficiency virus, LBW – low birth weight, NEC – nutrition education and counseling, RF – risk factor

*Risk factors that are in the extended model, only: a – for full descriptions of original studies and quality assessment, please see supplementary materials; b – prevalence was adjusted for treatment effects; c – reduced because of relationship with calcium deficiency. See supplementary materials for more details.

### Estimates of spontaneous preterm births in each country

We used the country-specific estimates of births from 2019 and PTB rates from 2010 to estimate the number of PTB in each country [45,46]. We used PTB rate estimates from 2010 because more recent national estimates were available for only 16 of the 81 countries included in our analysis [3]. sPTB estimates in each country were calculated accounting for regional differences in the proportion of preterm births that are spontaneous vs provider-initiated [8].

### Calculation of population attributable fractions

The population attributable fraction describes the proportional reduction in the outcome variable (in this case, sPTB) in the overall population if the those with a risk factor (for example, gestational diabetes) are shifted into the non-risk category (eg, no gestational diabetes) [47]. The risk relationships that were reported as odds ratios were converted to risk ratios for the following calculations [48]. Our approach to estimating the contribution to sPTB of individual risk factors consisted of six steps (**Box 1**). First, we used Equation 1 (Levin’s formula) [PAFu] to estimate the population attributable fraction for each of the risk factors for each country [49,50]. Second, we used Equation 2 [PAFt] to produce a combined estimate of the population attributable fraction for all risk factors in that country, which accounts for the presence of multiple risk factors [51]. Third, we applied Equation 3 [PAFa] to normalize the individual risk factor PAFs and produce our final estimate of the corrected population attributable fractions for each country. Using these PAFs, we estimate the number of sPTB in each country associated with a risk factor (Equation 4), and then summed these values for all 81 LMICs and for subsets of countries within each of the two regional groupings. To obtain the final estimate of the percent of sPTB attributable to each risk factor in the included 81 LMICs and in the two regions, we divided the total number of PTBs for each risk factor by the total number of sPTB. This process was completed for both the final model (24 risk factors) and the extended model (27 risk factors). The country specific PAF calculations are run for the final model only.

Box 1 **Methods for estimating the population attributable risk for PTB risk factors**

Equation 1: Individual PAF. This formula is used to estimate the independent PAF for each factor. It does not correct for multiple risk factors and the sum of the risk for the 26 factors can be above 100%.

For all risk factors:

PAF_u_ = [P(F)(RR – 1)] / [1 + P(F)(RR – 1)],

where P(F) is the risk factor prevalence and RR is the relative risk associated with the factor, comparing preterm birth in the exposed and unexposed groups.

Equation 2: This formula corrects for multiple factors and yield a combined PAF for the 26 risk factors that cannot exceed 100%.

PAF_t_ = 1 – Π^26^ (1 – PAF_u_)

u = 1

Equation 3: This equation normalizes the PAF of individual risk factors to the combined PAF from equation 1.

PAF_a_ = (Individual PAF_u_ / ∑ PAF_u1-26_) × PAF_t_)

Equation 4: This equation translated rates into estimates of the number of spontaneous PTB linked to each risk factor.

#PTB associated with each risk factor_country_ = PAF_a_ × # spontaneous PTB_country, 2010_

## RESULTS

### Risk associations

**Figure 1:**
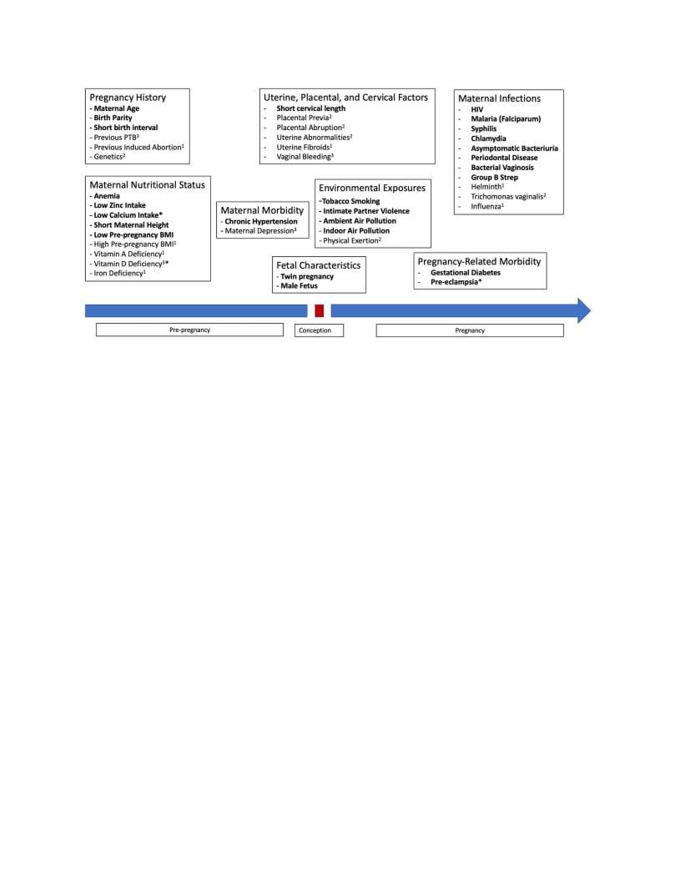
Potential risk factors for preterm birth. Risk factors in **bold** are included in the models. Other factors were reviewed and excluded due to: 1 = Evidence of no risk; 2 = Insufficient evidence; 3 = Not an independent risk factor. The asterisk indicates there are linked risk factors, ie, calcium is included in the model but vitamin D is not; the risk of preterm birth for preeclampsia has been adjusted for the impact of calcium supplementation. Details for all risk factors are available in the supplementary appendix. Excluded from diagram: biomarkers (fetal fibronectin, genetics).

**Figure 1** summarizes the results of our investigations of factors proposed by previous research as potential risk factors for sPTB. Seventeen of these risk factors were excluded from the analysis because of reasons described in the Methods section. The supplementary materials provide summaries of available evidence for all 44 potential PTB risk factors (Appendix S2 in **Online Supplementary Document**).

**Table 1** shows the relative risks for sPTB for the 27 risk factors included in the final and extended models (three additional risk factors in the extended model marked with an *). The quality of evidence supporting the risk estimates, estimates of risk factor prevalence, and quality of the prevalence data sources are presented for each risk factor. The estimated relative risks of these 27 factors for PTB range from 1.06 (for male children) to 6.19 (for cervical length <25mm).

Our reviews indicated that three risk factors are related to sPTB, with significant overlap: low calcium intake, low vitamin D intake, and preeclampsia. Previous treatment trials examining calcium supplementation and vitamin D supplementation, administered separately, have shown only calcium supplementation to significantly reduce the risk of preeclampsia and PTB [26,52]. Therefore, low vitamin D intake was excluded from both models. When the trials are limited to those in settings with low calcium intake, the effect of supplementation on preterm births is no longer significant [26]. Therefore, this risk factor was only accounted for in the extended model that includes risk factors with medium or high-quality evidence, but borderline insignificant associations with the outcome. We reduced the relative risk of preeclampsia from 1.89 to 1.32 to reflect the fact that calcium supplementation reduces the risk of preeclampsia by 55% [26]. For the extended PAF model we used one risk for low calcium intake and for both the final and extended models we included a second risk for preeclampsia independent of low calcium intake. In addition, the risks due to maternal age and parity were considered together as three categories, based on data showing the risk for these combinations of factors [23].

Highest relative risks (>2.0) are associated with short cervical length, twin pregnancies and maternal syphilis infection. There were no risk factors with an overall high-quality score.

### Risk factor prevalence

Country-specific data on the prevalence of risk factors were available for about half of the risk factors (15 of 27), though not all of these were for women of reproductive age or pregnant women, and the quality of prevalence data varied widely across risk factors.

### Population attributable fraction of sPTB

**Table 2** shows the estimated percent of sPTB attributed to each risk factor for the 81 Countdown countries for both the final and extended models. The leading risk factors included in the final model are maternal anemia (13.1%), indoor air pollution (9.7%), bacterial vaginosis (9.5%), chronic hypertension (4.9%) and maternal age >35 years and parity >3 (3.3%). These estimates reflect the population-attributable fraction (PAF) for each risk factor, considering the other risk factors. The extended model has the same three leading risk factors, but low calcium intake (6.9%) replaces chronic hypertension for the fourth highest PAF. The total PAF across the 81 countries in the final model is 73%, compared to 78.1% in the extended model.

**Table 2:** Estimated percent of preterm births attributed to risk factors for all 81 LMICs, in the final and extended models

	Final model PAF (Min-Max)	Extended model PAF (Min-Max)
**Pregnancy history**		
Short birth interval	1.96 (0.70-4.06)	1.81 (0.63-3.70)
Young maternal age (<18 years) & primiparity	1.24 (0.23-3.65)	1.13 (0.22-3.30)
Maternal age 18-35 years & parity >3	3.32 (1.39-5.12)	3.06 (1.26-4.98)
Older maternal age (>35 years) & parity >3	2.15 (0.72-3.66)	1.98 (0.67-3.44)
**Maternal nutritional status:**		
Low zinc intake	N/A	2.01 (0.90-2.51)
Maternal anemia	13.07 (5.99-17.20)	12.05 (5.46-15.52)
Low calcium intake	N/A	6.96 (0.07-10.88)
Short height (<145 cm)	1.50 (0.05-5.89)	1.39 (0.04-5.50)
Low BMI (<18.5 kg/m^2^)	2.96 (0.52-4.49)	2.73 (0.48-4.16)
**Maternal morbidity**		
Chronic hypertension	4.86 (3.43-7.32)	4.47 (3.21-7.02)
**Fetal characteristics:**		
Twin pregnancy	2.19 (1.66-5.18)	2.02 (1.51-4.95)
Fetal gender (male)	1.72 (1.54-2.05)	1.59 (1.41-1.96)
**Environmental exposures during pregnancy:**		
Indoor air pollution	9.72 (0.17-14.32)	8.94 (0.16-13.01)
Ambient air pollution	3.17 (0.73-4.51)	2.93 (0.66-4.09)
Intimate partner violence	2.95 (1.83-4.64)	2.73 (1.65-4.40)
Tobacco smoking	0.16 (0.01-1.48)	0.15 (0.01-1.42)
**Uterine, placental, and cervical factors**		
Short cervical length (<25mm)	2.22 (1.98-2.63)	2.04 (1.81-2.52)
**Pregnancy-related morbidity:**		
Gestational diabetes	2.53 (1.63-3.50)	2.33 (1.51-3.27)
Pre-eclampsia	0.72 (0.28-1.14)	0.66 (0.27-1.04)
**Maternal infections:**		
HIV	0.42 (0.00-6.85)	0.38 (0.00-6.19)
Malaria	1.92 (0.00-9.91)	1.75 (0.00-9.05)
Syphilis	1.03 (0.24-2.34)	0.95 (0.22-2.12)
Chlamydia	0.47 (0.27-1.32)	0.43 (0.25-1.25)
Asymptomatic bacteriuria	0.07 (0.04-0.20)	0.06 (0.04-0.20)
Periodontal Infection	3.13 (0.32-5.68)	2.90 (0.29-5.32)
Bacterial vaginosis	9.53 (8.03-11.70)	8.77 (7.29-11.23)
Group B Strep colonization	N/A	1.84 (1.21-2.82)
**Total**	73.0%	78.1%

BMI – body mass index, HIV – human immunodeficiency virus, PAF – population attributable fraction

In sub-Saharan Africa (SSA), the 24 risk factors in the final model have a total PAF of 77.1%, with the top four risk factors like those identified at global level: maternal anemia (12.4%), indoor air pollution (11.3%), bacterial vaginosis (10.0%), chronic hypertension (6.3%) and malaria (4.5%). Similar to the overall extended model, when low calcium intake was included for sub-Saharan Africa it replaced chronic hypertension as the fourth leading risk factor and the total PAF increases to 82.1%. Malaria had a larger PAF in SSA compared to the overall estimate, with a PAF of 4.6% compared to 1.9%.

In the five-country South Asia group, the total PAF for the 24 risk factors was equal to 72.4%. The top five risk factors are maternal anemia (14.9%), indoor air pollution (9.2%), bacterial vaginosis (9.1%), periodontal infection (4.5%) and low body mass index (BMI) (4.2%). The contribution of periodontal disease is greater in this region than SSA (4.5% PAF vs 1.9% PAF, respectively). In the extended model, low calcium intake is again the fourth greatest PAF (5.6%) and the total PAF is 76.8%.

**Figure 2:**
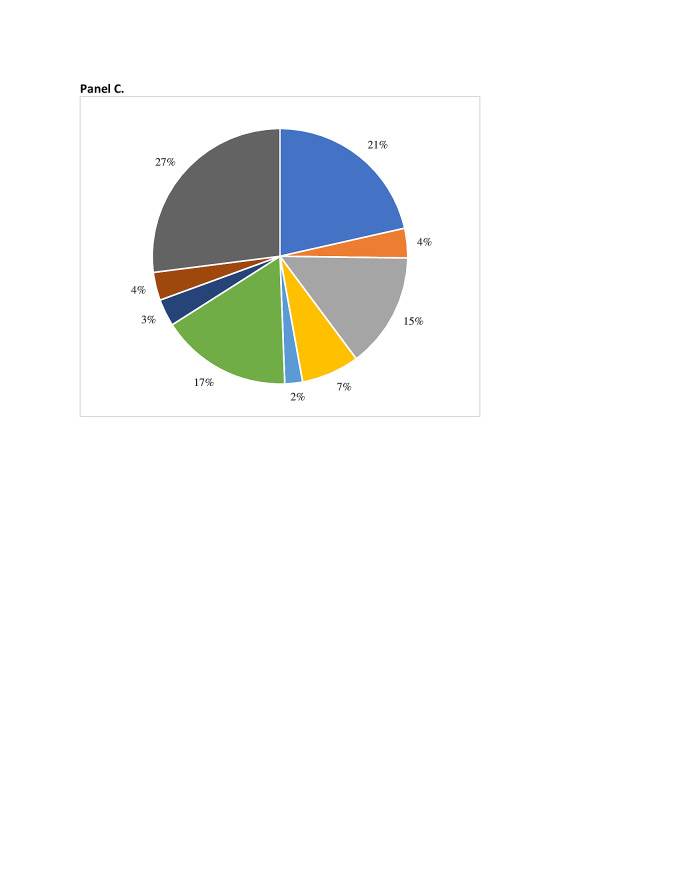
Percentage of sPTB by risk factor group, overall and by region. **Panel A.** 81 LMIC. **Panel B.** Sub-Saharan Africa. Panel C. South Asia.

Using the eight risk factor groupings shown in **Figure 1**, in the final model maternal nutritional risk factors have the greatest PAF (17.5%) across the 81 countries. The second highest contributing group is maternal infections, with a PAF of 16.6%, followed by environmental exposures during pregnancy (16.0%), pregnancy history (8.7%), maternal morbidity (4.4%), fetal characteristics (3.9%), pregnancy-related morbidities (3.3%), and cervical insufficiency (2.2%). In the South Asia region, the order of risk factor groupings is the same as the overall 81 countries. The nutritional status grouping in South Asia is associated with the greatest percentage of sPTB (21.5%) of any of the risk groupings in the final model. In the final model for sub-Saharan Africa, the two highest contributing groupings are maternal infections (19.7%) and environmental factors (15.5%). The nutritional status risk factor grouping drops to third with a total PAF equal to 15%. However, in the extended model, the nutritional status group has the greatest PAF in the SSA region (23.3%). The percentage of sPTB represented by these eight groups overall and in the two regions are presented in **Figure 2**, panels A-C.

**Figure 3:**
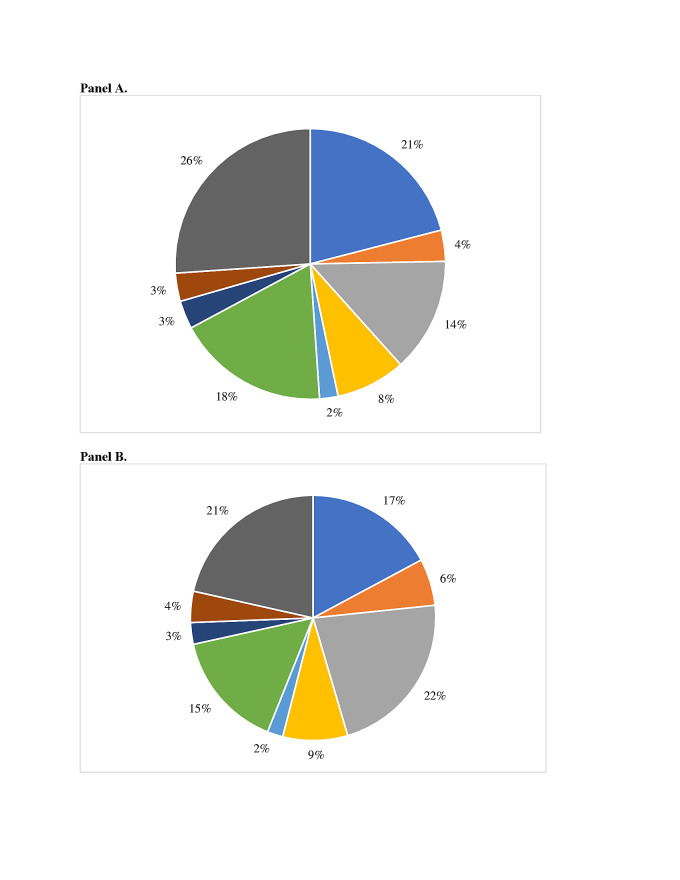
Percentage of sPTB by risk factor group, example countries. **Panel A.** Bangladesh. **Panel B.** Ghana.

Risk factors for sPTB also vary by country. **Figure 3**, panels A and B, present country-specific sPTB risk factor group profiles Ghana and Bangladesh to illustrate this variation. The PAF for the nutritional status group is greater in Bangladesh (21%) than in Ghana (17%). The maternal infection group had a greater PAF in Ghana (22%) than Bangladesh (14%). The total PAF for the pregnancy history and pregnancy-related morbidity were similar between the two countries.

## DISCUSSION

Our analysis used available evidence to estimate the relative importance of risk factors for sPTB in LMIC by estimating their population attributable fractions. The analysis identified 24 sPTB risk factors that are currently supported by evidence, which together (and excluding overlap) totaled to a PAF of 73.0% across the 81 Countdown countries. The extended model that included three additional risk factors (low zinc intake, low calcium intake and group B streptococcus infection) for a total of 27 risk factors accounted for 78.1% of sPTB. Differences between regions and countries are important as a basis for prioritizing interventions. For example, in South Asia, where a high proportion of sPTB are associated with non-infectious risk factors (nutritional deficiencies, indoor air pollution, short maternal height, low pre-pregnancy BMI), family planning, economic and nutritional interventions throughout the life course may be more effective. In sub-Saharan Africa, focusing on screening and treatment of maternal infections could result in a greater reduction in sPTB. The ranking of risk factor contribution and the demonstrated diversity of risk factors should be considered when designing future programmatic efforts.

No single risk factor had a PAF greater than 15% of sPTB in these LMICs. Instead, we identified multiple risk factors, most having PAFs of less than 5%. This finding has important implications for accelerating efforts to reduce sPTB and to achieve the SDGs, suggesting that scaling up one or two interventions to reduce the associated risk factors will not have a major impact on global rates of preterm birth. Additionally, in the final model 27% of the PAF for sPTB is unaccounted for, indicating that there are other risk factors that contribute to sPTB that we were unable to include due to unavailability of evidence in the LMIC setting, unavailability of prevalence estimates or inconclusive evidence from studies published thus far.

Our findings show PAF for some of the 24 sPTB risk factors that are much lower than those reported previously. The previously published PAFs reflect studies that focused on the contribution of a single risk factor, whereas our analytic approach estimates the contribution of each risk factor while taking others into account. This correction results in lower PAF estimates, in some cases drastically so. Additionally, in some of the prior studies, the study populations may not have been representative of the general population due to the focus on a single risk factor, which may result in high prevalence estimates. Furthermore, while many observational studies control for some potential confounders, the resulting relative risks may overestimate the association with preterm birth because of unmeasured or underestimated coexisting risk factors.

Maternal-Fetal infections are often cited in the literature as contributing to 30%-40% of preterm births [6,53-55]. The PAF for maternal infections included in this analysis (Bacterial vaginosis, UTI, Chlamydia, Syphilis, Group B Streptococcus, HIV, syphilis, periodontal disease) jointly contributed to 16.6% of sPTB. This is primarily due to previous studies focusing on single risk factor while this analysis looked at 24 (or 27) risks factors at once. There is a limitation to the analysis as due to the lack of prevalence data on chorioamnionitis, or intrauterine infection, certain TORCH infections and other bacterial or viral infections. Preterm premature rupture of membranes may be associated with sub-clinical intrauterine infection, and we did not have adequate prevalence data to estimate its contribution either.

The comparison to previously published PAFs reveals that our estimates are consistently lower. This is not unexpected; as discussed previously, this is likely due to the consideration of the presence of all other risk factors in our estimate, which are unaccounted for in the other estimates. The estimated PAF for short maternal height (<145cm) was within the range of those previously estimated; 1.5% compared the previously estimate range of 0.7%-7.9% [27]. The estimates for malaria are lower than those previously cited; our estimate of malaria in sub-Saharan Africa was 4.5% compared to a previous estimate in sub-Saharan African countries of 8%-36% [56]. This could be due to the prevalence estimate being adjusted for IPTp coverage, which would result in a lower prevalence and a lower PAF. Additionally, our prevalence estimates may be lower than older studies because of concerted efforts in reducing malaria prevalence over time, which in turn lowers our PAF estimate. Other estimates of short birth interval include a range of 3.3%-6.6%, [22] and 3.9% for moderately preterm birth (33-36 weeks gestational age), and 6.1% for extremely preterm birth (24-32 weeks gestational age) [57], compared to our estimate of 1.9%. The maternal anemia estimate from this analysis (13.1%) is lower than the previously reported PAF value of 19% [25]. The PAF for anemia, which can be the result of numerous deficiencies and infections, may be lower in our analysis because we accounted for some of these sources. Tobacco smoking during pregnancy was cited as being responsible for 4% of PTB, but in this analysis the PAF was only 0.16% [58]. This difference may be due to the lower prevalence of maternal tobacco use in LMICs (average 3.4%) compared to the study in California in the 1970s that reported a prevalence of 13%. The PAF from this model for ambient air pollution (18% vs 3.1%), [59] twin pregnancy (15%-20% vs 2.1%), [6] and cervical insufficiency (14.6%-16.3% vs 2.2%) [60] are all also well below previously cited PAFs.

The same analytical process was conducted for the birth outcome “small for gestational” (SGA) by the team (results forthcoming). The same number of risk factors (N = 27) were included in the final model for both sPTB and SGA, though the included risk factors differed. The total PAF for the SGA final model (63.9%) was lower than the sPTB final model (73.0%). Indoor air pollution was the second leading risk factor in both analyses. Additionally, for both sPTB and SGA, maternal nutritional status was the leading risk factor grouping. This suggests that improving maternal nutritional status through evidence-based interventions and addressing indoor air pollution would have an impact on reducing the incidence of both birth outcomes.

### Limitations of the analysis

In LMICs, there is a general dearth of available data on gestational age, particularly availability of high-quality gestational age dating based on an early pregnancy ultrasound. Inaccuracy in gestational age dating results in misclassification of preterm births, shown to overestimate the proportion of births that are preterm [61], which affects both preterm birth prevalence estimates and risk associations. On the other hand, inclusion of births at 38-39 weeks that have higher increased complications compared to births at 40 weeks would increase the number of sPTB; little information is available to say if risk factors for these births are the same as those for births <37 weeks.

The use of a simple literature review for the individual risk factor investigations is a limitation of the analysis, although this was done because a systematic review of this number of risk factors was not feasible. To ameliorate this, we developed a quality scoring system with a preference for articles given to those that were a result of systematic reviews, such as Cochrane reviews or other meta-analyses. The year of data collection and publication, number of studies included in a meta-analysis (if applicable), study design and many other factors were also considered when selecting the articles used for risk and prevalence estimates (full explanations can be found in the Appendix S3 of the **Online Supplementary Document**).

Another limitation is the paucity of country-specific prevalence data for many of the risk factors. This resulted in having to use modeled or regional prevalence to calculate the country-specific PAFs. Both modeled estimates and applying regional prevalence estimates to a specific country rely on assumptions and are imperfect measures. Ideally, there would be country-level estimates of all the present risk factors, but this is not the case.

Finally, although the estimation method we used accounts for the presence of other risk factors, this method relies on the assumption that the relative risk estimate has been adjusted for all other risk factors [51]. This is rarely, if ever, possible though because of the resources necessary and the presence of unknown risk factors. Therefore, the PAFs we present here may still be overestimating the true attributable fraction but offer a more realistic estimate than those studies examining a single risk factor.

### Additional considerations

For this analysis, very few relationships between risk factors and sPTB that were examined by a RCT of an intervention; low calcium intake and low zinc intake were the primary ones and these are only included in the extended model. Therefore, there are very few risk factors where a causal conclusion can be made, which limits our confidence in the risk relationships presented. However, for many risk factors, a randomized trial is not feasible and methods, such as Mendelian randomization [62], to approximate a causal relationship from observational studies have not often been conducted in the LMIC settings. Although most of the estimated risk ratios use adjustment in their analyses, there is still a possibility for residual confounding. The estimates of the RR for these factors and the resulting PAF presented here should be thought of as closer to the upper bounds on the estimates of PAF for the risk factors.

We did our best to detangle causal pathways and eliminate those risk factors in the causal pathway of another (full explanations can be found in Appendix S2 of **Online Supplementary Document**) given the existing literature, however for some risk factors the true pathways are still known to the medical community. For example, the highest contributing risk factor to sPTB is maternal anemia, and the biological links between anemia and sPTB are also not yet fully understood. Anemia can be caused by a variety of nutrient deficiencies (predominantly iron), infectious diseases including malaria, inflammation, and genetic disorders, and the causes of anemia differ by population and region. Furthermore, iron deficiency is the leading contributor to anemia and yet iron supplementation alone has not been shown to impact preterm birth [63]. Given the current body of evidence, it is still unclear which of these causal pathways links anemia to sPTB [25]. As discussed in the results, the mechanisms between pre-eclampsia, low calcium intake and low vitamin D intake are not well understood.

Another limitation is that our analysis assumes that each woman has only one sPTB risk factor, when many if not most women are exposed to more than one risk. For example, a woman with a low BMI may also be zinc deficient, of short stature, or have an infectious disease, all with associated risks for sPTB. This analysis takes a first step by clarifying a set of sPTB risk factors that are supported by existing evidence and estimating their singular contributions; the next steps are to delineate the interrelationships among risk factors and their epidemiological co-occurrence, as well as possible additive/multiplicative/synergistic or antagonistic effects.

### Implications for research, development and implementation agendas

Following the Millennium Development Goal (MDG) era, there was a call for improved data systems in LMICs in order to improve the timely and accurate measurement of coverage for maternal, newborn, and child interventions [64]. Our experience in conducting this analysis suggests that this continues to be an urgent and unmet need. Even though complications associated with preterm birth are the largest contributor to under-five and neonatal mortality globally, there are few high-quality, country-specific data on the prevalence of risk factors for preterm birth or studies with preterm birth as a primary outcome. The challenges of measuring gestational age in LMICs are real, but greater effort is needed to overcome this challenge and generate increased quantity and quality of data on gestational age. Improved measurement is needed not only for clinical care, but also to increase the availability of epidemiologic data to support more precise estimation of PAF for PTB risk factors in specific countries and therefore more accurate targeting of interventions to areas of higher risk.

There are risk factors for spontaneous preterm birth identified here that are amenable to interventions to reduce the risk factor during pregnancy, some of which already exist. There are efficacious interventions for many of the risk factors (IPTp for malaria, multiple micronutrient supplementation for anemia, penicillin for syphilis and various antibiotics for infections), but coverage for many of these interventions is very low in many LMIC. The 2010 *Global Report on Preterm Birth and Stillbirth* identified only two interventions (smoking cessation programs and progesterone) for preterm birth that fit their criteria for “high” quality interventions, where additional research is unlikely to change recommendations [10]. Additionally, there are promising interventions, such as aspirin [65], that have been shown to be efficacious in trial settings but have not yet been implemented globally. Another note, this analysis focused on interventions provided as treatment during pregnancy. Therefore, there are additional opportunities for preventative intervention that fall outside the scope of this analysis, such as population-based interventions (ie, policy surrounding fortification and supplementation) that may be impactful.

For many risk factors, there remains limited evidence supporting interventions and/or therapeutics that can effectively reduce the incidence of preterm birth at scale. A prime example of this is bacterial vaginosis (BV). While BV and vaginal dysbiosis have been associated with preterm birth in numerous epidemiologic studies, several intervention trials have repeatedly failed to show an impact of current methods of screening for and treating BV to prevent preterm birth in various geographic settings [66-69]. Further research and discovery related to the biological pathways by which these risk factors may lead to preterm birth are needed to develop effective interventions to both treat the risk factor and prevent preterm birth.

The extended model was developed to assess the potential contribution of that set of risk factors that have a borderline association with sPTB. We did not want to discount these risk factors entirely because other studies have demonstrated a significant risk relationship with preterm birth that was nullified in the current study by an inclusion of a single study or disaggregation of results with a limited number of trials. Their inclusion in the extended model did increase the proportion of sPTB associated with a risk factor, particularly for low calcium intake. Further trials examining calcium supplementation in LMIC would be potentially beneficial for reducing sPTB. In the 2018 Cochrane review by Hofmeyr et al., the aggregated analysis demonstrates a reduction in preterm birth, but when disaggregated into normal and low calcium intake settings, calcium supplementation is no longer significant in the low intake group [26]. This could be attributed to insufficient power, as the point estimates for the two analyses are the same, or other methodological factors. Clarifying calcium supplementation’s impact in this setting would be beneficial, because low calcium intake is the fourth leading risk factor associated with sPTB in the extended model. The situation with zinc is similar in that a revised Cochrane review of antenatal supplementation changed the finding of a statistically significant 14% reduction in PTB to a 13% reduction with the upper bound of uncertainty just crossing one [24].

## CONCLUSIONS

This analysis takes a first step by clarifying a set of sPTB risk factors that are supported by existing evidence and estimating their singular contributions. There are risk factors that develop and can be addressed during pregnancy and there are also risk factors that reflect lifelong and even inter-generational issues. There are risk factors for which the intervention needs to target the individual woman, and risk factors for which the intervention needs to target households and communities. Additionally, as our results demonstrate, many of these risk factors have a PAF less than 5% and many pregnant women may have multiple risk factors. Therefore, intervention trials on a package of interventions addressing multiple causes may be an alternative to fully untangling the biological pathways and would focus more on examining future opportunities for prevention in programmatic planning to reduce preterm birth incidence. This also supports the call for health systems strengthening and improving access to and quality of routine antenatal care services to deliver the necessary screening procedures and interventions. The multiplicity of risk factors and their geographic variability means that an approach focused on a single risk factor is not the answer to reducing the burden of preterm birth and achieving the child survival SDG.

## Additional material


Online Supplementary Document

## Figures and Tables

**Table 3:** Estimated percent of preterm births attributed to risk factors for Sub-Saharan region and South Asia region*, in the final and extended models

	Sub Saharan Africa		South Asia	
	**Final model PAF (Min-Max)**	**Extended model PAF (Min-Max)**	**Final model PAF (Min-Max)**	**Extended model PAF (Min-Max)**
**Pregnancy history:**				
Short birth interval	1.54 (0.70-4.06)	1.40 (0.63-3.70)	2.41 (1.32-3.60)	2.26 (1.19-3.48)
Young maternal age (<18 years) & primiparity	1.50 (0.48-2.60)	1.37 (0.44-2.35)	1.03 (0.59-3.65)	0.95 (0.57-3.30)
Maternal age 18-35 years & parity >3	3.90 (1.42-4.85)	3.56 (1.32-4.40)	2.90 (1.45-4.35)	2.72 (1.32-4.21)
Older maternal age (>35 years) & parity >3	2.88 (1.22-3.56)	2.63 (1.13-3.26)	1.01 (0.72-2.26)	0.95 (0.67-2.19)
**Maternal nutritional status:**				
Low zinc intake	0.00 (0.00-0.00)	1.88 (1.78-2.14)	0.00 (0.00-0.00)	2.19 (1.44-2.46)
Maternal anemia	12.40 (5.99-16.17)	11.30 (5.46-14.58)	14.87 (13.24-15.16)	13.88 (12.26-14.08)
Low calcium intake	0.00 (0.00-0.00)	7.72 (1.01-9.87)	0.00 (0.00-0.00)	5.56 (0.93-9.54)
Short height (<145 cm)	0.52 (0.05-1.69)	0.47 (0.04-1.52)	2.41 (1.15-2.95)	2.23 (1.12-2.67)
Low BMI (<18.5 kg/m^2^)	2.11 (1.02-3.59)	1.92 (0.92-3.27)	4.20 (2.50-4.49)	3.91 (2.28-4.16)
**Maternal morbidity:**				
Chronic hypertension	6.27 (5.93-7.12)	5.71 (5.41-6.50)	3.74 (3.43-4.03)	3.49 (3.32-3.67)
**Fetal Characteristics**				
Twin pregnancy	2.55 (2.41-2.89)	2.32 (2.20-2.64)	1.81 (1.66-2.28)	1.69 (1.51-2.20)
Fetal gender (male)	1.63 (1.54-1.85)	1.48 (1.41-1.69)	1.75 (1.71-1.85)	1.63 (1.55-1.68)
**Environmental exposures during pregnancy:**				
Indoor air pollution	11.30 (2.73-13.15)	10.29 (2.48-11.90)	9.17 (6.63-11.17)	8.56 (6.04-10.12)
Ambient air pollution	2.60 (0.88-4.18)	2.37 (0.79-4.01)	4.05 (3.85-4.51)	3.78 (3.51-4.09)
Intimate partner violence	3.09 (2.92-3.51)	2.82 (2.67-3.20)	2.73 (2.39-3.97)	2.56 (2.17-3.84)
Tobacco smoking	0.09 (0.01-0.67)	0.08 (0.01-0.61)	0.08 (0.02-0.88)	0.08 (0.01-0.82)
**Uterine, placental, and cervical factors:**				
Short cervical length (<25mm)	2.10 (1.98-2.38)	1.91 (1.81-2.17)	2.25 (2.20-2.38)	2.10 (1.99-2.17)
**Pregnancy-related morbidity**				
Gestational diabetes	2.02 (1.91-2.29)	1.84 (1.74-2.09)	2.86 (2.75-3.06)	2.67 (2.49-2.96)
Pre-eclampsia	0.88 (0.83-1.00)	0.80 (0.76-0.91)	0.58 (0.28-0.68)	0.53 (0.28-0.62)
**Maternal infections:**				
HIV	0.97 (0.03-6.85)	0.88 (0.03-6.19)	0.02 (0.00-0.03)	0.02 (0.00-0.03)
Malaria	4.46 (0.00-9.91)	4.06 (0.00-9.05)	0.08 (0.00-0.11)	0.08 (0.00-0.10)
Syphilis	1.78 (1.54-2.34)	1.62 (1.40-2.12)	0.50 (0.35-0.63)	0.46 (0.32-0.61)
Chlamydia	0.53 (0.50-0.61)	0.49 (0.46-0.55)	0.33 (0.27-0.54)	0.31 (0.25-0.52)
Asymptomatic bacteriuria	0.05 (0.04-0.08)	0.05 (0.04-0.08)	0.08 (0.06-0.08)	0.07 (0.06-0.08)
Periodontal infection	1.88 (0.32-4.65)	1.71 (0.29-4.26)	4.47 (2.69-4.91)	4.17 (2.50-4.55)
Bacterial vaginosis	10.02 (9.48-11.39)	9.13 (8.65-10.39)	9.12 (8.24-9.86)	8.51 (7.98-8.98)
Group B Strep colonization	0.00 (0.00-0.00)	2.29 (2.17-2.60)	0.00 (0.00-0.00)	1.39 (1.21-1.95)
**Total**	77.1%	82.1%	72.4%	76.8%

BMI – body mass index, HIV – human immunodeficiency virus, PAF – population attributable fraction

*Bangladesh, Bhutan, India, Nepal, Pakistan.
