# Supplementary material for: Population attributable fractions for risk factors for spontaneous preterm births in 81 low- and middle-income countries: A systematic analysis
Source: J Glob Health. 2022 Mar 26;12:04013. doi: 10.7189/jogh.12.04013 (PMC8959104; doi:10.7189/jogh.12.04013)
Supplement: Online Supplementary Document [file jogh-12-04013-s001.pdf]

Supplementary materials

Overview of the Approach to Estimating the Population Attributable Fractions for Causes of Preterm Births

Table of Contents

1. General Overview ***2***

Table 1. Included risk factors in final (Model 1) and extended (Model 2) models ***5***

Table 2. Excluded Risk Factors ***7***

Table 3. Weighted prevalence estimates by WHO region ***9***

Quality Assessment ***12***

Table 4a. Observational quality checklist **12**

Table 4b. Quality checklist for intervention **13**

Table 4c. Overall quality of risk estimates **15**

2. Risk Estimate Sources and Quality ***16***

Pregnancy History **16**

Maternal Nutritional Status **19**

Maternal Morbidity **24**

Fetal characteristics **25**

Environmental Exposures during Pregnancy **27**

Uterine, Placental, and Cervical Factors **32**

Pregnancy-Related Morbidity **36**

Maternal infections **38**

Other **46**

3. Sources and Quality of Prevalence Estimates ***46***

4. Search Strategies ***55***

Table 4. Country Estimates ***56***

References ***66***

1. **General Overview**

The aim of this analysis is to estimate the contribution of individual risk factors to the overall risk of preterm birth in low-and-middle-income countries. We prioritized sPTB because three-quarters of preterm births in LMICs are spontaneous.(1) iatrogenic an identified potential risk factors for all sPTB presented in two recent syntheses developed by experts in the field, [4, 6] and confirmed by other analyses of PTB risk factors in the published literature.[2-9] A separate search was run to identify other possible risks not included in these publications

Once the risk factors were identified, a literature review was conducted to find the appropriate sources for the corresponding risk. When possible, meta-analyses with the most recent data published were used to estimate risk, with a priority given to Cochrane reviews. Additionally, priority was given to meta-analyses that included studies primarily conducted in low- and middle-income countries (LMICs). However, for some of the risk factors, this was not always possible while also citing the most rigorously collected data. Risks reported as an odds ratio were converted to relative risks for the population attributable fraction calculations.[10] For these purposes, we are only considering interventions that are implemented while the woman is pregnant, even though there are interventions to address some of the risk factors at earlier life stages.

There are also risk factors that have been identified but were excluded from the models for one of the following reasons: the risk factor not an independent risk factor for spontaneous preterm birth and is accounted for through other risk factors in the model, there is insufficient data on the risk factor (risk relationship and/or prevalence) or, finally, evidence of no risk relationship.

These are included for discussion in the risk estimate portion of the supplementary materials but are not included in the model.

The quality of the evidence for each risk factor was assessed using a checklist that was developed by referencing PRISMA and GRADE guidelines. For risk factors that have interventions available for implementation during pregnancy, the source for the intervention evidence was graded as well. The full checklist can be found below the risk factor information sections below. The three quality categories were high, medium and low. High quality evidence earned a score greater than ten for risk and 13 for intervention (maximum possible score is 14 for risk factors, 17 for interventions), taking into consideration outcome/exposure definition, sample size, generalizability to low- and middle-income countries, heterogeneity, methods of addressing biases and potential for confounding and magnitude of effect. A score greater than or equal to eight and less than or equal to ten was categorized as medium quality evidence for risk relationships and between ten and 13 for interventions. Low quality evidence had a score below eight for risk relationship and below ten for interventions. A final quality score is listed, which considers both the risk factor and intervention evidence.

For the prevalence data, when possible country specific estimates were used from DHS surveys, or those developed by groups such as IHME, UNAIDS, or the WHO. When country-specific data were unavailable, regional data were used. In three instances global data was the only prevalence source available. The preterm birth rate for each country is from a 2013 publication estimating 2010 rates.[11] There is also a 2019 Lancet Global Health paper, which presents a combination of national level and regional level estimates for preterm birth rates in 2014, depending on the country.[12] The country specific preterm birth rates from 2010 are used in the analyses because in the more recent estimates only 16 of 81 countries included in the analysis had country-specific estimates from 2014; the remaining were regional estimates. Additionally, as our analysis focuses on risk factors for spontaneous preterm birth, the number of preterm births for each country was modified using regional estimates of the proportion of preterm births that are spontaneous versus provider initiated.[1]

Table 1. Included risk factors in final (Model 1) and extended (Model 2) models

Risk Factor	Relative Risk (95% CI)	RR Source	Risk Quality (Score)	Intervention	Intervention Quality (Score)	Overall quality	Agreement on effects	Prevalence	Prevalence Quality
Pregnancy history									
Short birth interval	1·49 (1·17-1·89)[13]	Obs	High (11.5)	RF not amenable to intervention during pregnancy	NA	Medium	NA	Country specific	High
Young maternal age (< 18 years) & primiparity	1·46 (1·36-1·58)[14]	Obs	High (11.5)	RF not amenable to intervention during pregnancy	NA	Medium	NA	Country specific	High
Maternal age 18-35 years & parity >3	1·17 (1·05-1·29)[14]	Obs	High (11.5)	RF not amenable to intervention during pregnancy	NA	Medium	NA	Country specific	High
Older maternal age (>35 years) & parity >3	1·39 (1·19-1·62)[14]	Obs	High (11.5)	RF not amenable to intervention during pregnancy	NA	Medium	NA	Country specific	High
Maternal Nutritional status									
Low Zinc Intake	1·15 (0.98-1·30)[15]	RCT	Medium (11.5)	NA	NA	Medium	NA	Country specific	Low
Maternal Anemia	1·63 (1·33-2·01)[16]	Obs	Medium (11)	Iron supplementation	Medium (11.5)	Medium	No	Country specific	High
Low Calcium Intake	1·23 (0.98-1·56) [17]	RCT	Medium (10)	NA	NA	Medium	NA	Country specific	Low
Short height (<145 cm)	1·42 (1·10-1·83)[18]	Obs	High (11)	RF not amenable to intervention during pregnancy	NA	Medium	NA	Country specific & regional	Medium
Low BMI (<18·5 kg/m^2)	1·32 (1·10-1·57)[19]	Obs	Medium (10)	Nutrition education/ balanced protein energy	Medium	Medium	Yes/ No	Country specific	High
Maternal Morbidity									
Chronic hypertension	1·54 (1·28-1·93)[20]	Obs	High (11)	Antihypertensive drugs	Low (9)	Medium	No	Regional	Low
Fetal characteristics									

Twin pregnancy	3·65 (not reported) [21]	Obs	Medium (10)	RF not amenable to intervention during pregnancy	NA	Low	NA	Regional	Low
Fetal gender (male)	1·06 (1·04-1·07) [22]	Obs	Medium (8.5)	RF not amenable to intervention during pregnancy	NA	Low	NA	Global	Low
Environmental exposures during pregnancy									
Ambient air pollution	1·11 (1·03-1·19) [23]	Obs	Medium (9.5)	Residential green and blue space	Low (7)	Medium-Low	Yes	Country specific	Medium
Indoor air pollution	1·30 (1·06-1·59) [24]	Obs	Medium (9)	Cookstoves	Low (8)	Medium-Low	Yes	Country specific	Low
Intimate partner violence	1·89 (1·43-2·48) [25]	Obs	Medium (9.5)	Integrated cognitive behavioral intervention	Low (6.5)	Medium-Low	Yes	Country specific & regional	Medium
Tobacco smoking	1·27 (1·21-1·32) [26]	Obs	Medium (8)	Smoking cessation programs	Low (9.5)	Medium-Low	Yes	Regional	Low
Uterine, Placental and Cervical factors									
Short cervical length (<25mm)	6·19 (3·84-9·97) [27]	Obs	Low (4)	Pessary	Low (7.5)	Low	Yes	Global	Low
Pregnancy related morbidity									
Gestational diabetes	1·42 (1·15-1·77) [28]	Obs	Medium (8.5)	Insulin, diet and exercise, anti-diabetic medications	Medium (10.5)	Medium	No	Regional	Low
Pre-eclampsia	1·89 (1·73-2·06) [20] reduced to 1·40 (1·32-2·02)	Obs	Medium (10)	Calcium supplementation	Medium	Medium	Yes	Regional	Low
Maternal infection									
HIV	1·49 (1·43-1·55) [29]	Obs	Medium (10)	ARV	Low (9.5)	Medium-Low	No	Country specific	High
Malaria	1.56 (1.28-1.90) [1]	Obs	High (11)	IPTp	Low (LBW as surrogate outcome; 8)	Medium	Yes	Country specific	Medium
Syphilis	3·22 (3·15-3·23) [30]	Obs	Medium (9)	Penicillin	Medium (10)	Medium	Yes	Regional	Low

Chlamydia	1·26 (1·15-1·38)[31]	Obs	Medium (10)	Erythromycin	Low (5.5)	Medium-Low	No	Regional	Low
Asymptomatic bacteriuria	1·96 (1·45-2·77)[32]	Obs	Low (6)	Screen and treat	Low (8)	Low	No	Country specific	Medium
Periodontal Infection	1·61 (1.33-1·95)[33]	Obs	Medium (9)	Varying interventions	Low (9.5)	Medium	No	Country specific	Medium
Bacterial vaginosis	1·64 (1·36-2·33)[34]	Obs	Medium (8)	Screen and treat	Low (9.5)	Medium-Low	No	Country specific & regional	Medium
Group B Strep colonization	1.21 (0·99-1·48)[35]	Obs	Medium (9.5)	Penicillin	Low (9)	Medium-Low	No	Regional	Low

Table 2. Excluded Risk Factors

Risk Factor	Relative Risk or odds ratio (bounds)	RR source	Risk Quality	Intervention	Intervention quality	Overall quality	Agreement on effects	Reason for exclusion
**Pregnancy History**								
Previous preterm birth	2·60 (1·90-3·60)[36]	Obs	Low	NA	NA	Very low	NA	Not an independent risk factor
Previously induced abortion	1·33 (0·81-2·17)[37]	Obs	Low	NA	NA	Very low	NA	Evidence of no risk
**Maternal Nutritional Status**								
Low Vitamin A Intake	1·02 (0·99-1·05)[38]	RCT	High	NA	NA	High	NA	Evidence of no risk
Low Vitamin D Intake	1·52 (0·76-2·94)[39]	RCT	Medium	NA	NA	Medium	NA	Not an independent risk factor (Calcium overlap)

High BMI (>25 kg/m^2)	0·93 (0·85-1·01)[40]	Obs	Medium	NA	NA	Low	NA	Evidence of no risk
**Maternal Morbidity**								
Maternal depression	1·13 (1·06-1·21)[41]	Obs	Low	NA	NA	Very low	NA	Not an independent risk factor
**Environmental Exposure during Pregnancy**								
Physical exertion	1·32 (0·85-2·03)[42]	Obs	Low	NA	NA	Very low	NA	Insufficient evidence
**Uterine, Placental, and Cervical Factors**								
Fetal fibronectin	4·28 (2·29-8·04)[43]	Obs	Low	NA	NA	Very low	NA	Not an independent risk factor
Early vaginal bleeding	1·62 (1·29-2·02)[44]	Obs	Medium	NA	NA	Low	NA	Not an independent risk factor
Placental abruption	6·60 (5·40-7·90)[45]	Obs	Low	NA	NA	Very low	NA	Insufficient evidence
Placenta previa	6·04 (3·27-11·15)[46]	Obs	Low	NA	NA	Very low	NA	Insufficient evidence
Uterine abnormalities	OR/RR not reported	Obs	Low	NA	NA	Very low	NA	Insufficient evidence
Uterine fibroids	1·0 (0·9-1·2)[47]	Obs	Low	NA	NA	Very low	NA	Evidence of no risk
**Maternal Infections**								
Trichomonas vaginalis	1·30 (1·10-1·40)[48]	Obs	Low	NA	NA	Very low	NA	Insufficient evidence
Helminth infection	1·13 (0·56-2·33)[49]	RCT	Medium	NA	NA	Medium	NA	Evidence of no risk

Influenza infection	1.40 (0.9- 2.0)	Obs	Medium	NA	NA	Low	NA	Evidence of no risk
**Other**								
Genetic factors	OR/RR not reported	Obs	Low	NA	NA	Very low	NA	Insufficient evidence

Table 3. Weighted prevalence estimates by WHO region

	Global	AFRO	AMRO	EMRO	EURO	SEARO	WPRO
Short birth interval	6.4%	6.0%	5.8%	9.1%	7.5%	5.8%	9.0%
Young maternal age (< 18 years) & primiparity	6.7%	6.1%	6.5%	3.0%	1.8%	9.2%	3.6%
Maternal age 18-35 years & parity >3	35.4%	41.1%	27.0%	40.6%	31.1%	29.1%	27.6%

Older maternal age (>35 years) & parity >3	10.6%	13.5%	10.6%	11.7%	6.0%	7.3%	12.6%
Maternal Nutritional status							
Low Zinc Intake	21.9%	25.1%	18.6%	18.4%	9.6%	20.5%	22.1%
Maternal Anemia	38.2%	46.0%	37.0%	43.0%	31.5%	29.1%	34.0%
Low Calcium Intake	58.0%	73.7%	55.1%	34.3%	24.3%	48.2%	93.2%
Short height (<145 cm)	5.6%	2.7%	5.2%	5.4%	0.8%	8.9%	5.4%
Low BMI (<18·5 kg/m^2)	11.3%	12.1%	5.9%	13.8%	5.7%	10.0%	14.6%
Maternal Morbidity							
Chronic hypertension	17.7%	23.0%	16.8%	11.5%	14.6%	15.1%	11.4%
Fetal characteristics							
Twin pregnancy	1.5%	1.8%	1.3%	1.5%	3.0%	1.1%	1.1%
Fetal gender (male)	50.0%	50.0%	50.0%	50.0%	50.0%	50.0%	50.0%
Environmental exposures during pregnancy							
Ambient air pollution	50.9%	47.9%	38.2%	54.5%	46.8%	55.0%	37.0%
Indoor air pollution	62.1%	75.9%	47.1%	57.5%	9.6%	53.0%	58.1%
Intimate partner violence	6.9%	6.7%	7.9%	7.7%	4.2%	7.3%	3.5%
Tobacco smoking	1.5%	0.9%	3.9%	1.2%	6.6%	1.5%	4.2%
Uterine, Placental and Cervical factors							
Short cervical length (<25mm)	0.8%	0.8%	0.8%	0.8%	0.8%	0.8%	0.8%
Pregnancy related morbidity							
Gestational diabetes	9.8%	8.9%	9.5%	12.8%	5.8%	9.8%	11.7%

Pre-eclampsia	3.0%	3.9%	2.5%	1.4%	3.8%	2.4%	4.2%
Maternal infection							
HIV	1.4%	3.2%	0.9%	0.3%	0.1%	0.2%	0.1%
Malaria	6.3%	14.7%	3.9%	1.6%	0.0%	0.1%	0.8%
Syphilis	0.8%	1.4%	0.6%	0.5%	0.2%	0.4%	0.2%
Chlamydia	4.8%	3.9%	6.4%	3.3%	2.2%	6.0%	6.2%
Asymptomatic bacteriuria	0.1%	0.1%	0.1%	0.1%	0.3%	0.1%	0.1%
Periodontal Infection	7.9%	5.9%	10.8%	9.1%	6.4%	9.9%	1.6%
Bacterial vaginosis	28.7%	33.5%	26.1%	25.5%	22.8%	25.5%	24.2%
Group B Strep colonization	19.1%	22.2%	19.9%	16.1%	19.0%	17.4%	13.3%

Quality Assessment

Table 4a. Observational quality checklist

	Checklist		Score
**A**	**Method section**		
1	**Outcome definition: PTB**	<37 weeks gestational age, explicitly spontaneous PTB	1
		<37 weeks gestational age, either unspecified or not exclusively spontaneous PTB	0.5
		Other or Undefined	0
2	**Exposure/covariates definition**	Defined / consistent definition across all studies in meta	1
		Some defined while some not/ different definitions across studies in meta	0.5
		Not defined	0
3	**Timeline**	This paper was published in the last 5-10 years	1
		This paper was published more than 11 years ago	0
		All the studies included in the paper had data collected in the last 10-15 years	1
		Some of the studies included had data collected in the last 10-15 years	0.5
		Study included had data collected more than 16 years ago	0
4	**Country where the study was conducted**	Sufficient (4-5 studies conducted in LMICs)	1
		Some (<4 of studies conducted in LMICs)	0.5
		None in LMICs	0
5	**Number of data sets**	Systematic review and meta-analysis (=>5 datasets)	2
		Systematic review and meta-analysis (<5 datasets)	1
		Single studies (but consistent evidence from # of single studies)	0.5
		Only a single observational study	0
6	**Data items**	Large sample size in the research paper (>10,000)	1

		Small sample size in the research paper	0
**B**	**Result section**		
2	**Number of variables adjusted in the model**	Adjusted for relatively sufficient variables (#)	1
		Adjusted for relatively insufficient variables (#)	0.5
		Did not adjust for any variables	0
3	**Risk of bias**		
	**In individual or across studies**	Describe methods used for assessing risk of bias of individual studies (including specification of whether this was done at the study or outcome level), and how this information is to be used in any data synthesis.	1
	**Across studies**	Specify any assessment of risk of bias that may affect the cumulative evidence (e.g., publication bias, selective reporting within studies).	1
4	**Heterogeneity**	Low heterogeneity (0-30%)	2
		Medium heterogeneity (30-60%)	1
		High heterogeneity (>60%)	0.5
		No heterogeneity provided	0
5	**Magnitude of effect**	Statistically significant large magnitude (>=1.10)	1
		**Total**	**14**
	High Quality >10; Medium Quality 8-10; Low Quality <8		

Table 4b. Quality checklist for intervention

	Checklist		Score
**A**	**Method section**		
1	**Outcome definition: PTB**	<37 weeks gestational age, explicitly spontaneous PTB	1
		<37 weeks gestational age, either unspecified or not exclusively spontaneous PTB	0.5
		Other or Undefined	0
2	**Exposure/covariates definition**	Defined / consistent definition across all studies in meta	1
		Some defined while some not/ different definitions across studies in meta	0.5
		Not defined	0
3	**Timeline**	Published in the last 5-10 years	1

		This paper was published more than 11 years ago	0
		All the studies included in the paper had data collected in the last 10-15 years	1
		Some of the studies included had data collected in the last 10-15 years	0.5
		Study included had data collected more than 16 years ago	0
4	**Country where the study was conducted**	Sufficient (4-5 of studies conducted in LMICs)	1
		Some (<4 of studies conducted in LMICs)	0.5
		None in LMICs	0
5	**Number of data sets**	Systematic review and meta-analysis (=>5 datasets)	2
		Systematic review and meta-analysis (<5 datasets)	1
		Single studies (but consistent evidence from # of single studies)	0.5
		Only a single RCT/observational study	0
6	**Data items**	Large sample size in the research paper (>10,000)	1
		Small sample size in the research paper	0
7	**Study design**	Both RCT and Observational	2
		Only RCT	1
		Only Observational	0
**B**	**Result section**		
1	**Adjusted versus unadjusted estimates**	Adjusted RR/OR	1
		Some studies adjusted while some unadjusted	0.5
		Did not adjust for any variables	0
2	**Number of variables adjusted in the model**	Adjusted for relatively sufficient variables (#)	1
		Adjusted for relatively insufficient variables (#)	0.5
		Did not adjust for any variables	0
3	**Risk of bias**		
	**In individual or across studies**	Describe methods used for assessing risk of bias of individual studies (including specification of whether this was done at	1

		the study or outcome level), and how this information is to be used in any data synthesis.	
	**Across studies**	Specify any assessment of risk of bias that may affect the cumulative evidence (e.g., publication bias, selective reporting within studies).	1
4	**Heterogeneity**	Low heterogeneity (0-30%)	2
		Medium heterogeneity (30%-60%)	1
		High heterogeneity (>60%)	0.5
		No heterogeneity provided	0
5	**Magnitude of effect**	Large magnitude (>1.x)	1
		**Total**	**17**
	High quality: >13; Medium quality 10-13; Low quality <10		

Table 4c. Overall quality of risk estimates

**Observational**	**RCT/Intervention Trials**	**Overall Quality**
High	High	High
Medium	High	High
Low	High	High
High	Medium	Medium-High
Medium	Medium	Medium
Low	Medium	Medium-Low
High	Low	Medium
Medium	Low	Medium-Low
Low	Low	Low
High	None	Medium
Medium	None	Low
Low	None	Very Low
None	High	High
None	Medium	Medium
None	Low	Very low

2. Risk Estimate Sources and Quality

In this section of the supplementary materials we describe the data available to estimate the risk of spontaneous preterm birth given the presence of an assumed risk factor. For each of the factors reviewed we provide the best estimate and a brief description of the source of the estimated risk of spontaneous preterm birth given the presence of the factor as well as a grade of the quality of the evidence.

Pregnancy History

Maternal age and parity

Risk: Maternal age and parity are often cited in the literature as independent risks for preterm birth. However, it is very difficult to separate the risks entirely; younger women are more often primiparous and older women often have higher parity. In the analysis below, comprised of 14 cohort studies from multiple countries, there were zero studies that had a population prevalence greater than 5% for primiparous women older than 35 or women younger than 18 with a parity greater than three.[(14)] Due to the interconnectedness of the relationship, we will be considering these two risk factors together in the analysis.

The meta-analysis for maternal age and parity was completed using data from 14 prospective cohort studies in 9 different countries in Asia, Africa and South America. The adjusted ORs estimated in the analysis are adjusted for the following potential confounders: maternal education, ethnicity, land ownership, housing characteristics, income, antenatal care, height, weight, and MUAC.

Quality of risk estimate: Medium. The 14 prospective cohorts included in the analysis result in a large sample size and controlled for confounders in the analysis. Additionally, many of the sites included in the study have been established for many years and have experience measuring gestational age, which is important to avoid misclassification of preterm births. However, there was still some variation in the quality of gestational age measurement across the cohorts.

Young maternal age < 18 & Primiparity

Risk: The risk estimate for the association between primiparity, maternal age <18 years, and preterm birth is OR=1·52 (1·40-1·66), compared to women ages 18-35 with parity 1-2 and adjusted potential confounders. This is from a meta-analysis of 14 prospective cohort studies published in 2013 (N=90,168). The prevalence of primiparous women with a maternal age <18 years was 8·13%, which was used in the conversion of the odds ratio to a risk ratio equal to RR=1·46 (1·36-1·58).[(14)]

MODEL INCLUSION: RR=1·46 (1·36-1·58). Consistent evidence based on observation data, but no possibility for treatment during pregnancy and therefore no supporting evidence for an intervention effect

Maternal age 18-35 and primiparous

Risk: The risk of preterm birth among primiparous women between 18 and <35 compared to women ages 18-35 with parity 1-2 was insignificant (OR=1·09 [0·99-1·21]) with adjustment for potential confounders. This 95% confidence interval does not overlap with the confidence interval for the younger, primiparous women, demonstrating that younger maternal age is likely the stronger driver of the risk relationship than primiparity.[14]

MODEL INCLUSION: Not included in the model due to absence of risk for spontaneous preterm birth

Maternal age 18-35 and multiparous

Risk: This age group of women (18 to <35) who had a parity >3 had significantly increased odds of PTB (OR=1·20 [1·06-1·35]) compared to women of the same age with a parity of either 1 or 2. The prevalence of this age and parity category was equal to 13·24%, which was used to convert the OR to a RR= 1·17 (1·05-1·29).[14]

MODEL INCLUSION: RR=1·17 (1·05-1·29). Consistent evidence based on observation data, but no possibility for treatment during pregnancy and therefore no supporting evidence for an intervention effect

Older maternal age >35 & Parity >3

Risk: The risk estimate for the association between parity > 3, maternal age >35 years, and preterm birth is OR=1·43 (1·21-1·69), compared to women ages 18-35 with parity 1-2 and adjusted potential confounders. The prevalence of maternal age >35 years and parity >3 in this study was 6·31%, which was used in the conversion of the odds ratio to a risk ratio equal to RR=1·39 (1·19-1·62).[14]

MODEL INCLUSION: RR=1·39 (1·19-1·62). Consistent evidence based on observation data, but no possibility for treatment during pregnancy and therefore no supporting evidence for an intervention effect

Short birth interval

Risk: The risk estimate for the association between short birth interval (< 18 months) compared to recommended pregnancy intervals (36-60 months) is from a meta-analysis of 12 cohort studies (N=19,240) from 2013 that reported an odds ratio of OR= 1·58 (1·19-2·10), which was adjusted for income, maternal education, height, BMI, maternal age, and parity.[13] The paper references an assumption of a 10% prevalence overall, which is used in the conversion of the odds ratio to a risk ratio equal to RR=1·49 (1·17-1·89).

Quality of risk estimate: Medium. The twelve prospective cohorts included in the analysis result in a large sample size and control for confounders in the analysis. Additionally, many of the sites included in the study have been established for many years and have experience measuring

gestational age, which is important to avoid misclassification of preterm birth. Finally, the results from this analysis are similar to those of Conde-Agudelo, which included different studies (note: also a different exposure definition; birth vs. pregnancy interval, but still comparable).[50]

MODEL INCLUSION: RR=1·49 (1·17-1·89) Consistent observational evidence, but not amenable to intervention during pregnancy and therefore no supporting evidence for an intervention effect

Previous PTB preterm birth

Risk: The Preterm Birth Prediction Study did an analysis that specifically examined risk factors for PTB and found previous spontaneous birth to have a crude RR=2·7 (2·1-3·4) and an adjusted RR=2·6 (1·9-3·6).[36]

Quality of risk estimate: Very low. Although the analysis adjusted for potential confounders, it is a single study in a high-income setting and therefore there are concerns about generalizability. Additionally, this relationship is likely due to the presence of other risk factors and therefore is not considered an independent risk factor in the model.

MODEL INCLUSION: Not included in the model because not considered an independent risk factor

Previously induced Abortion

Risk: Most of the studies investigating the link between previously induced abortion and risk of preterm birth in subsequent pregnancies have been done in high-income countries. Recently, a systematic review and meta-analysis found that in the overall population, women with a history of uterine evacuation for induced termination of pregnancy (I-TOP) had a significantly higher risk of PTB (OR= 1·27 [1·12–1·44]).[51] Secondary analyses suggested that the risk is large due to surgical intervention, as opposed to medical abortions. This meta-analysis, while finding a significant OR, did have several limitations.[52] Primarily among the weakness of the findings is the lack of sufficient data on confounding factors in the studies. In addition, many of the studies were case-control and as with all studies of this type, recall biases are a concern.

A more recent study of the risk of preterm birth following an induced abortion from 1980 through 2008 in Scotland found a significant time trend.[53] Until the mid-90s there was a significant risk of preterm birth for women in the pregnancy following an induced abortion. The risk was strongest in the period 1980 -1983, and progressive became less through 1995. For the periods 2000-2003 and 2004 -2008 there was no longer a risk (OR= 0·98 and 1·02). This parallels the shift in abortion away from surgical abortion to medical abortion and increasing use of cervical pre-treatment prior to surgical abortion. The authors postulate that the previous links between IA and PTB are primarily driven by the use of surgical abortions in those earlier periods and note that many more studies find non-significant associations.

There are additional studies that have looked at the risk of preterm birth following a dilation and curettage (D & C) procedure. A recent systematic review meta-analysis of 21 studies found a

significant increase in the risk of preterm birth following a D & C procedure for a miscarriage or abortion.[54] In the 21 studies with over 1·8 million births it was found that women with a history of D & C had increased odds of a subsequent preterm birth (OR = 1·29 (1·17-1·42).While there are few data on the relationship between IA and PTB in LMIC, we did find two recent analyses, one in Guangdong China and a second in Hong Kong. Neither of these analyses found a significant risk of PTB given prior IA.

A prospective study examined the relationship between misoprostol induced abortions and preterm birth in a subsequent pregnancy. One of the exclusion criteria was induction of labor for any reason other than PPROM, so the cases are all spontaneous preterm births (with the exception of induced after PPROM). Controls (ratio 2:1) were spontaneous term births. Both the unadjusted and adjusted odds ratios demonstrated an insignificant risk of preterm birth after misoprostol induced abortion (OR=1·51 [0·95-2·39]; OR= 1·33 [0·81-2·17]).[37] This strengthens the argument that the relationship between previous induced abortion and PTB is primarily through cervical damage.

Quality of risk estimate: Very low. In many, but not all, of the observational studies there was controlling for possible confounders in the analysis. From these studies there is clear evidence that while surgical abortion can increase the risk of preterm birth probably through damage to the cervix, there is little evidence that medically induced abortions increase the risk of preterm births.

MODEL INCLUSION: Not included in the model due to evidence of no risk for spontaneous preterm birth

Maternal Nutritional Status

Low Vitamin A Intake

Risk: The risk for Vitamin A deficiency is the inverse of the RR determined in the 2015 Cochrane review of Vitamin A supplementation vs. placebo. This review included five studies and demonstrated a treatment effect of RR=0·98 (0·94-1·01), which when the inverse is taken to estimate risk of Vitamin A deficiency is equal to RR=1·02 (0·99-1·05).[38]

Quality of risk estimate: High. The sample size for the five combined trials is very large (over 48,000 participants) and the individual trials are free from any potential biases.

MODEL INCLUSION: Not included in the model due to evidence of no risk for preterm birth

Low Zinc Intake

Risk: The 2021 Cochran review of Zinc supplementation vs. with or without placebo included 21 trials with a total number of participants equal to 9,851. The treatment effect was equal to RR=0.87 (0.74 – 1.03).[15] The certainty of the evidence was found to be low; the evidence was downgraded because of study design limitations and impreciseness. The analysis did not distinguish between populations of low zinc and nutrition and those of normal zinc and nutrition,

which is different than the previously published 2015 Cochrane. The inverse of the treatment effect is equal to RR=1.15 (0.97 – 1.35), which will be used in the extended model 2.

Quality of risk estimate: Medium. Although the meta-analysis had the large sample size and trials in both high and low-and-middle income countries, the Cochrane review deemed the quality to be “low”. This results in the risk estimate for zinc supplementation to be downgraded from high to medium

Other evidence of risk: The risk of preterm births with zinc deficiency is calculated to be the inverse of the risk reduction determined in the 2015 Cochrane review of Zinc supplementation vs. placebo among women with “low zinc or nutrition”. The review included 16 studies and demonstrated a treatment effect of RR= 0·87 (0·77- 0·98), which when the inverse is taken to estimate the risk of low zinc intake is equal to RR= 1·15 (1·02-1·30).(55) This estimate applies to all women in an area where there is some zinc deficiency, not strictly women who have been determined to be zinc deficient. This is reflected in the prevalence estimate.

Other quality of risk estimate: High. The Cochrane review deemed the quality of the 16 studies included in the review of “moderate” quality due to design limitations. Studies were conducted in both high and low-and-middle income countries.

MODEL INCLUSION: Extended model 2. RR=1.15 (0.97 – 1.35) Borderline insignificant effect in most recent Cochrane.

Maternal Anemia

Risk: There was one recent systematic review and meta-analysis on the impact of maternal anemia on birth outcomes in low- and middle-income countries.(16) This meta-analysis included 13 cohort studies that measured gestational age. The study found a significant risk relationship (RR =1·63 [1·33-2·01]) between maternal anemia and preterm birth. An older meta-analysis of cohort studies reported a pooled RR=1·28 (1·11–1·48).(56)

Quality of risk estimate: Medium-High. The Rahman et al. study included data from multiple LMIC’s and included appropriate control measures in the selection and analysis of the included studies. Both meta-analyses computed an adjusted RR for risk and ran various stratified analyses. A weakness of the Rahman et al. analysis was that almost all of the data on preterm births came from countries in Asia.

Intervention evidence: A systematic review and meta-analysis of iron supplementation trials summarized their effects on maternal anemia, iron supplementation and birth outcomes.(56) In this meta-analysis there were 48 randomized trials (17,793 women) and 44 cohort studies (1,851,682 women). Iron use increased maternal mean hemoglobin concentration by 4·59 (95% confidence interval 3·72 -5·46) g/L compared with controls and significantly reduced the risk of anemia (RR= 0·50 [0·42-0·59]), iron deficiency (RR=0·59 [0·46-0·79]), iron deficiency anemia (RR=0·40 [0·26-0·60]), and low birth weight (RR=0·81 [0·71-0·93]). The effect of iron on preterm birth was not significant (RR= 0·84 [0·68-1·03]).

A 2015 Cochrane review compared any daily iron regimen (with or without other vitamins and minerals) vs. daily regimen (with same vitamins and minerals). The review included 13 studies and did not find a treatment effect on preterm birth (RR=0·93 [0·84-1·03]),[(57)] which when the inverse is taken to estimate risk of iron deficiency is equal to RR=1·07 (0·97-1·19). There were no studies where the participants were anemic at the start of supplementation. The association of iron deficiency and preterm birth is unclear in the published literature.

MODEL INCLUSION: RR= 1·63 (1·33-2·01). Consistent evidence based on observational data. However, anemia may be an indicator of other conditions and risk factors that were considered (iron deficiency, helminth infection, malaria, etc.), but were found to not have a treatment effect. For iron deficiency anemia and helminth infection, the treatment meta-analyses demonstrated there was no significant effect of treatment on preterm birth. For malaria there are very few studies with preterm birth as the outcome. Therefore, we are including maternal anemia, but acknowledging the limitation that it is unclear what causes of anemia are responsible for the relationship with preterm birth.

Low calcium intake

Risk from RCT: A systematic review of high-dose calcium supplementation (>1g/day) during pregnancy included 13 trials. The meta-analyses found a significant decrease in blood pressure ((12 trials, 15,470 women: RR= 0·65 [0·53-0·81]), and pre-eclampsia (13 trials, 15,730 women: RR=0·45 [0·31- 0·65]).[(17)] In addition, the impact of calcium supplementation on preeclampsia risk was greater for women with low calcium diets (eight trials, 10,678 women: RR=0·36 [0·20-0·65]). This same review also found a significant reduction in preterm births for women receiving calcium supplementation (11 trials, 15,275 women: RR=0·76 [0·60-0·97]). However, when the population is limited to a "low calcium diet", the reduction in preterm birth births is smaller and no longer statistically significant (7 trials, 10,242 women: RR= 0.81 [0.62, 1.02]). This estimate more accurately reflects the populations in this analysis and therefore the risk factor "low calcium intake" will only be used in the extended model. The inverse of the low calcium diet risk estimate will be used in Model 2, which is equal to RR=1·23 (0.98-1·56). This estimate applies to all women who live in areas with calcium deficiency but is not exclusive to women who have been determined to be calcium deficient (see below for those risk estimates). This is reflected in the prevalence estimate.

The authors published another paper that was a subset of an older meta-analysis, specifically in LMICs. They demonstrated a reduced risk of pre-eclampsia (10 trials, >11,000 women, RR=0·41 [0·24-0·69]). Using the CHERG adaption of the GRADE guidelines, the data quality for the association between Ca supplementation and pre-eclampsia was graded "high". There was also a significant reduction in preterm birth (5 trials, 9,919 women, RR=0·88 [0·78-0·99]).[(58)]

Quality of risk estimate: Medium. The randomized controlled treatment trials have shown that high-dose calcium supplementation reduces the risk of pre-term births as well as intermediate outcomes (pre-eclampsia). However, there are concerns with the heterogeneity of the patient populations and sizes of the trials included in the analysis.

MODEL INCLUSION: Extended Model 2. RR=1·32 (0.98-1·56). Borderline insignificant evidence from randomized controlled treatment trials.

Vitamin D deficiency

Risk: Initially, the 2016 Cochrane review was included in this analysis, but during the process the 2019 review was published. The most recent estimates report no impact of Vitamin D supplementation on preterm birth (RR=0·66 [0·34-1·30], 7 trials, low quality).(39) The inverse of this estimate is equal to RR= 1·52 (0·76- 2·94). This is compared to the 2016 results (below), which included three trials (two of which are the same as the 2019 trials, one of which was dropped and four of which are new in the 2019 analysis). The increased risk of preterm birth amongst women who received both calcium and vitamin D is included in both analyses. The 2019 analysis reports a RR=1·52 (1·01-2·28) (5 trials, low quality). Of the 4 new trials in the 2019 analysis, 2 are in LMICs and 2 are in HICs. Those trials in HICs may attenuate the estimate, as it is less likely women in these settings are vitamin D deficient and therefore will not benefit from vitamin D supplementation.

In the 2016 Cochrane Review of the impact of vitamin D supplementation during pregnancy, the data from 15 trials were analyzed.(59) Data from two trials involving 219 women suggest that women who received vitamin D supplements may have a lower risk of preeclampsia than those receiving no intervention or placebo (RR= 0·52 [0·25-1·05], low quality). With respect to infant outcomes, data from three trials involving 477 women suggest that vitamin D supplementation during pregnancy reduces the risk of preterm birth compared to no intervention or placebo (RR=0.36 [0·14 to 0·93], moderate quality). The inverse of this treatment effect is equal to RR=2·77 (1·07-7·14).

The review also includes trials where women received both calcium supplementation and vitamin D. Women who received vitamin D with calcium had a lower risk of pre-eclampsia than those not receiving any intervention (RR= 0·51 [0·32- 0·80]; three trials; 1114 women, moderate quality), but also an increased risk of preterm birth (RR= 1·57 [1·02- 2·43], three studies, 798 women, moderate quality).

These results suggest that either Vitamin D supplementation alone or calcium supplementation alone can reduce the risk of pre-eclampsia and preterm birth. However, combining the two increases the risk of preterm birth. For our model we have assumed that calcium supplementation and vitamin D supplementation operate primarily through the same risk, related to low levels of calcium.

Quality of risk estimate: Medium. Although both meta-analyses are pooled estimates from multiple RCTs, the studies included are graded as “low” quality by Cochrane because of concerns with selection bias.

MODEL INCLUSION: Not included in any model because assumed that Vitamin D effects operate through calcium deficiency, which is already included.

Short height (<145 cm)

Risk: The risk estimate for the association between preterm birth (regardless of SGA status) and short maternal height (<145 cm) is equal to an adjusted RR= 1·42 (1·10-1·83), which was adjusted for parity, maternal age, maternal education, antenatal care visits, and maternal urinary tract infection. This estimate is from a meta-analysis of 12 prospective cohort studies (N=36,803) in LMICs published in 2015.[18]

Quality of risk estimate: Medium. The twelve prospective cohorts included in the analysis result in a large sample size and control for confounders in the analysis. Additionally, many (but not all) of the sites included in the study have been established for many years and have experience measuring gestational age, which is important to avoid misclassification of preterm births. However, there was still some variation in the quality of gestational age measurement across the cohorts.

MODEL INCLUSION: RR=1·42 (1·10-1·83) Consistent evidence based on observational data, but not amenable to intervention during pregnancy and therefore no supporting evidence for an intervention effect

Low Pre-pregnancy BMI

Risk: The risk of spontaneous preterm birth associated with low BMI (<18.5kg/m^2) is from a meta-analysis published in 2010 and found an RR=1·32 (1·10-1·57) which was a pooled estimate from 8 studies that controlled for potential confounders, though the exact confounders differed between the studies.[19]

Quality of risk estimate: Medium. In the analysis there are both retrospective and prospective cohort studies included, which together had a large sample size and the analysis controlled for potential confounders. However, there were fewer studies in low- and middle-income countries and when disaggregated into developed and developing countries, the effect for developing countries was insignificant. This limits the generalizability of the results of this meta-analysis to the countries included in the model.

Intervention evidence: Interventions to address low BMI during pregnancy have been shown to have mixed results with lowering preterm birth. Balanced energy protein supplementation has been shown to have no impact on preterm birth (RR=0·96 [0·80-1·15]).[60] While nutrition education and counseling has been shown to decrease preterm birth by up to 19%,[61] quantity of foods consumed is only one aspect of the counseling curriculum. Therefore, the benefits may be from one of the other aspects (diversity of food, encouraged use of micronutrient supplements, etc.) of the counseling rather than the increased caloric intake.

MODEL INCLUSION: RR=1·32 (1·10-1·57). Consistent evidence based on observational data, but no consistent evidence supporting an intervention effect

High pre-pregnancy BMI

Risk: For spontaneous preterm births, this meta-analysis included 15 studies (total sample size not listed) and found no effect of BMI greater than 25, RR=0·94 (0·80-1·10). However, this number is from crude data, not adjusted. The analysis also examined the risk of preterm birth for overweight (BMI 25 - <30, 4 studies), obese (BMI 30-<35, 2 studies), and very obese (BMI >35, 2 studies) women and found adjusted RRs equal to 0·94 (0·80 to 1·10), 1·04 (0·92 to 1·17), and 0·95 (0·67 to 1·33), respectively. Additionally, the authors disaggregated data by developing and developed countries and found no differences between the two categories (RR=0·83 [0·61-1·12] vs RR=1·09 [0·87-1·36]), though this analysis does not separate spontaneous and iatrogenic births and it includes both adjusted and unadjusted data.[40]

Quality of risk estimate: Low. Although the summative risk estimate across all 15 studies was from crude data, the analysis by obesity class was adjusted for and the results from the two analyses are in agreement. Additionally, the analysis examined the relationship between high BMI and preterm birth in developing and developed countries separately, which strengthens the generalizability of the analysis to countries included in this model.

MODEL INCLUSION: Not included in the model due to evidence of no risk for spontaneous preterm birth

Maternal Morbidity

Chronic hypertension

Risk: The risk of chronic hypertension on spontaneous preterm birth is an adjusted OR=1·65 (1·33-2·05) which was estimated using a multilevel multivariate logistic regression model. The multilevel analysis was structured on three-levels (individual, health facility, country) with random intercepts, and adjusted for maternal age, marital status, education, parity, and previous caesarean section. This measure is from a secondary analysis of 16,474 singleton preterm births collected by the WHO multi-country survey on maternal and newborn health and published in 2014. This cross-sectional survey was conducted in health facilities across 29 countries. Chronic hypertension status was obtained via medical record review. The prevalence of hypertension in this study population was 11%, and converting this odds ratio to a risk ratio results in an estimate equal to RR=1·54 (1·28-1·93).[20]

Quality of risk estimate: Medium-High. The large sample size, specific measurement of spontaneous preterm birth, hierarchical model use and LMIC sites earn this risk factor a quality grade of “high”

Intervention evidence: The Cochrane review of 15 randomized controlled trials comparing any antihypertensive drug during pregnancy compared to no drug/placebo demonstrated no effect on preterm birth (RR=0·96 [0.83-1.12]).[62] The 15 RCTs included in the meta-analysis were of “moderate quality”, with the major limitation being that they did not “downgrade for indirectness”, or, that the studies examined a wide range of drugs, not a specific drug. With 15 trials completed, it is not an absence of evidence (as is the case with some risk factors), but rather evidence of absence for the impact of anti-hypertensive drugs on preterm birth. It should be

noted that there are interventions for chronic hypertension that are not specific for treatment during pregnancy (diet, exercise, etc), but these are outside the scope of this analysis.

MODEL INCLUSION: RR=1·54 (1·28-1·93). Consistent observational evidence, but no evidence supporting an intervention effect.

Maternal Depression

Risk: The 2016 Lancet series on maternal depression in low and middle incomes countries cites 19-25% of women experiencing antenatal depression in LMICs, compared to 7 to 15% in high income countries. Antenatal depression can be a result of many factors, including intimate partner violence or unintended pregnancy. Additionally, it can lead to other risk factors for preterm birth like poor nutrition status, lack of antenatal care, or substance use during pregnancy. A total of 51 articles was included in this meta-analysis, where there were different methods used to detect/diagnose antenatal depression across the studies. This source reported a pooled prevalence of antenatal depression equal to 25·3%.[(63)]

The series paper cites one article examining antenatal maternal depression and spontaneous preterm birth. The case-control study conducted in a hospital in Peru used two different scales to examine this relationship. There is no matching process described in the methods, but multiple maternal characteristics were adjusted for in the analysis. Both scales for depression demonstrated an increased risk of spontaneous PTB with increased depression severity measured by the PHQ9: mild OR=2·22 (1·64–3·00) and moderate-severe OR=3·67 (2·09–6·46). Measured by the DASS-2:1 mild 1.00 (ref), moderate OR= 3·82 (1·90–7·66), and moderate- severe OR= 2·90 (1·66–5·04).[(64)]

Another meta-analysis of 20 studies, the majority of which were conducted in the United States, found a pooled RR=1·13 (1·06-1·21). The effect was larger in developing countries (only two studies), RR=2·22 (1·03-4·79).[(41)]

Quality of risk estimate: Very low. Some of the studies in the meta-analyses and the individual study controlled for potential confounders in the analysis. Additionally, there is research done in LMIC settings. However, as the Lancet series mentions, maternal depression can lead to other preterm birth risk factors and therefore we will not be considering it as an independent risk factor in the model.

MODEL INCLUSION: Not included in the model due to concerns with causal pathway, as outlined in the Lancet paper

Fetal characteristics

Twin pregnancy

Risk: We ran a systematic review of articles looking for relative risk of preterm births given twins. A meta-analysis of LMIC included data from 23 low and middle income countries, which resulted in a sample size of 276,187 singleton births and 6,476 twin births (twin births

representing 1·2% of births in the data set). Preterm birth was not a primary outcome of the analysis and the authors did not differentiate between spontaneous and provider initiated preterm birth. They reported that 35% of the twin pregnancies were born prior to 37-weeks gestation, compared to 9.6% of single pregnancies (Chi-squared p-value <0.001).(21) This incidence estimate is not adjusted, but the authors note that this pattern of increased risk of preterm birth "persisted regardless of region (or) mode of delivery…". Using these estimates, we calculated an unadjusted RR= 3·65. Other published individual studies varied widely in terms of RR, with the lowest being a community-based trial in Bangladesh with an RR of 1.61,(65) up to a hospital-based retrospective analysis in Nigeria that found an unadjusted RR of 6·5.(66)

Note: It is commonly cited that more than 50% of twin pregnancies result in preterm delivery. Chauhaun et al. determined that one third of preterm births in the United States are spontaneous. Therefore, 15% of twin pregnancies in the US should be spontaneous preterm deliveries.(67) In a study in Korea, they reported 8% of twin pregnancies ending in spontaneous preterm birth amongst women of normal weight.(68) The discrepancies between these two numbers are concerning, despite being in different countries.

Quality of risk estimate: Low. Although it is accepted that twin pregnancies are often preterm deliveries, the exact risk of spontaneous preterm birth and twin pregnancies is not well quantified. The risk relationship is from multiple LMIC settings, however there was no adjustment for potential confounders in the analysis.

MODEL INCLUSION: RR=3·65. Consistent evidence based on observation data, but no possible treatment and therefore no supporting evidence for an intervention effect

Fetal sex

Risk: Although the mechanism behind the relationship is unclear, a higher proportion of male babies are born preterm than female babies. This is true in both singleton and twin pregnancies. In fact, male-male twin pregnancies have higher rates of PTB than mixed gender twin pregnancies and female-female twin pregnancies.(69)

One meta-analysis was found investigating fetal sex and risk of preterm birth.(22) This meta-analysis measured the association between fetal sex and preterm birth in four original datasets, and 20 populations extracted from published birthweight references. The original samples were also analyzed by mode of onset. In total, data came from 15 European countries, the US, Canada, and Australia. Their analysis found that males had a higher risk of preterm births (an overall OR =1·12 [1·09–1·14]). This was converted to an RR=1·06 (1·04-1·07) using a prevalence estimate of 50%. Of the 24 populations, 20 out of 24 had OR between 1·09 and 1·15. For the two studies that had covariates reported the adjusted OR = 1·10 and 1·28.

There were two other large analyses of national birth registries. One in Australia analyzed 30 years of data on birth, the second in the Netherlands analyzed 12 years of birth registry data.(70, 71) The Australian study found a significant higher rate of spontaneous preterm births in males (RR=1·12 [1·04–1·20]). The Netherlands analysis also found greater preterm rates for males (OR = 1·80 [1·70-1·90]).

We found only one study from a low- and middle-income country. A study in China that primarily focused on exposure to air pollution found that male fetuses were at higher risk of preterm birth due to exposure to pollutants. Their study also showed that male fetuses had significantly higher rates of preterm birth than did females (M = 8·7%; F = 7·8%).[72] This ratio fits closely with those found in the studies in high-income countries.

Quality of risk estimate: Low. Very few of the studies outlined above adjusted for potential confounders in the analysis. Additionally, there is a lack of data of studies in a LMIC setting.

MODEL INCLUSION: RR=1·06 (1·04-1·07). Consistent observational evidence, but no possible intervention and therefore no evidence for an intervention effect

Environmental Exposures during Pregnancy

Intimate partner violence during pregnancy

Risk: The authors reported an adjusted OR=1·89 (1·43-2·48), from a pooled analysis of 13 studies. The prevalence across all the studies is very low, ~0.5%, so the conversion to the RR is identical to the OR (RR=1.89, 1.43-2.48). When limited to studies conducted in LMICs (9 studies), the unadjusted OR=2·75 (2·00-3·78). The authors also report unadjusted odds ratios for physical and emotional abuse separately, which have ORs= 1·71 (1·35-2·17) and 1·39 (1·13-1·71), respectively. There were no studies that reported estimates for PTB risk due to sexual violence. The combined violence types had the highest risk, an unadjusted OR=2·33 (1·88-2·88).[25]

Supporting risk: A meta-analysis of five studies reported adjusted odds ratios for a pooled odds ratio equal to OR=1·42 (1·21-1·63) for abuse (Physical, psychological, sexual, emotional) during pregnancy and preterm birth. The authors do not distinguish between spontaneous and provider initiated preterm birth. The studies were all conducted in high income countries (USA, Canada, Norway, Portugal). The five studies are three cross-sectional studies, one prospective cohort and one retrospective cohort. In the introduction of this paper, the authors describe that the maternal stress response to abuse is likely the cause of preterm birth (production of prostaglandin). This paper includes a good conceptual model for the pathways from the different forms of abuse to preterm birth.[73]

LMIC estimate: A prospective cohort study conducted (N=1112) in Tanzania compared women who experience intimate partner violence (IPV) during pregnancy to those who did not. Women who experienced physical abuse had an adjusted OR=2·90 (1·30-6·50). The relationships for emotional and sexual abuse were not statistically significant. 1/3 of women experienced IPV during pregnancy-- 22·3% emotional abuse, 15·4% sexual abuse and 6·3% physical abuse.[74]

Quality of risk estimate: Medium-Low. The studies included in meta-analyses for abuse during pregnancy and the single study in Tanzania all controlled for potential confounders in the analysis. Although the meta-analysis studies all occurred in high income countries, the results are in agreement with the study conducted in Tanzania.

Intervention evidence: An RCT examining an "integrated cognitive behavioral" intervention during antenatal care in women experiencing IPV during pregnancy. There was a significant reduction in episodes of IPV and incidence in very-preterm birth (<33 weeks gestation). There was a reduction in preterm birth (<37 weeks) between the intervention (13·0%) and comparison (19·7%) groups, but this reduction was statistically insignificant.[75] There was a significant increase in mean gestational age when comparing the two groups. There are few studies examining interventions for IPV during pregnancy that examine preterm birth as an outcome,

MODEL INCLUSION: RR=1·89 (1·43-2·48). Consistent observational evidence, some supporting treatment evidence (lack of available studies).

Physical exertion

The Lancet series on preterm birth states that the relationship between preterm birth and level physical activity/ type of work is inconsistent across the published observational studies. They cite four articles, three of which are in Europe and the US and one in Guatemala. The authors cite difficulty with accounting for confounding factors in these studies, but that "working long hours and undertaking hard physical labor under stressful conditions are probably associated with an increase in preterm birth". The studies conducted in the US and Europe demonstrated no increased risk.[4, 76, 77] Another study in the Netherlands also found no relationship between PTB and standing, lifting or number of hours worked per week.[42] For example, women who worked >40 hours per week saw an increased risk of preterm birth (OR=1·32 [0·85-2·03]), but it was statistically insignificant. However, the generalizability of these studies to our analysis is questionable.

The study in Guatemala demonstrated an increased risk in SGA/preterm birth in women with three or more children and no help compared to women with family or hired help, the assumption being that women without help have increased work. As physical demands of work increased (measured by an activity score), frequency of SGA/preterm also increased.[78]

A study in the Philippines found that among women in three different work strata, there were no consistent differences in preterm birth incidence. Of women who worked at home, those who stood longer had infants born at significantly lower gestational age. Additionally, women who were "economically inactive" and experienced higher levels of physical stress (spending time doing an activity at >0.6 kcal/kg/min) had infants of lower gestational age.[79] However, these are the only two statistically significant relationships out of the analysis.

Quality of risk estimate: Very low. The authors of the Lancet series acknowledge the lack of controlling in the analysis of the currently published studies. Furthermore, there are concerns with the generalizability of studies conducted in high-income settings, as the "work" is likely different than what is considered work in LMIC settings. The Guatemala study examines intensity of household responsibilities and controls for potential confounders (maternal height and age, household income, birthweight of previous child) in their analysis. There could also be reverse causality in this relation because women who are ill in pregnancy may not work (or do

physical activity) and the illness rather than the work or physical activity may be the cause of PTB.

MODEL INCLUSION: Not included in the model due to unavailability of data and inconsistent results from data that is available

Tobacco smoking

Risk: The most recent meta-analysis of 20 prospective studies (total sample size not stated) was published in 2000 and reports an association between smoking during pregnancy and preterm birth of a magnitude OR=1·27 (1·21-1·33), which was calculated using a Mantel-Haenszel fixed effects model. In the methods, the authors state that in their meta-analysis, "adjusted odds ratios were used whenever possible…most studies adjusted for maternal age, race, gravidity, parity, income, and other sociodemographic factors." The global prevalence of tobacco smoking in pregnancy is 1·3%, which was used to convert the odds ratio to an estimated RR=1·27 (1·21-1·32).[26]

Quality of risk estimate: Medium. Some, but not all of the studies included in the analysis controlled for potential confounders. Additionally, there were only developed countries included in the meta-analysis, which lowers generalizability to countries included in the model.

Intervention evidence: A 2009 Cochrane review of 14 trials reports that smoking cessation interventions resulted in a significant decrease in preterm birth (RR=0·86 [0·74-0·98]).[80] The majority of the interventions in this review were health education/behavioral change interventions, but there were also interventions biomedical interventions, such as nicotine replacement therapy.

A 2017 Cochrane review examined 19 studies on exclusively psychosocial interventions that demonstrated an insignificant decrease in preterm birth (RR=0·93 [0·77-1·11]).[81] Despite there being more trials included in this analysis, the total sample size is smaller than the 2009 Cochrane meta-analysis.

MODEL INCLUSION: RR=1·27 (1·21-1·32). Consistent evidence based on observational data, moderate evidence supporting intervention effect.

Air Pollution (ambient air particulate matter and indoor air pollution)

Ambient air pollution

Risk: The risk estimate is from a meta-analysis comprised of 13 studies, the majority of which took place in the United States. The pooled odds ratio for a 10 μg/m^3 increase in overall $PM_{2.5}$ exposure during pregnancy was stated as OR=1·13 (1·03-1·25).[23] The study used this 10 μg/m^3 increase to standardize the exposure levels across the different studies for the meta-analysis. The individual studies adjusted for potential confounders in their analysis, though the number of confounders differed between the studies. The vast majority of the studies were conducted in the

USA, so the prevalence estimate from the GBD 2015 of $PM_{2.5}$ exposure (17·5%) was used to convert the OR to an RR=1·11 (1·03-1·19).[82]

Quality of risk estimate: Medium-Low. All the studies included adjusted for potential confounders, which would typically earn this meta-analysis a "medium" grade. However, only one of the included studies was conducted in LMICs and this found no significant effect on preterm birth. Therefore, due to potential lack of generalizability, the quality of the risk estimate is medium-low.

Further explanation: The 2015 meta-analysis of 13 studies found a statistically significant relationship when results were pooled across the studies and standardized to represent the odds of preterm birth per $10\mu g/m^3$ increase in $PM_{2.5}$ exposure (OR=1·13 [1·03-1·24]).[23] Note that this is for outdoor, not indoor, air pollution. The majority of the studies estimated $PM_{2.5}$ concentrations by using the closest monitoring station to the subject's home. All studies included in this meta-analysis adjusted for potential confounders (though those included differed between studies) and scored medium to high on the Newcastle-Ottawa scale. However, the majority of the studies took place in the US and all but one study took place in a high development index (HDI) country. When disaggregated, the pooled OR from non-US countries is equal to 0·98 (0·95-1·01).

Another meta-analysis in HDI settings found an attenuated relationship between $PM_{2.5}$ and preterm birth, reporting an OR=1·03 (1·01-1·05) per IQR increase in $PM_{2.5}$ exposure and an OR= 1·02 (0·93-1·12) per $10\mu g/m^3$ increase in $PM_{2.5}$ exposure.[83] None of the above analyses separate spontaneous from provider initiated preterm births. Given the settings of the majority of the included studies, this means the relationship is likely overestimated.

The study that took place in a non-HDI setting is from an analysis using the WHO Global Survey on Maternal and Perinatal Health in 22 countries in sub Saharan Africa, Asia, and Latin America. The dataset contains only singleton, spontaneous births and controlled for potential confounders in the analysis. These data are collected at a facility level, so may not be entirely representative, but more so than the HDI settings above. This study measured $PM_{2.5}$ concentrations for each health facility through use of geocoding and construction of 50 km radii. The $PM_{2.5}$ concentrations were then assigned for that specific health facility radius. This analysis found no relationship between $PM_{2.5}$ exposure and preterm birth for the overall analysis. There were four exposure groups (quartiles of $PM_{2.5}$ exposure) and the reference group was a $PM_{2.5}$ concentration less than 6·35 (WHO cut off is 10). The other three groups (6·35 to <12·32, 12·32 to <22·2, >22·2) compared to the reference group for odds of spontaneous preterm birth had ORs equal to 1·08 (0·95-1·22), 1·05 (0·90-1·23), and 0·96 (0·79-1·17), respectively.[84] Furthermore, they did sub-analyses for China and India because these two countries had a wide range of $PM_{2.5}$ concentrations, which included the highest concentrations of all 22 countries. Here, China overall had a significant relationship (OR=1·11 [1·04-1·17]), but in the quartiles only the highest quartile compared to the lowest had an estimated impact on preterm birth (ref: <12·5, 12·5 to < 17·7 OR=0·77[0·45-1·13], 17·7 to <36·5 OR=0·97 [0·70-1·34], >36·5 OR=2·54 [1·42-4·55]). The relationship in India remained statistically insignificant, in fact an inverse relationship took place (overall OR=0·96 [0·91-1·03], ref: <18·8, 18·8 to < 35·3OR=1·08 [0·87-1·34], 35·3 to <70·3 OR=0·92 [0·72-1·19], >70·3 OR= 0·76 [0·49-1·17]). The lack of a consistent dose-

response relationship does not support the case for ambient air pollution as an independent risk factor for preterm birth.

The results from the observational studies examining the relationship between ambient air pollution and preterm birth are inconsistent. Furthermore, the results from the WHO survey, which are most generalizable to the countries in our analysis (overlap of countries included) and is limited to spontaneous preterm birth, shows no relationship. Observational studies are limited by potential biases, such as residual or unmeasured confounders and non-random selection of subjects. Ideally, a randomized controlled trial would be conducted to eliminate or limit these biases and produce a causal risk estimate. This type of trial is much more feasibly conducted for indoor/household air pollution.

Intervention evidence: A meta-analysis of 11 studies examining the effects of residential greenness on preterm birth reported no impact (Pooled OR: 0.99 [0.970 – 1.02], I^2=53.4%).[85] The studies were from high-income countries and while all included studies adjusted for potential confounders, the confounders included varied across studies.

Indoor air pollution

There is one meta-analysis of observational studies examining the relationship between household air pollution and preterm birth. The exposure for these studies is defined as solid fuel being the primary source of fuel for cooking and heating in the household. The meta-analysis included three trials conducted in India (N=11,728), Peru (N=190) and Guatemala (N=1,717). The meta-analysis reported a summary effect equal to 1·30 (1·06-1·59).[24] There was adjustment for confounding in the studies, but the variables differed across the three studies. These studies did not measure PM concentrations; the exposure variable was report of type of fuel use. As the majority of populations in LMICs use biomass as fuel, the real exposure of interest is the PM concentrations. Not having these concentrations available is a major limitation of these analyses.

There was only one RCT with published results conducted in LMICs that have had preterm birth as a measured outcome.[86, 87] This trial in Nigeria demonstrated a reduction in preterm birth of 40%. However, they did not see an improvement in indoor air quality as measured by $PM_{2.5}$ concentration between the intervention (ethanol stoves) and control (firewood) arms, so it is difficult to know if this reduction was due to the stoves or a separate, unmeasured confounder. Another randomized trial was conducted in rural Nepal (Katz J. et al., results forthcoming). The trial had two phases, one a cluster-randomized stepped wedge design that compared biomass stoves to those with a chimney for ventilation. The second phase compared the chimney vented biomass stove to a gas stove. In phases 1 & 2, $PM_{2.5}$ concentrations were reduced by ~32% and ~50%, respectively. Despite these large reductions, the results demonstrated that reducing indoor air pollution had no impact on preterm birth. Although, it is important to note that their reduced levels the $PM_{2.5}$ concentrations still far exceeded the limit set by the WHO. The results of this trial are not necessarily generalizable to outdoor ambient air pollution risk (dose, density, and composition of particles could be different).

Quality of risk estimate: Medium-Low. While the studies controlled for confounders, there are concerns about the exposure definition and relationship with PTB.

MODEL INCLUSION: Ambient air pollution: RR=1.11 (1.03-1.19). Indoor air pollution: RR=1.30 (1.06-1.59). For ambient, there is inconsistent observational evidence across different studies depending on inclusion and no evidence of an intervention impact,. For indoor air pollution there is a lack of intervention data that is likely attributable to the inability of the studies to reduce the $PM_{2.5}$ levels below the limit set by the WHO.

Uterine, Placental, and Cervical Factors

Short cervical length (<25mm) / Cervical insufficiency

Risk: The best estimate of risk of spontaneous preterm birth due to cervical insufficiency comes from a multi-site study in the US. In this study they found a significant relationship between cervix length at 24 weeks and risk of preterm births. The women with a cervical length in the 10th percentile (< 26mm) were compared to women with a cervical length above the 75th percentile. For reference, the 75th percentile was a cervical length of 40mm. This population was also much poorer and primarily African American as compared to the general population The estimated risk is stated as RR=6·19 (3·84-9·97). The study did not control for any potential confounders in their analysis.(27)

Quality of risk estimate: Low. The risk estimate comes from a single prospective study (albeit a multiple site study) in a high income country, which limits generalizability. Additionally, there was no adjusting for confounders in the analysis.

Intervention evidence: Both cervical pessaries, cerclages and progesterone are interventions for preventing preterm birth in women with short cervixes. A 2013 Cochrane review included only one RCT for cervical pessary, which was conducted in Spain. The results from this RCT showed a significant decrease in spontaneous preterm birth (RR=0·36 [0·27-0·49]).(88) Cervical cerclage has also been shown to reduce preterm birth; a meta-analysis from 2011 cited a RR=0·70 (0·58-0·83).(89) In a more recent meta-analysis, both cervical pessaries and vaginal progesterone had significant reductions in preterm birth (RR=0·32 [0·12-0·89] and RR=0·52 [0·36-0·73], respectively), but cervical cerclage did not (RR=0·77 [0·46-1·25]).(90)

MODEL INCLUSION: RR=6·19 (3·84-9·97). Consistent evidence based on observational data, supporting evidence for intervention effect for multiple interventions.

Fetal fibronectin

Risk: The study conducted in three US medical centers included women who experienced PPROM (n=65) or preterm contractions with intact membranes (n=117) and controls. The authors found that in women who had preterm contractions and cervical/vaginal fibronectin present were more likely to have infections and/or antepartum bleeding. Women who delivered before term who were negative for fibronectin had no history of infections.(43)

A quote from the authors of another paper on the relationship between fetal fibronectin and spontaneous preterm birth: “Fetal fibronectin is a glycoprotein found in amniotic fluid, placental

tissue, and the extracellular substance of the decidua basalis next to the placental intervillous space. It is thought to be released through mechanical or inflammatory mediated damage to the membranes or placenta before birth".[91] As the authors note, the presence of fetal fibronectin is likely indicative of another process, rather than an independent risk factor itself.

Quality of risk estimate: Very low. The study examining the risk relationship between fetal fibronectin and preterm birth has a very small sample size and was conducted in the United States. Furthermore, the authors demonstrated that fibronectin presence was linked to a previous uterine infection or episode of bleeding. The commentary from the second paper also notes that the presence of fetal fibronectin is likely due to previous damage to the fetal environment (membranes or placenta).

MODEL INCLUSION: Not included in the model. The evidence indicates that while the presence of fetal fibronectin can be predictive of a preterm birth, it is not an independent risk factor for the birth outcome but rather an intermediate outcome, indicative of other processes.

Early vaginal bleeding

Vaginal bleeding has been shown to be associated with uterine abnormalities, hormone dysfunction, and reproductive tract infections. Some of the risk factors may act on bleeding directly, some through other mechanisms (lead to abnormal placentation, cervical erosion, etc). The study below reported significant associations (adjusted ORs) between first trimester vaginal bleeding and many reproductive tract infections (Trichomonas vaginosis, chlamydia, BV, and GBS, though GBS wasn't statistically significant). Women with more infections had much higher risk of bleeding. Women who had BV in the first trimester were 2x more likely to have 1st trimester bleeding than women who didn't have BV. The authors reported that 17·4% of PTB could be prevented with treatment of BV associated with 1st trimester bleeding. 16·9% of PTB could be prevented with treatment of BV even without 1st trimester bleeding presence.[92] Given this evidence, we are concluding that PTB is more associated with BV rather than 1st trimester bleeding.

The authors examined risk factors for vaginal bleeding and found the following results: working during pregnancy and vaginal bleeding OR=1·2 (1·0-1·5); Ovarian cyst and vaginal bleeding OR=1·6 (1·1-2·2); cervical inflammation/erosion/infection and vaginal bleeding OR=1·7 (1·3-2·3). Fibroids and vaginal bleeding OR=1·4 (0·9-2·1). For recent occurrence (within 6 months of conception) fibroids had an OR=2·2 (1·1-4·2) and cervical issues had a higher OR=2·1 (1·2-3·5). They found light bleeding not to be associated with PTB, but heavy bleeding was associated. In their discussion the authors write "The nature of the characteristics that were associated with an increased risk of bleeding— maternal age, previous spontaneous or induced abortion, cervical inflammatory conditions, fibroids, and ovarian cysts— suggests that bleeding, in some instances, is a symptom of existing uterine pathology or maternal physiologic changes that accompany aging."[93]

A meta-analysis reported a risk of spontaneous preterm birth due to early vaginal bleeding equal to OR=1·62 (1·29-2·02), which is the pooled results of 3 studies.[44] This analysis does not discuss upstream factors from vaginal bleeding.

The article states in its introduction that half of vaginal bleeding episodes during pregnancy have no known cause. The cascade from vaginal bleeding to preterm birth is thought to be because of thrombin production, which causes uterine contractions. Furthermore, vaginal bleeding could be caused by inflammation or infection of the uterus, or later in pregnancy because of placental abnormalities. The authors estimated that roughly 30% of preterm births were attributable to vaginal bleeding. It was reported that any bleeding was related to PPROM (RR=1·7 [1·0-2·9]), and preterm labor (RR=1·4 [1·0-2·1]). First trimester only bleeding was significant for PPROM (RR=1·9 [1·1-3·3]), but not for preterm labor (RR=1·3 [0·3-2·0]). Second trimester bleeding showed no impact on PPROM or preterm labor (both RR=1). Any first or second trimester bleeding was not associated with medically indicated preterm births. Recurrent bleeding and bleeding in both trimesters were associated with higher risk of PTB.(94) The analysis controlled for potential confounders from this analysis of the prospective cohort.

Quality of risk estimate: Low. The risks are from both single studies and meta-analyses. Some, but not all, of the articles listed above controlled for potential confounders in their analysis

MODEL INCLUSION: Not included in the model. The evidence demonstrates that while vaginal bleeding is an indicator of risk of preterm birth, it is not an independent risk factor. Rather, it is indicative of other processes, such as infection or placental abnormalities.

Placental abruption

Risk: A cohort of singleton births at Mount Sinai Hospital in New York City was followed from January 1986 to December 1996, resulting in a total sample size equal to 53,371 births. Of these, 530 of the pregnancies were complicated with placental abruption. The authors reported adjusted relative risks for all preterm births as well as spontaneous preterm labor, membrane rupture and provider initiated. The overall risk of PTB with placental abruption was equal to RR= 3·90 (3·50-4·40). The risks for premature membrane rupture, spontaneous preterm labor and medically indicated were RR= 2·70 (2·00-3·60), RR=6·60 (5·40-7·90), and RR= 5.00 (3.60-6.90), respectively. Placental abruption was more common in black women, women who had at least four children, who smoked or used drugs, and women who were on medicaid. The authors also demonstrated that as the degree of placental separation increased, the risk of preterm birth increased as well.(45)

Quality of risk estimate: Very low. The authors controlled for many confounders (clinic vs. private patient, maternal age, parity, race, smoking, drug use, pre-eclampsia, and chronic hypertension) in the analysis, however the risk estimate is only from a single study in a high-income setting.

MODEL INCLUSION: Not included in the model due to lack of available data on placental abruption prevalence in LMIC settings.

Placenta previa

Risk: A cohort in Syria compared birth outcomes for women with "major" (complete or partial placenta previa) and minor (marginal previa or low lying placental) placenta previa. All women with "major" previa received C-sections. The minor group was the comparison group.(46) This indicates that placenta previa is likely a risk factor for provider initiated preterm birth, however it is not stated whether or labor had begun prior to C-section.

A note: Placenta previa complicates 0·3-0·5% of pregnancy cases, so in the model this would make little to no difference even if there was a risk relationship with spontaneous preterm birth, which appears to be absent.(46)

Quality of risk estimate: Very low. The risk estimate is from a single study that did not adjust for any potential confounders in the analysis.

MODEL INCLUSION: Not included in the model due to unclear relationship with spontaneous preterm birth and lack of data

Uterine abnormalities

Risk: A retrospective cohort at Mount Sinai hospital in New York of 158 single pregnancy cases with uterine abnormality (major and minor fusion defects) and 158 unexposed singleton births. The authors examined spontaneous and medically indicated preterm birth. Comparison of baseline characteristics show that the groups are comparable. For spontaneous preterm birth, minor fusion defects are not statistically associated (p=0·498), but major fusion defects are (p=0·004). Indicated preterm births are statistically associated with minor defects (p=0·025), but not major (p=0·160). A C-section delivery was associated with both minor and major fusion defects.(95)

A systematic review does not differentiate between types of PTB, but shows a significant relationship between multiple types of uterine congenital abnormalities and PTB. Canalization defects had a RR=2·14 (1·48-3·11) and unification defects a RR=2·97 (2·08-4·23).(96)

Quality of risk estimate: Very low. There was no adjustment for potential confounders in the analysis in the NYC study. For the review the analysis does not differentiate between spontaneous and provider initiated preterm birth, which based off the findings from the NYC study, makes quite a difference in the analysis.

MODEL INCLUSION: Not included in the model due to unclear relationship with spontaneous preterm birth and lack of data

Uterine fibroids

Risk: A multi-center survey in China (N=>112,400 pregnancies) examined the risk between uterine fibroids and preterm birth. Compared to women without uterine fibroids, those with fibroids had no increased risk of preterm birth (OR=1·0 [0·9-1·2]), but a significantly increased risk of cesarean delivery (OR=1·7 [1·6-1·9]).(47) The preterm risk analysis controlled for maternal age, parity, BMI, pregnancy complicating hypertensive disorders, GDM, PPROM,

tobacco and alcohol use, and prior preterm birth. The cesarean delivery analysis excluded pregnancies complicated by placenta previa, breech presentation, and placental abruption and controlled for maternal age, parity, BMI, pregnancy complicating hypertensive disorders, PPROM, neonatal weight, and gestational age at delivery. Therefore, it appears that there is no relationship between uterine fibroids and preterm birth and any association that may be seen is likely due to provider-initiated C-section.

There is a meta-analysis that reports an unadjusted OR= 1·5 (1·3-1·7), however the authors of the meta-analysis note that in five of the six included studies there was increased hospitalization in these patients due to pain syndrome, which potentially led to “a decision to proceed with an early delivery”.[97] While not explicitly stated, this increased risk appears to be for provider-initiated preterm birth, not spontaneous preterm birth and therefore not included in our model.

Quality of risk estimate: Very low. The primary study has a large sample size and controlled for many potential confounders in the analysis.

MODEL INCLUSION: Not included in the model due to unlikely relationship with spontaneous preterm birth, given the evidence.

Pregnancy-Related Morbidity

Gestational diabetes

Risk: A prospective cohort study in California (N=46,230 pregnancies between June 1996 and June 1998) examined the risk of gestational diabetes and spontaneous preterm birth. The data were collected from medical record review, but the authors did conduct validation procedures on a subset of the records to ensure that they were accurate. Women who had diabetes prior to pregnancy were excluded from the study. All women in the study had a 50g, 1-hour oral glucose test administered at 24-28 weeks gestation and based on the results of this were classified into four groups: Normal screening (<140 mg/dL), Abnormal screening (1-hour test of at least 140 mg/dL and 100mg 3-hour test that was normal), Carpenter-Coustan (at least two tests above the cutoffs, but did not meet National Diabetes Data Group thresholds), and GDM (two tests at or above NDDG’s cutoffs). The authors demonstrated the risk of spontaneous PTB increased as severity of maternal glycaemia increased. The authors report an adjusted RR= 1·42 (1·15-1·77), comparing women with GDM according to NDDG’s cutoffs to women with normal test results. This model was adjusted for age, race–ethnicity, preeclampsia–eclampsia–pregnancy-induced hypertension, chronic hypertension, polyhydramnios, and birth weight for gestational age. They did a sub-analysis where they removed the births that were classified as spontaneous but resulted in a C-section to ensure they were not including possibly provider initiated births, which increased the risk estimate to RR=1·86 (1·44-2·42). In the discussion the authors acknowledge that their findings are not consistent with earlier studies that found no association between GDM and PTB, but these studies all had a very small sample size (number of preterm events did not exceed 32 in any of them), so it may be possible that the lack of association is due to a beta error.[28]

Quality of risk estimate: Medium-Low. The risk estimate is from a single study rather than a meta-analysis. However, the sample size for the study is quite large and they controlled for some important confounders in their analysis. The study was conducted in the United States, which means it may not be generalizable to the countries in our analysis. Furthermore, there are additional supportive observational studies (see below) and was specifically examining spontaneous preterm birth.

Additional observational risk: The study excluded women who had a diabetes diagnosis requiring treatment during or prior to current pregnancy and had a total sample size of 23,316 pregnant women. The study used three measures for maternal glucose; Fasting, 1-hour and 2-hour plasma glucose levels. For the secondary outcomes (premature delivery being a secondary outcome) the odds ratios were estimated for an increase of one standard deviation in each of the three levels (fasting- SD= 6.9mg/dL increase; 1 hour-SD=30.9mg/dL increase; 2-hour- SD=23.5 mg/dL increase). The model for premature delivery (<37 weeks) adjusted for age, body-mass index (BMI), smoking status, alcohol use, presence or absence of a family history of diabetes, gestational age at the oral glucose-tolerance test, sex of the infant, parity (0, 1, or ≥2, except for primary cesarean deliveries), mean arterial pressure, and presence or absence of hospitalization before delivery. Using the three measures of maternal glucose, the OR for preterm birth varied; fasting plasma glucose (FPG) OR=1·05 (0·99-1·11); 1-hour plasma (1-PG) glucose OR= 1·18 (1·12-1·25); 2-hour plasma glucose (2-PG) OR=1·16 (1·10-1·23). Additionally, maternal glucose levels are consistently associated with pre-eclampsia in the analysis across all three measures of maternal glucose; the FGP OR= 1·21 (1·13-1·29), 1-GP OR=1·28 (1·20-1·37), and the 2-GP OR=1·28 (1·20-1·37).[98]

Intervention evidence: Cochrane reviews on various treatments (insulin, lifestyle alterations like diet and exercise, other anti-diabetic medication) state that there is inconclusive evidence on treatment effect on preterm birth.[99],[100] Individual studies found no difference in gestational age at birth between treatment and intervention groups.[101], [102]

MODEL INCLUSION: RR=1·42 (1·15-1·77). Consistent evidence based on observational data, but no evidence supporting an intervention effect.

Pre-eclampsia

Risk: The analysis cites a risk for spontaneous PTB equal to OR=1·98 (1·80-2·18), which was estimated using a multilevel multivariate logistic regression model. The multilevel analysis was structured on three-levels (individual, health facility, country) with random intercepts, and adjusted for maternal age, marital status, education, parity, and previous caesarean section. This measure is from a secondary analysis of 16,474 singleton preterm births collected by the WHO multi-country survey on maternal and newborn health and published in 2014.[20] This analysis cited a pre-eclampsia prevalence in their population of 5%, which was used to convert the odds ratio to a risk ratio equal to RR=1·89 (1·73-2·06). This estimate was adjusted to account for the relationship with calcium deficiency, which is also included in the model. Calcium supplementation reduces risk of pre-eclampsia by 55%,[17] therefore the pre-eclampsia relative risk included in the model is equal to 1·40 (1·32-2·02).

Quality of risk estimate: Medium. The WHO multi-country survey was conducted in facilities in LMICs, which could limit its generalizability to the general population. The authors adjusted for multiple potential confounders in the analysis.

Intervention evidence: See above for calcium supplementation

Note: Pre-eclampsia is an indicator for provider-initiated (iatrogenic) preterm birth. However, there are more upstream risk factors associated with the incidence of preeclampsia and preterm birth, which leads us to believe that preeclampsia is partially an intermediate outcome along this casual pathway. Calcium supplementation is the recommended antenatal treatment for preeclampsia. The WHO ran an RCT of calcium supplementation (1·5 g/day) in women with low calcium diets (<600 mg/d) in LMIC settings. Their primary outcomes were pre-eclampsia and preterm birth. The women were all also nulliparous, which is important because parity plays a role in preeclampsia and preterm birth, so this restriction reduces potential confounding. The analysis adjusted for maternal age, BMI, gravidity, and compliance as well as accounted for clustering of the facilities. Additionally, women with chronic hypertension were excluded. The results showed that calcium supplementation reduces the severity of pre-eclampsia, but did not prevent it entirely.(103) This fits with our assumption for this model that Ca deficiency is just one cause of preeclampsia. The risk for preterm birth was reduced in this trial, but the reduction was not statistically significant.

MODEL INCLUSION: RR=1·40 (1·32-2·02). Consistent evidence from observational studies with supporting evidence from some randomized controlled trials for one possible intervention.

Maternal infections

HIV

Risk: The estimate of risk for HIV and preterm birth is from a large meta-analysis of 52 cohort studies conducted in Africa. The analysis found an OR of 1·56 (1·49 - 1·63). These studies looked at pregnant HIV-infected women with and without ARV therapy (N=200,896) and compared them to HIV-negative women. There was adjustment for potential confounders in roughly half of the studies included. There was no significant difference in risk of preterm births between HIV+ women with and without ARV therapy. Using an average of the prevalence of the countries included in the meta-analysis (average prevalence=7.85%), the odds ratio was converted to an estimated risk ratio equal to RR= 1·49 (1·43-1·55).(29)

Quality of risk estimate: Medium. The risk estimate is from a meta-analysis that included 40 cohort studies (both prospective and retrospective) included in pooled OR analysis comparing HIV infected women to non-infected women to determine the association between HIV status and preterm birth. The inclusion of strictly cohort studies, some of which adjusted for potential confounders in the analysis, earns this meta-analysis a "medium-low" grade for quality.

Intervention evidence: For an intervention effect, sub-group analyses compared ORs for women who did take ARVs before and/or during pregnancy to those who did not. ORs overlapped: ARV OR: 1·77 (1·55-2·02), No ARV OR: 1·54 (1·23-1·92). It is not explicitly stated in the paper, but

for these comparisons we believe the comparison group was HIV-negative women, as in the primary analysis.[29]

Note: There is mixed evidence on whether or not ARV use is associated with preterm birth. This includes both initiation of ARVs preconception and antenatally. Trends from individual high incomes countries differ from those in LMIC settings and there appear to be differences in risk of preterm birth associated with the type of therapy (protease inhibitors or not) and when the therapy was initiated (preconception or antenatally).[104-106]

MODEL INCLUSION: RR=1·49 (1·43-1·55). Consistent supporting evidence based on observational data, no supporting evidence on intervention effect

Malaria (falciparum and vivax)

Risk: The primary risk estimate for falciparum malaria is from a 2014 analysis of the WHO Global Survey to assess risk factors for spontaneous and provider initiated preterm births. The data set included 172, 461 singleton deliveries in 22 LMICs.(1) The authors used a multi-level model to address heterogeneity across the different samples and controlled for other risk factors such as height < 145cm, HIV, UTI, diabetes and pre-eclampsia. For malaria, the sample included 30,544 liveborn singleton deliveries in African countries. The authors reported an adjusted OR=1.67 (1.32 – 2.11). This is converted to a risk ratio (RR)=1.56 (1.28 – 1.90) using the average malaria prevalence (10.6%) across the six African countries included in the analysis.

Additional supporting evidence for the estimate of risk of preterm birth is based on a Steketee et al. review of previous work investigating the link between malaria and birth outcomes.[107] They found an OR= 2·85 (2·20 – 3·50) from three cohort studies. The studies are all observational and all demonstrate risks of similar magnitude. There is some variation in the definitions of malaria (cord parasitemia, placental infection, etc.) and differences in magnitude of the association between women with different characteristics, primarily primigravid women. The risk estimate in the model is a summative estimate from Steketee et al. that includes pregnant women of all characteristics

Studies that looked at the relationship between vivax malaria infections found an effect on low birth weight. However, studies that looked at gestational age found little evidence that there is an increased risk of premature births.[108] In this analysis we will assume no risk of premature birth for vivax malaria.

Quality of risk estimate: Medium. This study has a large sample size, was conducted recently in multiple LMICs and specifically measures risks associated with spontaneous preterm birth. Furthermore, the multi-level model and controlling for key confounders strengthens the findings.

Intervention evidence: There have been numerous studies that have investigated the impact of IPTp on LBW, but none have sufficient evidence to estimate efficacy in terms of reducing the risk of preterm birth. It should be noted that a recent Cochrane review found a 19% reduction in low birth weight.[109] The treatment studies demonstrate a reduction in low birthweight, but do not specifically look at preterm birth. However, given the difficulty assessing gestational age in

malaria endemic settings, the strength of association between these treatments and LBW can strengthen the association with preterm birth, but cannot be used for an efficacy estimate.

MODEL INCLUSION: RR=1.56 (1.28 – 1.90). Consistent evidence based on observational data, no evidence supporting an intervention effect

Syphilis

Risk: The associated risk between maternal syphilis infection and preterm birth was estimated to be RR= 3·22 (3·15-3·23) in a meta-analysis of 34 observational studies. Most studies were conducted in high-income settings. The meta-analysis reported incidence of preterm birth among women with syphilis (23·2%, 18·1-29·3%) and incidence among women without syphilis (7·2%, 5·6%-9·3%) using random-effects models. The relative risk was then calculated using these reported estimates. This paper also demonstrated that if the women with syphilis were treated in the first trimester of pregnancy, excess risk of preterm birth was essentially eliminated.[30]

Quality of risk estimate: Medium. In the meta-analysis for the risk estimate, there was no controlling for potential confounders. For the meta-analysis of treatment trials (see below), the authors noted a lack of controlling for confounding in 7 of the 9 studies but given the magnitude of the reduction gave the estimates a strong GRADE recommendation**.** Despite the lack of controlling for confounders, the relationship is strengthened by the treatment effects from both meta-analyses (some of which controlled for confounding).

Intervention evidence: The effectiveness estimated for treatment is from a meta-analysis of observational treatment trials that found that using at least 2·4MU penicillin resulted in a 64% reduction in preterm birth (RR: 0·36 [0·27, 0·47)]). There were 9 studies included in this analysis, seven of which did not control for any confounders in the analysis.[110]

MODEL INCLUSION: RR=3·22 (3·15-3·23). Consistent observational evidence with supporting intervention evidence from observational studies.

Chlamydia

Risk: The risk of preterm births from a 2018 meta-analysis that included 25 studies, of which only eight reported adjusted ORs, was estimated to be OR = 1·27 (1·16, 1·40). The meta-analysis includes 21,801 pregnant women with chlamydia, the total number of subjects was 614,892 and the estimated prevalence across the 25 studies was 3·54%, which was used to convert the odds ratio to an estimated RR= 1·26 (1·15, 1·38). The analysis included both prospective cohorts and case-control studies.[31]

Quality of risk estimate: Low. The papers included in the meta-analysis for the risk relationship are all observational, comparing pregnant women with and without chlamydia. There were differences in laboratory methodology to diagnose the infection across the different studies, combined with the different studies designs resulted in high heterogeneity. Additionally, only some of the included studies controlled for potential confounders. The sample size was quite large, pooled across the 25 studies (614,892). There is an absence of treatment effect from the

Cochrane review (see below), however this is from a small RCT and was likely underpowered to find an effect on preterm birth. Therefore, we are considering the risk relationship and no treatment effect.

Intervention evidence: The 2017 Cochrane review on treatment of chlamydia during pregnancy included only one study that examined the effect of erythromycin treatment vs. placebo on preterm birth, which demonstrated an insignificant reduction in preterm birth (RR=0·90, 0·56-1·46) and was determined to be of low quality.[111]

MODEL INCLUSION: RR= 1·26 (1·15-1·38). Consistent evidence based on observational data; no supporting intervention effect due to limited availability of evidence

Trichomonas vaginalis

Risk: There are very few studies examining T. vaginalis infection during pregnancy and preterm birth alone. A multi-site prospective study conducted in 1996 in hospitals in the US enrolled 13,816 pregnant women during their second trimester who were then tested for T. vaginalis. They found an increased risk (RR=1.30, 1.10-1.40) of preterm birth in women who tested positive.[48] The study controlled for some potential confounders and used sensitive lab methods for the time period.

Quality of risk estimate: Very low. The study had a very large sample size, which was obtained through screening of all pregnant women at the hospitals in the United States. Furthermore, the study controlled for important potential confounders in their analysis. However, the generalizability of this patient population to the population in this analysis is likely quite low.

MODEL INCLUSION: Not included in the model due to minimal supporting evidence and concerns of generalizability.

Asymptomatic bacteriuria

Risk: The 1989 meta-analysis examined the relationship between asymptomatic bacteriuria and preterm birth. There were 4 cohort studies (N=6,034) included in the analysis, which compared the rate of premature births in non-bacteriuric women to untreated bacteriuric women, which demonstrated a risk relationship equal to RR=0·51 (0·36-0·69). The studies matched groups on factors such as maternal age, race, parity, and SES. The meta-analysis also included RCTs of treatment and the relationship with LBW (where there was a significant reduction), however, they did not look specifically at treatment and preterm birth. The inverse of the RR is equal to RR=1·96 (1·45-2·77).[32]

A 2009 retrospective study of 199,093 singleton deliveries in the Soroka University Medical Center in Israel between the years 1988-2007 reported an adjusted OR=1·6 (1·5-1·7).[112] In a 2008 review the authors report that while screening for and treatment of asymptomatic bacteriuria during pregnancy is a standard guideline, there have been very few recent studies (post 1970s) examining the relationship.[113]

Quality of risk estimate: Medium-low. The studies included in the risk analysis are observational cohort studies and there was controlling for confounding in the analysis. The studies are not conducted in low- and middle-income countries, so there is concern about the generalizability of the findings to the countries included in our analysis.

Intervention evidence: A 2019 Cochrane review that included three randomized controlled trials demonstrated a 66% reduction in the risk of preterm birth (RR=0·27 [0·13-0·88]) if the mother was treated for asymptomatic bacteriuria.(114) The sample size for the trials is very small; the three trials combined have a total of 347 participants. Furthermore, the studies included were deemed to be of low quality due to design limitations concerning allocation, blinding, and heterogeneity between the two study populations. A recent cluster randomized trial showed no effect of population-based screening and treatment for abnormal vaginal flora and UTI in pregnancy. The incidence of PTB was 21·8% in the intervention group and 20·6% in the control for a risk ratio equal to RR= 1·07 (0·91-1·24).(115)

The above risk from the cohort studies is included in the analysis. The conflicting results from treatment trials does not lead to a conclusion that there is a proven effect.

MODEL INCLUSION: RR=1·96 (1·45-2·77). Consistent evidence based on observational data, no consistent evidence supporting an intervention effect

Periodontal disease

Risk: The risk of preterm birth associated with periodontal disease is RR=1·61 (1·33-1·95), taken from a 2016 meta-analysis of 16 studies (N=8,594 pregnant women). The 16 case-control and prospective cohort studies had slight variation in exposure definition, though most referred to the same classification. Most studies controlled for confounders, though the exact confounders controlled for varied across studies.(33)

Quality of risk estimate: Medium. The meta-analysis of observational studies had slight variation in exposure definition and most studies controlled for confounders, though the exact confounders controlled for varied across studies. The direction of the relationship between periodontal disease and preterm birth is consistent across the above studies, but the magnitude of the relationship differs.

Intervention evidence: A Cochrane review included 11 randomized controlled trials comparing treatment of periodontal disease with no treatment during pregnancy. The total sample size was equal to 5,671 participants. This analysis demonstrated an insignificant reduction in preterm birth with treatment of periodontal disease (RR=0·87, [0·70-1·10]).(116) There was controlling for potential confounders in the analysis of the individual studies.

An older meta-analysis by George et al. included 10 trials with a total sample size of 5,645 pregnant women. The studies were conducted in both LMICs and developed countries and demonstrated a RR= 0·65 (0·46-0·93) of preterm birth comparing treated to untreated pregnant women The majority of studies included in the treatment analysis controlled for important

confounders/risk factors in addition to controlling for confounding through the study design.(117)

The treatment effect meta-analyses included most of the same studies; there are three papers included in the Cochrane review that were not included in the George et al. analysis and two papers included in George et al. but not the Cochrane. The inclusion or exclusion of these studies affected the magnitude of the treatment effect as well as the statistical significance of the association. The studies included in the Cochrane review were deemed to be of "low" quality due to bias potential and heterogeneity.

The heterogeneity in the treatment meta-analyses appears to be a result of different case definitions and interventions across the trials. None of the studies included in the Cochrane review use the same case definition nor the same intervention (certain components were the same across the studies, but there were no two studies with identical components). In the George et al. meta-analysis, two sets of two studies (out of 10) had the same case definition. Similarly, the interventions all involved scaling and/or root planing and/or an educational component, but the details of these differed across studies (type of procedure, number of visits, additional steps like chlorhexidine mouthwash, etc.). Furthermore, severity of periodontal disease increases with maternal age,[(118)] so part of the lack of treatment effect could potentially be explained by the fact that younger women are more often the pregnant women who are in these treatment trials. Therefore, it is less likely that the treatment will have an impact, as the disease is not severe. Additional studies that are more comparable and take into account potential co-risk factors, such as maternal age, are needed in order to reach a conclusion on treatment effectiveness on reducing preterm birth.

MODEL INCLUSION: RR=1·61 (1·33-1·95). Consistent evidence based on observational data, but no consistent evidence supporting an intervention effect

Bacterial Vaginosis

Risk: A meta-analysis that included 24 studies reported the risk of preterm birth associated with asymptomatic bacterial vaginosis as OR=2·16 (95% CI: 1·56-3·0), estimated using a random effects model. It is not stated in the methods if the odds ratios were adjusted or not, but when the individual studies were examined it was clear that some, but not all, adjusted for potential confounders. This meta-analysis included 24,190 patients. The average prevalence of 27% calculated across the countries included in the analysis was used to convert the OR to an estimated RR=1·64 (1·36-2·33).[(34)]

Quality of risk estimate: Medium-Low. There are 24 cohort studies included in the meta-analysis examining the risk relationship, resulting in a large sample size. However, there are few studies in low- and middle-income countries, which means the findings may not be completely generalizable to the countries included in the model. There is no discussion of controlling for confounders in the analysis in the meta-analysis itself, but upon investigation of the studies included, there was controlling for potential confounders in some of the studies.

Intervention evidence: The 2013 Cochrane review on treatment of bacterial vaginosis during pregnancy demonstrates an insignificant reduction in the risk of preterm birth comparing any treatment vs. no treatment (OR: 0·88 [0·71-1·09]).[(119)] This meta-analysis of treatment trials included 13 trials (many for different antibiotic treatments) with a total number of participants equal to 6,491. Another analysis of a cluster randomized trial revealed that of women with abnormal vaginal flora (AVF), one third experienced persistent AVF after treatment with clindamycin. These women experienced higher rates of adverse pregnancy outcomes, preterm birth included, as compared to women with AVF who were treated and cured.[(120)] The presence of persistent AVF may explain the discrepancy between the risk observed in the observational studies and the absence of treatment effects demonstrated in the trials.

MODEL INCLUSION: RR=1·64 (1·36-2·33). Consistent evidence based on observational data, but no consistent evidence supporting an intervention effect

Group B Strep colonization

Risk: The risk of group B strep colonization on preterm birth is from a 2017 meta-analysis of 45 studies, which included 31 cohort studies, 6 cross sectional studies, and 11 case control studies. Maternal GBS colonization was defined by presence of GBS in vaginal, cervical, and/or rectal swabs. The analysis examined separately studies that defined GBS presence by isolating GBS from urine samples.[(35)] The analysis presented findings multiple ways. The first included both the cohort and cross sectional studies (using the swab-colonization definition of exposure, which is the standard sampling method) and found a RR=1·21 (0·99-1·48) (28 studies). These studies were largely hospital based studies, either cross-sectional or retrospective cohorts, with few concerns about selection bias (aside from being not a random population sample). Examining just the case-control studies (same exposure definition, 9 studies), an OR=1·85 (1·24-2·77) was reported. However, only one of the case-control studies described a matching process and this study only matched on maternal age. The other eight implemented strict exclusion criteria, which may limit generalizability.

There were 11 studies that defined GBS from urine isolates. The 9 cohort studies reported an RR=1·98 (1·45-2·69) and the 2 case control studies reported an OR= 1·96 (0·65-5·98). Presence of maternal bacteriuria indicates denser colonization than the swabbing techniques, which may explain the stronger association (at least amongst the cohort studies). However, this risk represents the risk of asymptomatic bacteriuria caused by GBS, not risk of GBS colonization alone. As we already have asymptomatic bacteriuria considered, this risk presented above (RR=1.98) is excluded as not to be repetitive.

Currently, the analysis with both cohort and cross sectional studies (RR=1·21 [0·99-1·48]) is considered, as this estimate is for GBS colonization and has the fewest methodological concerns.

Quality of risk estimate: Medium-Low. Although the meta-analysis for the risk relationship included 45 studies, there was variation in how the exposure was identified (swab vs. urine sample), which impacted the magnitude and significance of the risk relationship (diagnosing disease via urine sample provided a stronger relationship that was statistically significant), though these were analyzed separately. There was adjustment for confounders in the analysis of

some of the studies, others were not adjusted. Furthermore, the individual studies' results are quite varied, with very wide confidence intervals and variation in the point estimate. There is inconsistency in the magnitude of the treatment effects (one shows very large reduction, others do not), but they are all in the same direction, just not all statistically significant.

Intervention evidence: A study from 1987 demonstrated that treatment with penicillin of group B streptococci present in urine in pregnant women reduced preterm birth rates from 38% in placebo group to 5.4% in treatment group, which translates to a relative risk of 0·14.[121] Two other studies demonstrate a reduction in risk of preterm birth with treatment, but this reduction is not significant: RR=0·90 (0·60-1·30),[122] and RR=0·75 (p=0·16, 95% confidence interval not listed, but small sample sized acknowledged).[123] The majority of recent treatment studies published investigate the use of intrapartum antibiotics to prevent colonization in the infant. Both ACOG and the WHO recommend intrapartum treatment of GBS with antibiotics in order to prevent vertical transmission.[124],[125] However, WHO states that more research is needed to determine the impact of GBS screening and treatment on preterm birth.

MODEL INCLUSION: Extended Model 2. RR=1·21 (0·99-1·48). Inconsistent and weak risk association based on observational data, no consistent supporting evidence for an intervention effect

Helminth Infection

Risk: The risk is from a Cochrane review on the treatment of helminths in pregnant women, which reported a treatment effect of RR=0·88 (0·43-1·78, two trials, 1318 total participants). The inverse of this treatment effect is equal to 1·13 (95% CI: 0·56-2·33).[49]

Quality of risk estimate: Medium. The two studies included in Cochrane review are small and in one of the studies are there concerns about the possibility of selection bias.

MODEL INCLUSION: Not included in the model due to evidence of no risk for preterm birth

Influenza Infection

Risk: A prospective, multi-season cohort study of pregnant women in Bangkok, Thailand, Lima, Peru, and Nagpur, India was conducted. In 2017 and 2018, cohorts of pregnant women were enrolled starting up to 10 weeks before the anticipated start of the influenza season until the fourth week of the influenza season. Influenza was diagnosed via real-time RT-PCR testing of the nasal swabs from symptomatic women. 10,826 women had complete birth data, 196 (11%) of whom had a preterm birth. For the PTB analyses the model was adjusted for smoking, alcohol use, abbreviated wealth index, age, parity, education, chronic disease, HIV infection, gestational diabetes and hypertension, and gestational age at first visit. Having an influenza episode during pregnancy was not associated with an increased risk of preterm birth (aHR 1.4, 95% CI 0.9 to 2.0) Findings were consistent in sensitivity analyses.

Quality: Medium. The study had a large sample size in multiple LMICs, a clear exposure definition and the analysis controlled for multiple important confounders.

MODEL INCLUSION: Not included in the model due to evidence of no risk for preterm birth

Other

Genetic Component of risk of Preterm Birth

Risk: There has been extensive work primarily through animal models looking at the genetic links to preterm birth. These studies along with more recent twin studies were reviewed by Bezold et al.[(126)] While there are some outliers in findings, overall most studies find a large genetic component of the risk of preterm birth, all carried through the maternal side.

There have also been several studies using twin registries, comparing PTB rates between monozygotic and dizygotic twins.[(127-129)] In these studies, using slightly different analytical methods, the genetic component of PTB were all linked to the maternal genetic characteristics. The three studies suggested that between 27% and 36% of the risk of preterm birth was linked to genetics. All three studies were in high-income countries (Australia, Netherlands and Sweden) and note that the cohorts have few other risks *for* preterm birth, suggesting that the genetic role in risk of preterm birth would be lower in populations with higher exposure to other risk factors.

Quality of risk estimate: Very low. The majority of evidence is still in animal models from single studies and exact relative risks have yet to be quantified.

MODEL INCLUSION: Not included in the model due to lack of data

3. Sources and Quality of Prevalence Estimates

This section contains information of the prevalence of the 26 risk factors included in the model. The source of the prevalence estimate is listed first; second is the quality of the source; third is an explanation of process to correcting the prevalence estimates for treatment, if applicable.

HIV

Prevalence: We used the UNAIDS country-specific estimates of the number of women in need of PMTCT divided by the number of births that year to get the % of HIV positive pregnancies.[(130)] For countries without HIV estimates, an assumed prevalence of 0·1% was included in the model. Data from UNAIDS and birth estimates did not have corresponding 95% confidence

intervals, therefore a confidence interval with a width of 10% was constructed around the point estimate.

Quality: High. Country-specific data collected by UNAIDS

Malaria

Prevalence: We were not able to find good estimates of falciparum malaria prevalence in pregnant women. To estimate this, we used country-level rates of malaria incidence for all ages and estimates of percent of malaria due to falciparum. The estimates of malaria incidence and percent due to falciparum are from the World Malaria Report 2018.(131) We are assuming that these current estimates reflect overall malaria control methods in countries including LLTN, indoor residual spraying and other vector control strategies. The coverage and effectiveness of IPTp in Sub-Saharan African countries was used to adjust the final estimates of the prevalence of pregnancies exposed to falciparum malaria.

Quality: Medium. Country-specific data collected by WHO, but not specific to pregnant women

Syphilis

Prevalence: The prevalence data is from a 2015 analysis including 63 studies on the global and regional prevalence of three sexually transmitted infections in women of reproductive age (WRA). The regional prevalence and corresponding 95% confidence intervals were used for this estimate.(132)

Quality: Low. Regional data and only in WRA, not pregnant women specifically.

Treatment: The effectiveness estimated for treatment is from a meta-analysis that found that using at least 2·4MU penicillin resulted in a 64% reduction in preterm birth (RR: 0·36 [0·27-0·47)]).(110) There were 9 studies included in this analysis, seven of which did not control for any confounders in the analysis. Coverage of syphilis screening and detection during ANC is from the LiST database. For countries without an estimate of syphilis screening and detection, coverage of ANC4+ was used as a proxy measure, with the understanding that this coverage measure will overestimate the coverage of syphilis treatment. The ANC4+ coverage is also from the LiST database. To account for some of the overestimation, the median values from a study on quality of ANC service provision in Sub-Saharan Africa on % of facilities prepared to deliver syphilis testing and treatment to lower the coverage to a more realistic level (=coverage of ANC4+ * % of facilities prepared to deliver syphilis T&T).(133)

Example of treatment adjustment calculation: Treatment adjusted prevalence of women with syphilis = % of women with syphilis - (% of women with syphilis * coverage of syphilis detection and treatment OR ANC4+ * effectiveness of syphilis treatment on preterm birth).

Chlamydia

Prevalence: The prevalence data are from a 2015 analysis including 63 studies on the global and regional prevalence of three sexually transmitted infections in women of reproductive age. The regional prevalence and corresponding 95% confidence intervals were used for this estimation.(132)

Quality: Low. Regional data and only in WRA, not pregnant women specifically.

Treatment: The 2017 Cochrane review on treatment of chlamydia during pregnancy included only one study that examined the effect of erythromycin treatment vs. placebo on preterm birth, which demonstrated an insignificant reduction in preterm birth (RR=0·90 [0·56-1·46]) and was determined to be of low quality.(111) Furthermore, women are screened for chlamydia by presence of symptoms rather than a test and many cases of chlamydia are asymptomatic (up to 75% of cases in women are asymptomatic).(134) Even if women are correctly screened and identified, the proper course of antibiotics may not be administered. Given these limitations, we will not be correcting for treatment for the prevalence of chlamydia.

Asymptomatic bacteriuria

Prevalence: Country specific estimates of prevalence and corresponding 95% confidence intervals of UTI in women of reproductive age from IHME were used in the model.(135)

Quality: Medium. Modeled country-specific data and WRA, not specifically pregnant women.

Note: Other studies and papers on the topic have quoted a prevalence rate of asymptomatic bacteriuria in pregnancy ranging from 2-10%.(113),(115),(136) This is higher than the estimates reported by IHME, but these estimates are also from specific study populations and therefore may be biased upward.

Treatment: We will not be adjusting for treatment due to lack of significant effect and coverage.

Periodontal disease

Prevalence: The prevalence data and 95% CIs for periodontal disease is from the IHME database for women of reproductive age. The mean value across the countries is 8·93%, with a median value equal to 9·35%, 25th percentile value of 6·34%, and a 75th percentile value of 11·37%.(135)

Quality: Medium. Modeled country-specific data and WRA, not specifically pregnant women.

Treatment adjustment: We are assuming no coverage of treatment and therefore will not be correcting for treatment in the prevalence data.

Bacterial Vaginosis

Prevalence: The prevalence of bacterial vaginosis from countries in the WHO classified “African” region is from a meta-analysis of 18 studies from Sub-Saharan Africa published in 2018. This prevalence data reflects the BV prevalence in south/eastern African countries and was

reported separately for women 15-24 (35·2% [27·7-43·6%]) and in women ages 25-49 years old (33·3% [24·7-43·1%]). The estimates were combined by weighting the total sample size for the two analyses to get an "average" prevalence estimate of 34·1% (25·9-43·3%).[137]

The prevalence for the remaining countries, including those in the South Asia regional group, is from a systematic review from 2019, using the average prevalence of bacterial vaginosis in women of reproductive age for each region estimated using a random effects model. While this is not ideal, there is a paucity of BV prevalence data. The regional 95% confidence intervals are included.[138]

Quality: Medium, overall. High for the country-specific, primarily collected data from the meta-analysis of sub-Saharan African studies. Low for the regional estimates for the remaining countries in the model.

Treatment adjustment: Due to the lack of consistent evidence on the efficacy of treatment of bacterial vaginosis on preterm birth, we will not be correcting for treatment in the prevalence estimates. Furthermore, similar to chlamydia, screening and treatment for bacterial vaginosis is currently based on symptoms and many cases are asymptomatic.

Group B Strep

Prevalence: The prevalence and 95% CIs for group B strep in women of reproductive age is regional data calculated by a 2016 meta-analysis of 78 studies.[139]

Quality: Low. Only regional data available.

Treatment: The majority of recent treatment studies published investigate the use of intrapartum antibiotics to prevent colonization in the infant. Both ACOG and the WHO recommend intrapartum treatment of GBS with antibiotics in order to prevent vertical transmission.[124],[125] However, WHO states that more research is needed to determine the impact of GBS screening and treatment on preterm birth. As the prevalence of the practice of screening and treatment for GBS during antenatal care in these settings is unknown (though, likely low) and efficacy of this practice is also unclear, we will not be correcting for treatment.

Zinc deficiency

Prevalence: The estimates of the prevalence and 95% CIs of zinc deficiency are from the 2012 paper which estimated inadequate zinc intake prevalence from national food balance data from the Food and Agriculture Association from the United Nations. This estimate is for each country as a whole and is not specific to pregnant women.[140]

Quality: Low. Although it is country-specific data, it is not specific to women of reproductive age or pregnant women.

Calcium deficiency

Prevalence: The prevalence estimate is from a 2017 analysis that used national food balance sheet data from the UN Food and Agriculture Organization, UN population data, food composition tables and nutrient intake and requirements data to estimate low intake prevalence for 14 micronutrients, including calcium.(141) The years collected spanned 1961 to 2011 and for each country the prevalence of inadequate intake was estimated fusing the population-weighted estimate average requirements and the micronutrient availability distribution. These were country-specific estimates, but not specific to women of reproduction age or pregnant women

One of the difficulties is the lack of clear guidance on the recommended daily intake of calcium during pregnancy. While recommendations vary, the WHO recommends daily calcium supplementation of 1.5-2 g in populations with low dietary calcium intake.[(124)] Recently with the impact of calcium supplementation becoming clearer, there has been work done in estimating calcium intake among adults and women in low- and middle-income countries.[(142),(143)] Both of these papers ran systematic reviews on daily calcium intake. Both papers found that the average calcium intake for adults and for women was well under 1000 mg per day. In fact, the more recent work by Cormick et al. clearly called for the need for calcium supplementation in all pregnant women in LMICs.

Quality: Low. Although it is country-specific data, it is not specific to women of reproductive age or pregnant women.

Pre-eclampsia

Prevalence: The prevalence data are from a 74 study analysis, which reported regional level data for preeclampsia. There were two values reported, a crude pooled preeclampsia incidence estimate from the available study data and a modeled incidence estimate that populated data for countries without crude data using “country macroeconomic indicators” that includes a 95% confidence interval. For four of the six WHO regions, the modeled estimate was larger (in some cases, nearly two times as larger) compared to the crude incidence. Due to this difference, the crude data is currently in the model to avoid overestimation.[(144)]

Quality: Low. Only regional level data available.

Maternal anemia

Prevalence: The prevalence of maternal anemia is from the WHO estimates from 2013, which compiled data on hemoglobin concentration in pregnant women from over 250 population-based data sources in 107 different countries.[(145)]

Quality: High. Country-specific, primarily collected data from population-representative surveys.

Chronic hypertension

Prevalence: The prevalence of chronic hypertension is from a 2016 analysis of data from 90 studies, which reported the regional prevalence for women ages 20-29, 30-39, and 40-49.[(146)] To

calculate the prevalence of chronic hypertension in pregnancy, these prevalence rates were weighted by the fertility rates for the age cohorts above. To do this, the proportion of the age cohort out of the total TFR_{20-49} was multiplied by the prevalence data and then these estimates were added together for an overall prevalence estimate for the region. The fertility rate data by region is from the United Nations world population prospects 2017.[(147)] 95% confidence intervals were not given for the estimates, therefore a confidence interval with a width of 10% was constructed around the point estimate.

Quality: Low. Only regional data available.

Treatment: From LiST, the effectiveness value of case management of hypertensive disorders is equal to 0.500. However, the Cochrane review of 15 randomized controlled trials comparing any antihypertensive drug compared to no drug/placebo and demonstrated an inconclusive effect (RR=0·96 [0·83-1·12]).[(62)] After consideration, we will not be adjusting for treatment effect in the prevalence estimate.

Gestational Diabetes

Prevalence: The prevalence data and 95% CIs for gestational diabetes is by WHO region from a meta-analysis published in 2016. Depending on the region from which the individual study was done or the year the study was conducted there was some heterogeneity in diagnostic criteria used to define GDM. The average prevalence across the regional data for all the countries was 9·07%, with a median of 8·9%, 25th percentile equal to 5·8%, and a 75th percentile equal to 11·7%.[(148)]

Quality: Low. Only regional level estimates available

Treatment: Cochrane reviews on various treatments (insulin, lifestyle alterations like diet and exercise, other anti-diabetic medication) state that there is inconclusive evidence on treatment effect on preterm birth.[(99),(100)] Individual studies found no difference in gestational age at birth between treatment and intervention groups.[(101),(102)] Therefore, we will not adjust for treatment.

Short birth interval

Prevalence: The country specific estimates for prevalence of short birth interval (<18 months) is from the Lives Save Tool (LiST) database, which has compiled data from multiple surveys (DHS, MICS, FFS and national surveys). 95% confidence intervals were not included in these estimates, therefore a confidence interval with a width of 10% was constructed around the point estimate.

Quality: High. Country-specific estimates available.

Multiple gestations

Prevalence: The prevalence is estimated from a 2011 paper that used DHS data to estimate regional twinning rates per 1000 births (calculated in the paper by dividing the number of twin births by the total number of births multiplied by 1000, so to get the percent births that were twins this rate was divided by 10), with a range of 1·1-3% of all births being twins.[(149)] 95% confidence intervals were not given for the estimates, therefore a confidence interval with a width of 10% was constructed around the point estimate. Estimates from the CDC are in agreement with the above data (estimating 2-3% of births are twin births).[(150)]

Quality: Low. Only regional level data available.

Short maternal height

Prevalence: The estimates for short maternal height (<145 cm) prevalence are from the DHS surveys. For countries with a DHS survey between 2006-2016, country-specific estimates are used. For countries without a recent DHS, a regional estimate was calculated using the average of the 2006-2016 survey data from country estimates in the region. 95% confidence intervals were not given for the estimates, therefore a confidence interval with a width of 10% was constructed around the point estimate.[(151)]

Quality: Medium overall. High for countries with country-specific data, low for those with regional estimates.

Maternal age & parity

Prevalence: The prevalence for the maternal age and parity categories comes from the Family Planning module of the LiST data base, which compiles data from national surveys, demographic health surveys, multiple indicator cluster surveys and more.

Quality: High. Country specific estimates from population-representative surveys.

Tobacco smoking

Prevalence: A meta-analysis of 295 studies on prevalence of smoking during pregnancy that was published in 2018 was used for regional prevalence estimates and the corresponding 95% confidence intervals. The regional estimates were calculated from the country specific estimates in the meta-analysis, weighted by number of live births in the country for the most recently available year.[(152)]

Quality: Low. Only regional data available.

Low BMI

Prevalence: The estimates for prevalence of lower maternal BMI (<18·5) are from the LiST database, with the majority of data from 2014 and the remaining from 2013. Many of these data sources are national surveys such as DHS and MICS. 95% confidence intervals were not given

for the estimates, therefore a confidence interval with a width of 10% was constructed around the point estimate.

Quality: High. Country-specific estimates available for most countries.

Cervical insufficiency

Prevalence: In most medical texts and summaries, the prevalence of cervical insufficiency (CI) (measured by cervical length ≤ 25 mm) is said to be 1% of pregnant women or lower.(153, 154) We did a search for prevalence of CI as measured by length of cervix around 26 weeks and found three studies in low- and middle-income countries. The two studies in Nigeria found prevalence of 1·4% and 0·85% while the study in China found a prevalence of 0·45%.(155-157) These values fit well with the data from higher income countries. For this analysis we will use a prevalence value of 0·75%.

Quality: Low. Global prevalence only.

Ambient air particulate matter

Prevalence: The prevalence estimates for ambient air particulate matter ($PM_{2.5}$) are from an analysis for the Global Burden of Disease study 2015. The authors estimated the “summary exposure value” SEV, which was age standardized and estimated for women specifically in every country. The authors state that the SEV represents the relative-risk weight prevalence of the exposure. Furthermore, the exposure estimate for $PM_{2.5}$ had a “data representative index” (DRI) of 100% because there were data points on the exposure for all countries and all years. The estimates for exposure to ambient air pollution were calculated using the WHO air pollution data base and satellite based estimates.(158)

Quality: Medium. Modeled country-specific data.

Indoor air pollution

Prevalence: The prevalence estimates for the indoor air pollution combine three sources: the percent of the population with access to clean fuels and technologies for cooking,(159) the deaths from household pollution from solid fuels,(135) and the total population in 2020.(147) The total population was adjusted to reflect the deaths from household air pollution and the proportion of population with access to clean fuels was subtracted from this percentage (representing the proportion of the population alive and able to be exposed) in order to estimate the proportion of the population without access to clean fuel options. This best reflects the risk estimate exposure definition, which is use of solid fuels. This estimate is for the entire population and is not representative of pregnant women or women of reproductive age.

Quality. Low. Although it is country-specific data, it is not specific to women of reproductive age or pregnant women.

Intimate partner violence during pregnancy

Prevalence: The prevalence data is primarily from the Demographic Health Survey, which collects data on whether a woman experienced violence during pregnancy.[(151)] For those countries that do not have a recent DHS, but have a prevalence estimate from one of the two reports below, this data point was used. For those that have neither, regional estimates using the individual data points were calculated.

There are two other studies the prevalence of IPV during pregnancy. One meta-analysis reports the prevalence from 19 countries using DHS and IVAWS data. The prevalence in the low and middle income countries ranged from 2·0% to 13·5%.[(160)] The second study is from the WHO multi-country study on women's health and domestic violence against women, which reported on abused during pregnancy in 10 countries. Within certain countries there was a rural and urban study site. The prevalence range in this analysis went from a minimum of 3·8% to a maximum of 27·6%.[(161)]

The WHO published a report on global and regional estimates of violence against women, which compiled data from 79 countries and two territories.[(162)] All the individual prevalence estimates were from primary data from population surveys on women ages 15-49, including the WHO's own survey, Demographic Health Surveys, International Violence Against Women Surveys, and many more. They reported regional lifetime prevalence of physical and/or sexual intimate partner violence, which ranged from 24·6% in the Western Pacific region to 37·7% in the South-East Asian region. However, this is not specific to pregnant women.

Quality: Medium overall. High for countries with country-specific data, low for those with regional estimates

4. Search Strategies

Search strategy 1: results on pubmed- 105773(tiab)
“Premature birth”[Mesh] OR “birth, premature”[tiab] OR “births, premature”[tiab] OR “Premature Births”[tiab] OR “Preterm Birth”[tiab] OR “Birth, Preterm”[tiab] OR “Births, Preterm”[tiab] OR “Preterm Births”[tiab] OR ((“Preterm”[tiab] OR “Premature”[tiab]) AND (“Birth”[tiab] OR “births”[tiab] OR “delivery”[tiab])) OR “Infant, Premature”[Mesh] OR “infants, premature”[tiab] OR “premature infant”[tiab] OR “Preterm infants”[tiab] OR “Infants, preterm”[tiab] OR “Preterm Infant”[tiab] OR “Premature infants”[tiab] OR “Neonatal Prematurity”[tiab] OR “Prematurity, Neonatal”[tiab]
AND
Search strategy 2: results on pubmed- 2090865
“Risk Factors”[Mesh] OR “Factor, risk”[tiab] OR “Factors, risk”[tiab] OR “Risk factor”[tiab] OR “Causality”[Mesh] OR “cause”[tiab] OR “causes”[tiab]

Search strategy 3 (above combine): results on pubmed- 21570
Narrow search to reviews only- 3,783

Search strategy 4 results on pubmed- 1758

“Population attributable fraction” [tw]

search strategy 5 (1 and 4 combined): results on pubmed: 24

Table 4. Country Estimates									
	Afghanistan	Algeria	Angola	Azerbaijan	Bangladesh	Benin	Bhutan	Bolivia	Botswana
Short birth interval	1.99%	1.83%	1.89%	3.34%	1.32%	0.76%	2.00%	1.66%	1.97%
Young maternal age (< 18 years) & primiparity	1.04%	1.57%	2.20%	0.62%	3.65%	0.88%	1.96%	1.85%	1.93%
Maternal age 18-35 years & parity >3	4.81%	2.24%	4.09%	2.12%	2.64%	3.97%	1.45%	3.55%	1.42%
Older maternal age (>35 years) & parity >3	2.83%	2.76%	2.98%	1.11%	0.74%	2.40%	1.24%	3.27%	1.22%
Maternal Anemia	13.28%	10.71%	13.04%	11.24%	13.67%	15.05%	14.25%	12.16%	10.38%
Short height (<145 cm)	1.08%	0.49%	0.44%	0.20%	2.95%	0.36%	2.68%	2.41%	0.47%
Low BMI (<18.5 kg/m^2)	3.46%	1.25%	2.33%	0.90%	4.36%	1.80%	2.50%	0.53%	1.75%
Chronic hypertension	3.49%	7.32%	6.45%	5.09%	3.73%	6.05%	4.03%	4.98%	7.01%
Twin pregnancy	2.32%	2.97%	2.62%	5.14%	1.66%	2.46%	1.80%	1.78%	2.85%
Fetal gender (male)	1.77%	1.90%	1.68%	2.03%	1.71%	1.57%	1.85%	1.83%	1.82%
Ambient air pollution	3.43%	2.99%	2.65%	2.96%	4.51%	2.49%	3.85%	2.65%	1.91%
Indoor air pollution	10.21%	1.29%	7.36%	0.82%	11.17%	11.56%	6.63%	6.08%	5.37%
Intimate partner violence	4.04%	3.61%	3.18%	2.51%	2.39%	2.98%	2.59%	4.30%	3.46%
Tobacco smoking	0.16%	0.14%	0.26%	1.47%	0.19%	0.03%	0.20%	1.40%	0.13%
Short cervical length (<25mm)	2.27%	2.45%	2.16%	2.62%	2.20%	2.02%	2.38%	2.35%	2.34%
Gestational diabetes	3.12%	2.35%	2.07%	1.66%	2.75%	1.94%	2.98%	2.31%	2.25%
Pre-eclampsia	0.29%	1.03%	0.91%	1.05%	0.63%	0.85%	0.68%	0.57%	0.99%
HIV	0.03%	0.02%	0.49%	0.02%	0.00%	0.31%	0.03%	0.04%	6.27%
HIV	0.02%	0.00%	3.51%	0.00%	0.00%	7.22%	0.00%	0.00%	0.03%
Malaria	0.65%	1.73%	1.95%	0.25%	0.44%	1.90%	0.35%	0.55%	2.18%
Syphilis	0.55%	0.62%	0.55%	0.40%	0.27%	0.51%	0.30%	1.22%	0.60%
Chlamydia	0.06%	0.08%	0.05%	0.17%	0.07%	0.07%	0.08%	0.14%	0.06%
Asymptomatic bacteriuria	1.99%	3.62%	1.70%	3.28%	3.76%	3.00%	3.76%	3.68%	0.70%
Periodontal Infection	8.39%	11.70%	10.31%	8.89%	9.11%	9.67%	9.86%	8.42%	11.21%
Total	71.27%	64.68%	74.84%	57.88%	73.91%	79.86%	67.48%	67.72%	68.32%

	Burkina Faso	Burundi	Cambodia	Cameroon	Central African Republic	Chad	Comoros	Congo	Democratic Republic of the Congo
Short birth interval	0.70%	1.61%	1.16%	1.67%	1.96%	1.61%	4.06%	1.45%	2.18%
Young maternal age (< 18 years) & primiparity	1.10%	0.49%	0.76%	1.81%	2.05%	1.78%	1.16%	2.02%	1.26%
Maternal age 18-35 years & parity >3	4.04%	4.08%	2.50%	4.00%	4.07%	4.85%	4.04%	3.48%	4.09%
Older maternal age (>35 years) & parity >3	3.07%	3.55%	1.88%	2.27%	2.19%	2.48%	3.12%	2.57%	2.92%
Maternal Anemia	14.24%	8.65%	15.41%	12.89%	12.74%	13.09%	10.27%	14.98%	12.60%
Short height (<145 cm)	0.13%	1.12%	1.41%	0.21%	0.40%	0.14%	0.63%	0.35%	1.11%
Low BMI (<18.5 kg/m^2)	2.41%	2.42%	3.09%	1.38%	2.47%	2.60%	1.91%	2.28%	2.52%
Chronic hypertension	5.99%	6.37%	3.68%	6.13%	5.95%	6.11%	6.51%	6.22%	6.04%
Twin pregnancy	2.44%	2.59%	1.80%	2.49%	2.42%	2.48%	2.65%	2.53%	2.46%
Fetal gender (male)	1.56%	1.66%	1.85%	1.59%	1.55%	1.59%	1.69%	1.62%	1.57%
Ambient air pollution	2.66%	3.01%	2.62%	3.65%	2.84%	2.89%	1.80%	3.13%	2.88%
Indoor air pollution	11.20%	12.69%	12.41%	10.22%	11.82%	12.07%	12.20%	9.69%	11.76%
Intimate partner violence	2.96%	3.14%	1.89%	3.02%	2.93%	3.01%	3.21%	3.07%	2.98%
Tobacco smoking	0.01%	0.14%	0.39%	0.03%	0.11%	0.12%	0.25%	0.07%	0.11%
Short cervical length (<25mm)	2.00%	2.13%	2.38%	2.05%	1.99%	2.04%	2.18%	2.08%	2.02%
Gestational diabetes	1.93%	2.05%	2.98%	1.97%	1.91%	1.96%	2.09%	2.00%	1.94%
Pre-eclampsia	0.84%	0.90%	1.05%	0.86%	0.84%	0.86%	0.92%	0.87%	0.85%
HIV	0.20%	0.30%	0.07%	0.94%	0.81%	0.31%	0.03%	0.94%	0.18%
HIV	7.89%	4.30%	0.20%	6.17%	7.36%	4.00%	0.10%	4.08%	6.17%
Malaria	1.87%	1.66%	0.32%	1.76%	1.96%	1.95%	2.05%	1.83%	1.78%
Syphilis	0.51%	0.54%	1.01%	0.52%	0.51%	0.52%	0.55%	0.53%	0.51%
Chlamydia	0.06%	0.04%	0.05%	0.07%	0.04%	0.06%	0.05%	0.05%	0.04%
Asymptomatic bacteriuria	2.80%	2.09%	1.07%	2.94%	1.93%	2.54%	2.53%	1.97%	1.72%
Periodontal Infection	9.58%	10.19%	8.52%	9.79%	9.51%	9.77%	10.41%	9.95%	9.66%
Total	80.20%	75.71%	68.47%	78.43%	80.35%	78.83%	74.40%	77.75%	79.36%

	Côte d'Ivoire	Djibouti	Dominican Republic	Equatorial Guinea	Eritrea	Ethiopia	Gabon	Gambia	Ghana
Short birth interval	1.20%	1.98%	2.41%	1.45%	2.15%	1.93%	1.81%	1.97%	0.78%

Young maternal age (< 18 years) & primiparity	1.81%	1.94%	2.85%	1.59%	1.51%	1.08%	2.18%	1.07%	0.97%
Maternal age 18-35 years & parity >3	3.88%	1.43%	3.07%	3.08%	2.92%	4.64%	3.50%	3.93%	3.24%
Older maternal age (>35 years) & parity >3	2.66%	1.23%	1.37%	2.71%	3.19%	3.17%	2.63%	2.89%	3.56%
Maternal Anemia	14.85%	10.29%	11.00%	13.78%	10.97%	7.12%	16.04%	14.11%	15.55%
Short height (<145 cm)	0.23%	1.12%	0.42%	0.42%	0.45%	0.59%	0.24%	0.05%	0.18%
Low BMI (<18.5 kg/m^2)	1.64%	2.50%	1.20%	1.74%	3.59%	3.14%	1.61%	2.16%	1.44%
Chronic hypertension	6.29%	3.63%	5.29%	6.20%	6.72%	6.60%	6.51%	6.16%	6.17%
Twin pregnancy	2.56%	2.41%	1.89%	2.52%	2.73%	2.68%	2.64%	2.50%	2.51%
Fetal gender (male)	1.64%	1.83%	1.94%	1.61%	1.75%	1.71%	1.69%	1.60%	1.61%
Ambient air pollution	2.04%	3.49%	2.27%	2.87%	3.10%	2.74%	2.82%	3.18%	2.06%
Indoor air pollution	10.87%	12.90%	1.23%	8.69%	7.56%	13.15%	3.58%	12.60%	10.37%
Intimate partner violence	3.10%	4.20%	4.57%	3.06%	3.31%	3.25%	3.21%	3.04%	3.05%
Tobacco smoking	0.12%	0.15%	0.68%	0.12%	0.13%	0.09%	0.44%	0.03%	0.01%
Short cervical length (<25mm)	2.10%	2.36%	2.50%	2.07%	2.25%	2.21%	2.18%	2.06%	2.06%
Gestational diabetes	2.02%	3.24%	2.46%	1.99%	2.16%	2.12%	2.09%	1.98%	1.99%
Pre-eclampsia	0.88%	0.30%	0.61%	0.87%	0.94%	0.93%	0.91%	0.87%	0.87%
HIV	0.79%	0.34%	0.12%	1.49%	0.10%	0.23%	1.35%	0.37%	0.55%
HIV	3.12%	0.00%	0.00%	6.82%	0.40%	0.48%	3.89%	1.28%	5.63%
Malaria	1.78%	0.49%	0.58%	1.93%	2.00%	1.93%	2.03%	1.69%	1.93%
Syphilis	0.53%	0.57%	1.29%	0.53%	0.57%	0.56%	0.55%	0.52%	0.53%
Chlamydia	0.07%	0.05%	0.10%	0.05%	0.05%	0.05%	0.05%	0.06%	0.06%
Asymptomatic bacteriuria	2.95%	3.02%	5.32%	2.18%	2.38%	2.42%	2.33%	4.65%	3.52%
Periodontal Infection	10.06%	8.72%	8.95%	9.92%	10.74%	10.55%	10.40%	9.85%	9.87%
Total	77.20%	68.19%	62.15%	77.68%	71.66%	73.36%	74.72%	78.61%	78.51%

	Guatemala	Guinea	Guinea-Bissau	Guyana	Haiti	Honduras	India	Indonesia	Iraq
Short birth interval	2.76%	0.84%	1.91%	1.93%	1.61%	1.68%	2.27%	1.25%	2.29%
Young maternal age (< 18 years) & primiparity	2.30%	2.33%	2.59%	2.27%	1.32%	2.88%	0.77%	0.71%	3.10%
Maternal age 18-35 years & parity >3	3.35%	3.83%	2.93%	3.46%	2.94%	3.13%	2.56%	1.96%	3.50%

Older maternal age (>35 years) & parity >3	2.27%	2.85%	2.10%	2.12%	3.47%	2.08%	0.72%	2.94%	2.51%
Maternal Anemia	9.74%	14.78%	13.22%	10.18%	13.76%	7.72%	15.16%	10.02%	10.84%
Short height (<145 cm)	5.89%	0.23%	0.43%	0.89%	0.23%	2.15%	2.66%	2.75%	1.19%
Low BMI (<18.5 kg/m^2)	0.73%	1.27%	2.17%	1.20%	1.90%	0.98%	4.49%	2.67%	1.11%
Chronic hypertension	4.87%	6.04%	6.30%	5.16%	4.75%	5.13%	3.82%	4.13%	3.87%
Twin pregnancy	1.74%	2.46%	2.56%	1.84%	1.70%	1.83%	1.71%	1.84%	2.57%
Fetal gender (male)	1.79%	1.57%	1.64%	1.89%	1.74%	1.88%	1.75%	1.90%	1.96%
Ambient air pollution	2.94%	1.92%	2.46%	1.95%	2.33%	3.21%	4.02%	1.88%	3.87%
Indoor air pollution	8.41%	11.63%	12.43%	4.46%	13.24%	8.02%	8.89%	7.14%	0.58%
Intimate partner violence	4.20%	2.98%	3.11%	4.45%	4.09%	4.42%	2.45%	2.65%	4.48%
Tobacco smoking	0.24%	0.11%	0.12%	0.55%	0.27%	0.29%	0.02%	0.40%	0.16%
Short cervical length (<25mm)	2.30%	2.02%	2.11%	2.44%	2.24%	2.42%	2.26%	2.44%	2.52%
Gestational diabetes	2.26%	1.94%	2.03%	2.39%	2.20%	2.38%	2.82%	3.05%	3.45%
Pre-eclampsia	0.56%	0.85%	0.89%	0.59%	0.55%	0.59%	0.64%	0.70%	0.32%
HIV	0.08%	0.36%	0.87%	0.42%	0.70%	0.05%	0.03%	0.08%	0.03%
HIV	0.00%	6.71%	1.31%	0.38%	0.07%	0.00%	0.11%	0.10%	0.00%
Malaria	0.54%	1.84%	1.82%	0.52%	0.50%	0.56%	0.47%	0.53%	0.62%
Syphilis	1.19%	0.51%	0.54%	1.26%	1.16%	1.25%	0.28%	0.30%	0.61%
Chlamydia	0.05%	0.06%	0.07%	0.11%	0.09%	0.06%	0.08%	0.05%	0.08%
Asymptomatic bacteriuria	3.01%	3.01%	3.24%	5.11%	3.91%	3.32%	4.91%	5.00%	2.51%
Periodontal Infection	8.23%	9.66%	10.08%	8.72%	8.03%	8.67%	9.34%	10.10%	9.30%
Total	69.45%	79.81%	76.93%	64.30%	72.80%	64.70%	72.22%	64.58%	61.49%

	Jamaica	Kenya	North Korea	Kyrgyzstan	Lao People's Democratic Republic	Lesotho	Liberia	Madagascar	Malawi
Short birth interval	2.11%	1.94%	1.93%	1.92%	1.94%	0.85%	1.50%	1.70%	1.06%
Young maternal age (< 18 years) & primiparity	2.07%	1.36%	1.89%	0.23%	1.66%	1.90%	2.17%	2.20%	1.92%
Maternal age 18-35 years & parity >3	1.52%	3.67%	1.39%	3.34%	2.38%	2.50%	3.62%	3.94%	3.78%
Older maternal age (>35 years) & parity >3	1.31%	2.39%	1.20%	2.43%	2.92%	2.18%	2.84%	3.18%	2.39%

Maternal Anemia	10.49%	10.37%	17.20%	10.79%	12.79%	9.83%	13.70%	9.66%	10.45%
Short height (<145 cm)	2.10%	0.19%	2.59%	0.22%	1.53%	0.36%	0.51%	1.69%	0.65%
Low BMI (<18.5 kg/m^2)	1.03%	2.02%	2.09%	1.24%	3.05%	1.53%	1.80%	2.97%	1.99%
Chronic hypertension	5.32%	6.48%	3.89%	5.00%	4.00%	6.87%	6.21%	6.45%	6.31%
Twin pregnancy	1.90%	2.63%	1.74%	5.05%	1.96%	2.79%	2.53%	2.62%	2.56%
Fetal gender (male)	1.95%	1.68%	1.79%	2.00%	2.01%	1.79%	1.62%	1.68%	1.64%
Ambient air pollution	2.10%	1.75%	2.76%	2.08%	3.08%	2.20%	0.88%	1.92%	2.16%
Indoor air pollution	2.08%	11.73%	12.86%	3.98%	0.91%	8.81%	12.40%	12.92%	11.90%
Intimate partner violence	4.59%	3.20%	1.83%	2.46%	2.06%	3.39%	3.07%	3.18%	3.11%
Tobacco smoking	1.03%	0.06%	0.19%	1.44%	0.22%	0.05%	0.04%	0.11%	0.09%
Short cervical length (<25mm)	2.51%	2.17%	2.30%	2.57%	2.59%	2.30%	2.08%	2.16%	2.11%
Gestational diabetes	2.47%	2.08%	2.87%	1.63%	3.24%	2.21%	2.00%	2.07%	2.03%
Pre-eclampsia	0.61%	0.91%	0.65%	1.03%	1.14%	0.97%	0.87%	0.91%	0.89%
HIV	0.03%	1.27%	0.03%	0.03%	0.07%	5.34%	0.31%	0.03%	2.13%
HIV	0.00%	1.66%	0.00%	0.00%	0.04%	0.00%	4.18%	2.01%	4.98%
Malaria	0.41%	1.64%	0.49%	0.25%	0.34%	1.95%	1.98%	2.06%	1.64%
Syphilis	1.30%	0.55%	0.29%	0.39%	1.10%	0.58%	0.53%	0.55%	0.54%
Chlamydia	0.10%	0.05%	0.04%	0.19%	0.05%	0.06%	0.07%	0.05%	0.04%
Asymptomatic bacteriuria	5.60%	4.45%	2.45%	2.49%	0.73%	0.60%	3.14%	0.67%	1.97%
Periodontal Infection	8.99%	10.36%	9.51%	8.73%	9.27%	10.99%	9.94%	10.31%	10.08%
Total	61.64%	74.64%	71.98%	59.50%	59.09%	70.04%	77.96%	75.04%	76.42%

	Mali	Mauritania	Morocco	Mozambique	Myanmar	Namibia	Nepal	Nicaragua	Niger
Short birth interval	1.39%	2.10%	1.71%	1.04%	2.22%	1.02%	1.42%	2.41%	1.25%
Young maternal age (< 18 years) & primiparity	1.68%	1.47%	0.77%	2.11%	0.61%	1.55%	2.00%	3.09%	1.40%
Maternal age 18-35 years & parity >3	4.27%	2.84%	2.13%	2.75%	2.58%	2.73%	2.77%	3.85%	4.69%
Older maternal age (>35 years) & parity >3	2.43%	3.11%	3.30%	2.52%	3.23%	2.94%	0.78%	2.28%	2.75%
Maternal Anemia	14.56%	12.62%	11.47%	12.58%	10.62%	10.61%	13.24%	7.27%	13.92%
Short height (<145 cm)	0.11%	0.44%	1.21%	0.94%	1.63%	0.13%	2.61%	2.00%	0.18%
Low BMI (<18.5 kg/m^2)	2.10%	2.03%	1.12%	2.09%	2.83%	2.05%	3.41%	0.88%	2.43%
Chronic hypertension	5.93%	6.55%	3.93%	6.08%	3.94%	6.81%	3.89%	5.05%	5.95%

Twin pregnancy	2.41%	2.66%	2.60%	2.47%	1.76%	2.77%	1.74%	1.80%	2.42%
Fetal gender (male)	1.54%	1.70%	1.98%	1.58%	1.81%	1.77%	1.79%	1.85%	1.55%
Ambient air pollution	2.76%	4.18%	2.55%	1.77%	3.79%	2.09%	4.28%	2.54%	3.35%
Indoor air pollution	11.83%	7.37%	0.33%	11.56%	12.04%	7.97%	10.25%	8.26%	11.78%
Intimate partner violence	2.92%	3.23%	4.54%	3.00%	2.53%	3.36%	2.50%	4.35%	2.94%
Tobacco smoking	0.03%	0.12%	0.16%	0.20%	0.28%	0.67%	0.88%	0.87%	0.11%
Short cervical length (<25mm)	1.98%	2.19%	2.55%	2.03%	2.33%	2.28%	2.30%	2.38%	1.99%
Gestational diabetes	1.91%	2.11%	3.50%	1.95%	2.91%	2.19%	2.87%	2.34%	1.91%
Pre-eclampsia	0.83%	0.92%	0.33%	0.85%	0.66%	0.96%	0.66%	0.58%	0.84%
HIV	0.25%	0.06%	0.02%	2.79%	0.18%	3.81%	0.02%	0.03%	0.06%
HIV	7.43%	1.30%	0.00%	6.72%	0.04%	0.88%	0.00%	0.01%	6.95%
Malaria	1.81%	2.01%	0.74%	1.54%	0.47%	1.93%	0.49%	0.55%	1.83%
Syphilis	0.50%	0.56%	0.61%	0.52%	0.29%	0.58%	0.29%	1.23%	0.51%
Chlamydia	0.06%	0.08%	0.08%	0.04%	0.06%	0.06%	0.06%	0.05%	0.06%
Asymptomatic bacteriuria	2.65%	3.57%	5.41%	2.12%	2.87%	0.61%	2.69%	3.79%	2.14%
Periodontal Infection	9.48%	10.48%	9.43%	9.72%	9.63%	10.89%	9.52%	8.54%	9.52%
Total	80.88%	73.70%	60.48%	78.99%	69.31%	70.64%	70.45%	66.03%	80.53%

	Nigeria	Pakistan	Panama	Papua New Guinea	Paraguay	Philippines	Rwanda	Senegal	Sierra Leone
Short birth interval	1.64%	3.60%	2.00%	3.44%	2.02%	2.98%	1.48%	1.40%	1.21%
Young maternal age (< 18 years) & primiparity	1.35%	0.59%	1.96%	0.35%	1.27%	1.12%	0.48%	1.14%	1.80%
Maternal age 18-35 years & parity >3	4.13%	4.35%	1.44%	2.55%	4.10%	3.04%	2.92%	3.77%	3.86%
Older maternal age (>35 years) & parity >3	2.99%	2.26%	1.24%	3.47%	3.66%	3.20%	3.21%	3.36%	2.92%
Maternal Anemia	14.52%	14.55%	9.29%	11.77%	10.63%	10.65%	5.99%	16.17%	11.81%
Short height (<145 cm)	0.43%	1.15%	1.99%	1.40%	2.01%	1.44%	0.68%	0.05%	0.47%
Low BMI (<18.5 kg/m^2)	1.86%	3.09%	0.74%	0.99%	0.81%	3.06%	1.68%	2.38%	1.97%
Chronic hypertension	6.13%	3.43%	5.03%	3.66%	5.09%	3.76%	6.31%	6.36%	6.06%
Twin pregnancy	2.49%	2.28%	1.80%	1.79%	1.82%	1.84%	2.57%	2.59%	2.46%
Fetal gender (male)	1.59%	1.73%	1.85%	1.84%	1.87%	1.89%	1.64%	1.65%	1.57%
Ambient air pollution	2.74%	3.93%	1.66%	1.49%	1.82%	2.47%	3.15%	2.82%	1.67%

Indoor air pollution	11.89%	9.16%	13.37%	13.06%	5.86%	9.25%	12.59%	9.04%	12.10%
Intimate partner violence	3.02%	3.97%	4.34%	1.88%	4.39%	1.94%	3.11%	3.14%	2.99%
Tobacco smoking	0.03%	0.20%	0.98%	0.20%	0.99%	0.85%	0.06%	0.11%	0.62%
Short cervical length (<25mm)	2.05%	2.23%	2.38%	2.37%	2.41%	2.44%	2.11%	2.13%	2.03%
Gestational diabetes	1.97%	3.06%	2.34%	2.96%	2.36%	3.04%	2.03%	2.05%	1.95%
Pre-eclampsia	0.86%	0.28%	0.58%	1.04%	0.59%	1.07%	0.89%	0.89%	0.85%
HIV	0.60%	0.02%	0.14%	0.24%	0.08%	0.01%	0.64%	0.12%	0.43%
HIV	5.78%	0.03%	0.00%	3.45%	0.00%	0.00%	9.91%	1.46%	7.47%
Malaria	1.66%	0.63%	0.39%	0.29%	0.56%	0.32%	1.92%	2.02%	1.78%
Syphilis	0.52%	0.54%	1.23%	1.00%	1.24%	1.03%	0.54%	0.54%	0.52%
Chlamydia	0.06%	0.07%	0.06%	0.06%	0.17%	0.08%	0.05%	0.07%	0.07%
Asymptomatic bacteriuria	0.80%	3.34%	4.23%	0.34%	3.18%	0.58%	2.41%	3.15%	2.95%
Periodontal Infection	9.80%	8.24%	8.51%	8.48%	8.61%	8.72%	10.10%	10.18%	9.68%
Total	78.93%	72.74%	67.54%	68.14%	65.55%	64.80%	76.46%	76.58%	79.24%

	Solomon Islands	Somalia	South Africa	South Sudan	Sudan	Suriname	Swaziland	Tajikistan	United Republic of Tanzania
Short birth interval	3.51%	0.79%	1.14%	2.01%	2.22%	2.10%	0.99%	1.93%	0.99%
Young maternal age (< 18 years) & primiparity	0.36%	1.25%	1.86%	1.25%	1.14%	2.06%	2.60%	0.25%	1.54%
Maternal age 18-35 years & parity >3	2.61%	3.27%	1.59%	3.58%	5.12%	1.52%	3.37%	3.69%	3.64%
Older maternal age (>35 years) & parity >3	3.55%	3.40%	2.78%	3.03%	3.06%	1.31%	2.50%	1.92%	3.36%
Maternal Anemia	10.91%	13.45%	10.00%	10.65%	10.78%	10.65%	10.02%	10.09%	13.69%
Short height (<145 cm)	1.43%	1.08%	0.48%	1.08%	1.10%	2.09%	0.33%	0.32%	0.67%
Low BMI (<18.5 kg/m^2)	0.82%	2.76%	1.02%	0.52%	2.03%	1.07%	1.38%	1.51%	2.09%
Chronic hypertension	3.74%	3.50%	7.12%	3.52%	3.57%	5.29%	6.87%	5.03%	6.42%
Twin pregnancy	1.83%	2.32%	2.89%	2.34%	2.37%	1.89%	2.79%	5.08%	2.61%
Fetal gender (male)	1.88%	1.77%	1.85%	1.78%	1.80%	1.94%	1.79%	2.01%	1.67%
Ambient air pollution	0.73%	1.93%	2.76%	2.73%	3.48%	2.05%	2.10%	3.85%	2.15%
Indoor air pollution	14.32%	13.32%	2.73%	11.91%	9.04%	2.12%	5.08%	3.53%	12.21%
Intimate partner violence	1.92%	4.05%	3.51%	4.07%	4.13%	4.57%	3.39%	2.48%	3.17%

Tobacco smoking	0.21%	0.14%	0.13%	0.15%	0.15%	1.03%	0.18%	0.04%	0.12%
Short cervical length (<25mm)	2.42%	2.28%	2.38%	2.29%	2.32%	2.50%	2.30%	2.59%	2.15%
Gestational diabetes	3.02%	3.12%	2.29%	3.14%	3.18%	2.46%	2.21%	1.64%	2.07%
Pre-eclampsia	1.07%	0.29%	1.00%	0.29%	0.30%	0.61%	0.97%	1.03%	0.90%
HIV	0.03%	0.03%	5.96%	0.59%	0.04%	0.37%	6.85%	0.03%	1.22%
HIV	1.89%	0.93%	0.00%	3.46%	0.86%	0.00%	0.01%	0.00%	2.32%
Malaria	0.30%	0.59%	2.34%	0.53%	0.63%	0.41%	1.88%	0.24%	1.70%
Syphilis	1.02%	0.55%	0.61%	0.55%	0.56%	1.29%	0.58%	0.39%	0.55%
Chlamydia	0.07%	0.05%	0.07%	0.05%	0.07%	0.11%	0.06%	0.19%	0.05%
Asymptomatic bacteriuria	0.33%	2.31%	0.96%	2.35%	2.49%	5.68%	0.57%	2.15%	0.32%
Periodontal Infection	8.66%	8.41%	11.39%	8.46%	8.56%	8.95%	10.99%	8.79%	10.27%
Total	66.61%	71.61%	66.88%	70.34%	69.00%	62.07%	69.82%	58.78%	75.85%

	Timor-Leste	Togo	Turkmenistan	Uganda	Uzbekistan	Venezuela	Yemen	Zambia	Zimbabwe
Short birth interval	2.05%	1.01%	2.21%	1.96%	2.61%	2.14%	2.27%	1.08%	0.73%
Young maternal age (< 18 years) & primiparity	0.57%	0.85%	2.17%	1.46%	0.43%	2.09%	1.00%	1.78%	1.62%
Maternal age 18-35 years & parity >3	4.02%	3.54%	1.60%	4.11%	4.07%	1.54%	4.67%	4.14%	3.65%
Older maternal age (>35 years) & parity >3	3.50%	3.12%	1.38%	2.64%	1.42%	1.33%	3.24%	2.94%	2.63%
Maternal Anemia	8.13%	14.42%	10.86%	9.60%	12.15%	9.72%	11.69%	10.18%	10.78%
Short height (<145 cm)	2.59%	0.18%	0.22%	0.33%	0.22%	2.13%	1.86%	0.45%	0.18%
Low BMI (<18.5 kg/m^2)	3.66%	1.83%	1.20%	2.00%	1.23%	0.52%	2.21%	1.94%	1.52%
Chronic hypertension	3.97%	6.06%	5.12%	6.29%	4.98%	5.38%	3.64%	6.32%	6.80%
Twin pregnancy	1.77%	2.46%	5.18%	2.56%	5.03%	1.92%	2.42%	2.57%	2.76%
Fetal gender (male)	1.82%	1.58%	2.05%	1.63%	1.99%	1.98%	1.84%	1.64%	1.77%
Ambient air pollution	1.89%	2.36%	3.05%	3.57%	3.36%	2.59%	3.57%	2.25%	2.19%
Indoor air pollution	12.91%	11.47%	0.17%	12.25%	1.43%	2.02%	5.46%	10.83%	8.43%
Intimate partner violence	2.55%	2.99%	2.52%	3.10%	2.45%	4.64%	4.21%	3.12%	3.35%
Tobacco smoking	0.67%	0.01%	1.48%	0.10%	1.44%	1.05%	0.15%	0.07%	0.05%
Short cervical length (<25mm)	2.34%	2.03%	2.63%	2.10%	2.56%	2.54%	2.37%	2.11%	2.27%
Gestational diabetes	2.93%	1.95%	1.67%	2.02%	1.63%	2.50%	3.25%	2.03%	2.19%

Pre-eclampsia	0.67%	0.85%	1.05%	0.88%	1.02%	0.62%	0.30%	0.89%	0.96%
HIV	0.03%	0.60%	0.03%	1.43%	0.03%	0.03%	0.03%	2.94%	3.25%
HIV	0.00%	7.30%	0.00%	4.36%	0.00%	0.11%	0.70%	4.47%	1.80%
Malaria	0.50%	1.65%	0.26%	1.68%	0.25%	0.58%	0.63%	1.64%	1.90%
Syphilis	0.29%	0.52%	0.40%	0.53%	0.39%	1.32%	0.57%	0.54%	0.58%
Chlamydia	0.04%	0.07%	0.19%	0.04%	0.20%	0.06%	0.07%	0.05%	0.06%
Asymptomatic bacteriuria	1.96%	3.07%	2.65%	1.85%	2.57%	4.29%	2.16%	1.97%	0.51%
Periodontal Infection	9.71%	9.69%	8.95%	10.05%	8.70%	9.11%	8.75%	10.11%	10.87%
Total	68.57%	79.59%	57.05%	76.55%	60.16%	60.21%	67.07%	76.06%	70.84%

References

1. Vogel JP, Lee AC, Souza JP. Maternal morbidity and preterm birth in 22 low- and middle-income countries: a secondary analysis of the WHO Global Survey dataset. BMC pregnancy and childbirth. 2014;14:56. Epub 2014/02/04. doi: 10.1186/1471-2393-14-56. PubMed PMID: 24484741; PubMed Central PMCID: PMCPMC3913333.
2. Embleton ND KJ, Ziegler EE. Low-Birthweight Baby: Born Too Soon or Too Small. Nestlé Nutr Inst Workshop Ser,. 2015;81:1-7. Epub 2015. doi: DOI: 10.1159/000365790.
3. Organization WH. Born Too Soon: The Global Action Report on Preterm Birth. 2012.
4. Goldenberg RL, Culhane JF, Iams JD, Romero R. Epidemiology and causes of preterm birth. Lancet (London, England). 2008;371(9606):75-84. Epub 2008/01/08. doi: 10.1016/S0140-6736(08)60074-4. PubMed PMID: 18177778.
5. Rubens CE, Sadovsky Y, Muglia L, Gravett MG, Lackritz E, Gravett C. Prevention of preterm birth: harnessing science to address the global epidemic. Sci Transl Med. 2014;6(262):262sr5. Epub 2014/11/14. doi: 10.1126/scitranslmed.3009871. PubMed PMID: 25391484.
6. Ota E, Ganchimeg T, Morisaki N, Vogel JP, Pileggi C, Ortiz-Panozo E, et al. Risk Factors and Adverse Perinatal Outcomes among Term and Preterm Infants Born Small-for-Gestational-Age: Secondary Analyses of the WHO Multi-Country Survey on Maternal and Newborn Health. PloS one. 2014;9(8). doi: ARTN e105155 10.1371/journal.pone.0105155. PubMed PMID: WOS:000340900600127.
7. Gravett MG, Rubens CE, Nunes TM, Group GR. Global report on preterm birth and stillbirth (2 of 7): discovery science. BMC pregnancy and childbirth. 2010;10 Suppl 1:S2. Epub 2010/03/27. doi: 10.1186/1471-2393-10-S1-S2. PubMed PMID: 20233383; PubMed Central PMCID: PMCPMC2841774.
8. Blencowe H, Cousens S, Chou D, Oestergaard M, Say L, Moller AB, et al. Born too soon: the global epidemiology of 15 million preterm births. Reprod Health. 2013;10 Suppl 1:S2. Epub 2014/03/15. doi: 10.1186/1742-4755-10-S1-S2. PubMed PMID: 24625129; PubMed Central PMCID: PMCPMC3828585.
9. Muglia LJ, Katz M. The enigma of spontaneous preterm birth. The New England journal of medicine. 2010;362(6):529-35. Epub 2010/02/12. doi: 10.1056/NEJMra0904308. PubMed PMID: 20147718.
10. Zhang J, Yu KF. What's the relative risk? A method of correcting the odds ratio in cohort studies of common outcomes. Jama. 1998;280(19):1690-1. Epub 1998/12/01. doi: 10.1001/jama.280.19.1690. PubMed PMID: 9832001.
11. Blencowe H, Cousens S, Oestergaard MZ, Chou D, Moller AB, Narwal R, et al. National, regional, and worldwide estimates of preterm birth rates in the year 2010 with time trends since 1990 for selected countries: a systematic analysis and implications. Lancet (London, England). 2012;379(9832):2162-72. Epub 2012/06/12. doi: 10.1016/S0140-6736(12)60820-4. PubMed PMID: 22682464.
12. Chawanpaiboon S, Vogel JP, Moller AB, Lumbiganon P, Petzold M, Hogan D, et al. Global, regional, and national estimates of levels of preterm birth in 2014: a systematic review and modelling analysis. Lancet Glob Health. 2019;7(1):e37-e46. Epub 2018/11/06. doi: 10.1016/S2214-109X(18)30451-0. PubMed PMID: 30389451; PubMed Central PMCID: PMCPMC6293055.
13. Kozuki N, Lee AC, Silveira MF, Victora CG, Adair L, Humphrey J, et al. The associations of birth intervals with small-for-gestational-age, preterm, and neonatal and infant mortality: a meta-analysis. BMC Public Health. 2013;13 Suppl 3:S3. Epub 2014/02/26. doi: 10.1186/1471-2458-13-S3-S3. PubMed PMID: 24564484; PubMed Central PMCID: PMCPMC3847557.
14. Kozuki N, Lee AC, Silveira MF, Sania A, Vogel JP, Adair L, et al. The associations of parity and maternal age with small-for-gestational-age, preterm, and neonatal and infant mortality: a meta-analysis. BMC Public Health. 2013;13 Suppl 3:S2. Epub 2014/02/26. doi: 10.1186/1471-2458-13-S3-S2. PubMed PMID: 24564800; PubMed Central PMCID: PMCPMC3847520.

15. Carducci B, Keats EC, Bhutta ZA. Zinc supplementation for improving pregnancy and infant outcome. The Cochrane database of systematic reviews. 2021;3:CD000230. Epub 2021/03/17. doi: 10.1002/14651858.CD000230.pub6. PubMed PMID: 33724446.
16. Rahman MM, Abe SK, Rahman MS, Kanda M, Narita S, Bilano V, et al. Maternal anemia and risk of adverse birth and health outcomes in low- and middle-income countries: systematic review and meta-analysis. Am J Clin Nutr. 2016;103(2):495-504. Epub 2016/01/08. doi: 10.3945/ajcn.115.107896. PubMed PMID: 26739036.
17. Hofmeyr GJ, Lawrie TA, Atallah AN, Torloni MR. Calcium supplementation during pregnancy for preventing hypertensive disorders and related problems. The Cochrane database of systematic reviews. 2018;10:CD001059. Epub 2018/10/03. doi: 10.1002/14651858.CD001059.pub5. PubMed PMID: 30277579; PubMed Central PMCID: PMCPMC6517256.
18. Kozuki N, Katz J, Lee AC, Vogel JP, Silveira MF, Sania A, et al. Short Maternal Stature Increases Risk of Small-for-Gestational-Age and Preterm Births in Low- and Middle-Income Countries: Individual Participant Data Meta-Analysis and Population Attributable Fraction. J Nutr. 2015;145(11):2542-50. Epub 2015/10/02. doi: 10.3945/jn.115.216374. PubMed PMID: 26423738; PubMed Central PMCID: PMCPMC6457093.
19. Han Z, Mulla S, Beyene J, Liao G, McDonald SD, Knowledge Synthesis G. Maternal underweight and the risk of preterm birth and low birth weight: a systematic review and meta-analyses. International journal of epidemiology. 2011;40(1):65-101. Epub 2010/11/26. doi: 10.1093/ije/dyq195. PubMed PMID: 21097954.
20. Morisaki N, Togoobaatar G, Vogel JP, Souza JP, Rowland Hogue CJ, Jayaratne K, et al. Risk factors for spontaneous and provider-initiated preterm delivery in high and low Human Development Index countries: a secondary analysis of the World Health Organization Multicountry Survey on Maternal and Newborn Health. BJOG : an international journal of obstetrics and gynaecology. 2014;121 Suppl 1:101-9. Epub 2014/03/20. doi: 10.1111/1471-0528.12631. PubMed PMID: 24641540.
21. Vogel JP, Torloni MR, Seuc A, Betran AP, Widmer M, Souza JP, et al. Maternal and perinatal outcomes of twin pregnancy in 23 low- and middle-income countries. PloS one. 2013;8(8):e70549. Epub 2013/08/13. doi: 10.1371/journal.pone.0070549. PubMed PMID: 23936446; PubMed Central PMCID: PMCPMC3731264.
22. Zeitlin J, Saurel-Cubizolles MJ, De Mouzon J, Rivera L, Ancel PY, Blondel B, et al. Fetal sex and preterm birth: are males at greater risk? Human reproduction (Oxford, England). 2002;17(10):2762-8. Epub 2002/09/28. doi: 10.1093/humrep/17.10.2762. PubMed PMID: 12351559.
23. Sun X LX, Zhao C, Ng RW, Lim CE, Zhang B, Liu T. The association between fine particulate matter exposure during pregnancy and preterm birth: a meta-analysis. BMC pregnancy and childbirth. 2015;15(1):300.
24. Amegah AK, Quansah R, Jaakkola JJ. Household air pollution from solid fuel use and risk of adverse pregnancy outcomes: a systematic review and meta-analysis of the empirical evidence. PloS one. 2014;9(12):e113920. Epub 2014/12/03. doi: 10.1371/journal.pone.0113920. PubMed PMID: 25463771; PubMed Central PMCID: PMCPMC4252082.
25. Donovan BM, Spracklen CN, Schweizer ML, Ryckman KK, Saftlas AF. Intimate partner violence during pregnancy and the risk for adverse infant outcomes: a systematic review and meta-analysis. BJOG : an international journal of obstetrics and gynaecology. 2016;123(8):1289-99. Epub 2016/03/10. doi: 10.1111/1471-0528.13928. PubMed PMID: 26956568.
26. Shah NR, Bracken MB. A systematic review and meta-analysis of prospective studies on the association between maternal cigarette smoking and preterm delivery. American journal of obstetrics and gynecology. 2000;182(2):465-72. doi: Doi 10.1016/S0002-9378(00)70240-7. PubMed PMID: WOS:000085582600033.
27. Iams JD, Goldenberg RL, Meis PJ, Mercer BM, Moawad A, Das A, et al. The length of the cervix and the risk of spontaneous premature delivery. National Institute of Child Health and Human Development Maternal Fetal Medicine Unit Network. The New England journal of medicine. 1996;334(9):567-72. Epub 1996/02/29. doi: 10.1056/NEJM199602293340904. PubMed PMID: 8569824.
28. Hedderson MM, Ferrara A, Sacks DA. Gestational diabetes mellitus and lesser degrees of pregnancy hyperglycemia: association with increased risk of spontaneous preterm birth. Obstetrics and gynecology. 2003;102(4):850-6. Epub 2003/10/11. PubMed PMID: 14551018.

29. Xiao PL, Zhou YB, Chen Y, Yang MX, Song XX, Shi Y, et al. Association between maternal HIV infection and low birth weight and prematurity: a meta-analysis of cohort studies. BMC pregnancy and childbirth. 2015;15:246. doi: ARTN 246
10.1186/s12884-015-0684-z. PubMed PMID: WOS:000362473900001.
30. Qin JB, Yang TB, Xiao SY, Tan HZ, Feng TJ, Fu HL. Reported Estimates of Adverse Pregnancy Outcomes among Women with and without Syphilis: A Systematic Review and Meta-Analysis. PloS one. 2014;9(7):e102203. doi: ARTN e102203
10.1371/journal.pone.0102203. PubMed PMID: WOS:000339992400050.
31. Olson-Chen C, Balaram K, Hackney DN. Chlamydia trachomatis and Adverse Pregnancy Outcomes: Meta-analysis of Patients With and Without Infection. Matern Child Hlth J. 2018;22(6):812-21. doi: 10.1007/s10995-018-2451-z. PubMed PMID: WOS:000430846900005.
32. Romero R, Oyarzun E, Mazor M, Sirtori M, Hobbins JC, Bracken M. Meta-Analysis of the Relationship between Asymptomatic Bacteriuria and Preterm Delivery Low Birth-Weight. Obstetrics and gynecology. 1989;73(4):576-82. PubMed PMID: WOS:A1989T803600008.
33. Corbella S, Taschieri S, Del Fabbro M, Francetti L, Weinstein R, Ferrazzi E. Adverse pregnancy outcomes and periodontitis: A systematic review and meta-analysis exploring potential association. Quintessence Int. 2016;47(3):193-204. Epub 2015/10/28. doi: 10.3290/j.qi.a34980. PubMed PMID: 26504910.
34. Leitich H, Kiss H. Asymptomatic bacterial vaginosis and intermediate flora as risk factors for adverse pregnancy outcome. Best Pract Res Cl Ob. 2007;21(3):375-90. doi: 10.1016/j.bpobgyn.2006.12.005. PubMed PMID: WOS:000247462000004.
35. Bianchi-Jassir F, Seale AC, Kohli-Lynch M, Lawn JE, Baker CJ, Bartlett L, et al. Preterm Birth Associated With Group B Streptococcus Maternal Colonization Worldwide: Systematic Review and Meta-analyses. Clinical infectious diseases : an official publication of the Infectious Diseases Society of America. 2017;65(suppl_2):S133-S42. Epub 2017/11/09. doi: 10.1093/cid/cix661. PubMed PMID: 29117329; PubMed Central PMCID: PMCPMC5850429.
36. Goldenberg RLI, J.D.; Mercer, B.M.; Meis, P.J.; Bottoms, S.J.; et al. The preterm prediction study: the value of new vs standard risk factors in predicting early and all spontaneous preterm births. American journal of public health. 1998;88(2):233-8.
37. Winer N, Resche-Rigon M, Morin C, Ville Y, Rozenberg P. Is induced abortion with misoprostol a risk factor for late abortion or preterm delivery in subsequent pregnancies? European journal of obstetrics, gynecology, and reproductive biology. 2009;145(1):53-6. Epub 2009/05/12. doi: 10.1016/j.ejogrb.2009.04.028. PubMed PMID: 19427095.
38. McCauley ME, van den Broek N, Dou L, Othman M. Vitamin A supplementation during pregnancy for maternal and newborn outcomes. The Cochrane database of systematic reviews. 2015(10):CD008666. Epub 2015/10/28. doi: 10.1002/14651858.CD008666.pub3. PubMed PMID: 26503498.
39. Palacios C, Kostiuk LK, Pena-Rosas JP. Vitamin D supplementation for women during pregnancy. The Cochrane database of systematic reviews. 2019;7:CD008873. Epub 2019/07/28. doi: 10.1002/14651858.CD008873.pub4. PubMed PMID: 31348529; PubMed Central PMCID: PMCPMC6659840.
40. McDonald SD, Han Z, Mulla S, Beyene J, Knowledge Synthesis G. Overweight and obesity in mothers and risk of preterm birth and low birth weight infants: systematic review and meta-analyses. BMJ (Clinical research ed). 2010;341:c3428. Epub 2010/07/22. doi: 10.1136/bmj.c3428. PubMed PMID: 20647282; PubMed Central PMCID: PMCPMC2907482.
41. Grote NK, Bridge JA, Gavin AR, Melville JL, Iyengar S, Katon WJ. A meta-analysis of depression during pregnancy and the risk of preterm birth, low birth weight, and intrauterine growth restriction. Arch Gen Psychiatry. 2010;67(10):1012-24. Epub 2010/10/06. doi: 10.1001/archgenpsychiatry.2010.111. PubMed PMID: 20921117; PubMed Central PMCID: PMCPMC3025772.
42. Snijder CA, Brand T, Jaddoe V, Hofman A, Mackenbach JP, Steegers EA, et al. Physically demanding work, fetal growth and the risk of adverse birth outcomes. The Generation R Study. Occup Environ Med. 2012;69(8):543-50. Epub 2012/06/30. doi: 10.1136/oemed-2011-100615. PubMed PMID: 22744766.

43. Lockwood CJ, Senyei AE, Dische MR, Casal D, Shah KD, Thung SN, et al. Fetal fibronectin in cervical and vaginal secretions as a predictor of preterm delivery. The New England journal of medicine. 1991;325(10):669-74. Epub 1991/09/05. doi: 10.1056/NEJM199109053251001. PubMed PMID: 1870640.
44. Hackney DN, Glantz JC. Vaginal bleeding in early pregnancy and preterm birth: systemic review and analysis of heterogeneity. The journal of maternal-fetal & neonatal medicine : the official journal of the European Association of Perinatal Medicine, the Federation of Asia and Oceania Perinatal Societies, the International Society of Perinatal Obstet. 2011;24(6):778-86. Epub 2010/12/15. doi: 10.3109/14767058.2010.530707. PubMed PMID: 21142755; PubMed Central PMCID: PMCPMC4547549.
45. Ananth CV, Berkowitz GS, Savitz DA, Lapinski RH. Placental abruption and adverse perinatal outcomes. Jama. 1999;282(17):1646-51. Epub 1999/11/30. doi: 10.1001/jama.282.17.1646. PubMed PMID: 10553791.
46. Kollmann M, Gaulhofer J, Lang U, Klaritsch P. Placenta praevia: incidence, risk factors and outcome. The journal of maternal-fetal & neonatal medicine : the official journal of the European Association of Perinatal Medicine, the Federation of Asia and Oceania Perinatal Societies, the International Society of Perinatal Obstet. 2016;29(9):1395-8. Epub 2015/06/05. doi: 10.3109/14767058.2015.1049152. PubMed PMID: 26043298.
47. Zhao R, Wang X, Zou L, Li G, Chen Y, Li C, et al. Adverse obstetric outcomes in pregnant women with uterine fibroids in China: A multicenter survey involving 112,403 deliveries. PloS one. 2017;12(11):e0187821. Epub 2017/11/15. doi: 10.1371/journal.pone.0187821. PubMed PMID: 29136018; PubMed Central PMCID: PMCPMC5685483.
48. Cotch MF, Pastorek JG, 2nd, Nugent RP, Hillier SL, Gibbs RS, Martin DH, et al. Trichomonas vaginalis associated with low birth weight and preterm delivery. The Vaginal Infections and Prematurity Study Group. Sexually transmitted diseases. 1997;24(6):353-60. Epub 1997/07/01. doi: 10.1097/00007435-199707000-00008. PubMed PMID: 9243743.
49. Salam RA, Haider BA, Humayun Q, Bhutta ZA. Effect of administration of antihelminthics for soil-transmitted helminths during pregnancy. The Cochrane database of systematic reviews. 2015(6):CD005547. Epub 2015/06/19. doi: 10.1002/14651858.CD005547.pub3. PubMed PMID: 26087057.
50. Conde-Agudelo A, Rosas-Bermudez A, Kafury-Goeta AC. Birth spacing and risk of adverse perinatal outcomes: a meta-analysis. Jama. 2006;295(15):1809-23. Epub 2006/04/20. doi: 10.1001/jama.295.15.1809. PubMed PMID: 16622143.
51. Saccone G, Perriera L, Berghella V. Prior uterine evacuation of pregnancy as independent risk factor for preterm birth: a systematic review and metaanalysis. American journal of obstetrics and gynecology. 2016;214(5):572-91. Epub 2016/01/09. doi: 10.1016/j.ajog.2015.12.044. PubMed PMID: 26743506.
52. Averbach SH, Seidman D, Steinauer J, Darney P. Re: Prior uterine evacuation of pregnancy as independent risk factor for preterm birth: a systematic review and metaanalysis. American journal of obstetrics and gynecology. 2017;216(1):87. Epub 2016/09/07. doi: 10.1016/j.ajog.2016.08.038. PubMed PMID: 27596618.
53. Oliver-Williams C, Fleming M, Monteath K, Wood AM, Smith GC. Changes in association between previous therapeutic abortion and preterm birth in Scotland, 1980 to 2008: a historical cohort study. PLoS Med. 2013;10(7):e1001481. Epub 2013/07/23. doi: 10.1371/journal.pmed.1001481. PubMed PMID: 23874161; PubMed Central PMCID: PMCPMC3706322.
54. Lemmers M, Verschoor MA, Hooker AB, Opmeer BC, Limpens J, Huirne JA, et al. Dilatation and curettage increases the risk of subsequent preterm birth: a systematic review and meta-analysis. Human reproduction (Oxford, England). 2016;31(1):34-45. Epub 2015/11/05. doi: 10.1093/humrep/dev274. PubMed PMID: 26534897.
55. Ota E, Mori R, Middleton P, Tobe-Gai R, Mahomed K, Miyazaki C, et al. Zinc supplementation for improving pregnancy and infant outcome. The Cochrane database of systematic reviews. 2015(2):CD000230. Epub 2015/05/01. doi: 10.1002/14651858.CD000230.pub5. PubMed PMID: 25927101.
56. Haider BA, Olofin I, Wang M, Spiegelman D, Ezzati M, Fawzi WW, et al. Anaemia, prenatal iron use, and risk of adverse pregnancy outcomes: systematic review and meta-analysis. BMJ (Clinical research ed).

2013;346:f3443. Epub 2013/06/26. doi: 10.1136/bmj.f3443. PubMed PMID: 23794316; PubMed Central PMCID: PMCPMC3689887.
57. Pena-Rosas JP, De-Regil LM, Garcia-Casal MN, Dowswell T. Daily oral iron supplementation during pregnancy. The Cochrane database of systematic reviews. 2015(7):CD004736. Epub 2015/07/23. doi: 10.1002/14651858.CD004736.pub5. PubMed PMID: 26198451.
58. Imdad A, Jabeen A, Bhutta ZA. Role of calcium supplementation during pregnancy in reducing risk of developing gestational hypertensive disorders: a meta-analysis of studies from developing countries. BMC Public Health. 2011;11 Suppl 3:S18. Epub 2011/04/29. doi: 10.1186/1471-2458-11-S3-S18. PubMed PMID: 21501435; PubMed Central PMCID: PMCPMC3231891.
59. De-Regil LM, Palacios C, Lombardo LK, Pena-Rosas JP. Vitamin D supplementation for women during pregnancy. Sao Paulo Med J. 2016;134(3):274-5. Epub 2016/06/30. doi: 10.1590/1516-3180.20161343T2. PubMed PMID: 27355803.
60. Imdad A, Bhutta ZA. Maternal nutrition and birth outcomes: effect of balanced protein-energy supplementation. Paediatric and perinatal epidemiology. 2012;26 Suppl 1:178-90. Epub 2012/07/07. doi: 10.1111/j.1365-3016.2012.01308.x. PubMed PMID: 22742610.
61. Girard AW, Olude O. Nutrition education and counselling provided during pregnancy: effects on maternal, neonatal and child health outcomes. Paediatric and perinatal epidemiology. 2012;26 Suppl 1:191-204. Epub 2012/07/07. doi: 10.1111/j.1365-3016.2012.01278.x. PubMed PMID: 22742611.
62. Abalos E, Duley L, Steyn DW, Gialdini C. Antihypertensive drug therapy for mild to moderate hypertension during pregnancy. The Cochrane database of systematic reviews. 2018;10:CD002252. Epub 2018/10/03. doi: 10.1002/14651858.CD002252.pub4. PubMed PMID: 30277556; PubMed Central PMCID: PMCPMC6517078.
63. Gelaye B, Rondon MB, Araya R, Williams MA. Epidemiology of maternal depression, risk factors, and child outcomes in low-income and middle-income countries. Lancet Psychiatry. 2016;3(10):973-82. Epub 2016/09/22. doi: 10.1016/S2215-0366(16)30284-X. PubMed PMID: 27650773; PubMed Central PMCID: PMCPMC5155709.
64. Sanchez SE, Puente GC, Atencio G, Qiu C, Yanez D, Gelaye B, et al. Risk of spontaneous preterm birth in relation to maternal depressive, anxiety, and stress symptoms. The Journal of reproductive medicine. 2013;58(1-2):25-33. Epub 2013/03/02. PubMed PMID: 23447915; PubMed Central PMCID: PMCPMC3662498.
65. Shah R, Mullany LC, Darmstadt GL, Mannan I, Rahman SM, Talukder RR, et al. Incidence and risk factors of preterm birth in a rural Bangladeshi cohort. BMC pediatrics. 2014;14:112. Epub 2014/04/25. doi: 10.1186/1471-2431-14-112. PubMed PMID: 24758701; PubMed Central PMCID: PMCPMC4021459.
66. Obiechina N, Okolie V, Eleje G, Okechukwu Z, Anemeje O. Twin versus singleton pregnancies: the incidence, pregnancy complications, and obstetric outcomes in a Nigerian tertiary hospital. Int J Womens Health. 2011;3:227-30. Epub 2011/08/17. doi: 10.2147/IJWH.S22059. PubMed PMID: 21845068; PubMed Central PMCID: PMCPMC3150208.
67. Chauhan SP, Scardo JA, Hayes E, Abuhamad AZ, Berghella V. Twins: prevalence, problems, and preterm births. American journal of obstetrics and gynecology. 2010;203(4):305-15. Epub 2010/08/24. doi: 10.1016/j.ajog.2010.04.031. PubMed PMID: 20728073.
68. Sung SJ, Lee SM, Kim S, Kim BJ, Park CW, Park JS, et al. The Risk of Spontaneous Preterm Birth according to Maternal Pre-pregnancy Body Mass Index in Twin Gestations. J Korean Med Sci. 2018;33(13):e103. Epub 2018/03/25. doi: 10.3346/jkms.2018.33.e103. PubMed PMID: 29573249; PubMed Central PMCID: PMCPMC5865054.
69. Challis J, Newnham J, Petraglia F, Yeganegi M, Bocking A. Fetal sex and preterm birth. Placenta. 2013;34(2):95-9. Epub 2012/12/25. doi: 10.1016/j.placenta.2012.11.007. PubMed PMID: 23261268.
70. Verburg PE, Tucker G, Scheil W, Erwich JJ, Dekker GA, Roberts CT. Sexual Dimorphism in Adverse Pregnancy Outcomes - A Retrospective Australian Population Study 1981-2011. PloS one. 2016;11(7):e0158807. Epub 2016/07/12. doi: 10.1371/journal.pone.0158807. PubMed PMID: 27398996; PubMed Central PMCID: PMCPMC4939964.

71. Peelen MJ, Kazemier BM, Ravelli AC, De Groot CJ, Van Der Post JA, Mol BW, et al. Impact of fetal gender on the risk of preterm birth, a national cohort study. Acta Obstet Gynecol Scand. 2016;95(9):1034-41. Epub 2016/05/25. doi: 10.1111/aogs.12929. PubMed PMID: 27216473.
72. Guo T, Wang Y, Zhang H, Zhang Y, Zhao J, Wang Q, et al. The association between ambient PM2.5 exposure and the risk of preterm birth in China: A retrospective cohort study. Sci Total Environ. 2018;633:1453-9. Epub 2018/05/16. doi: 10.1016/j.scitotenv.2018.03.328. PubMed PMID: 29758897.
73. Hill A, Pallitto C, McCleary-Sills J, Garcia-Moreno C. A systematic review and meta-analysis of intimate partner violence during pregnancy and selected birth outcomes. International journal of gynaecology and obstetrics: the official organ of the International Federation of Gynaecology and Obstetrics. 2016;133(3):269-76. Epub 2016/04/04. doi: 10.1016/j.ijgo.2015.10.023. PubMed PMID: 27039053.
74. Sigalla GN, Mushi D, Meyrowitsch DW, Manongi R, Rogathi JJ, Gammeltoft T, et al. Intimate partner violence during pregnancy and its association with preterm birth and low birth weight in Tanzania: A prospective cohort study. PloS one. 2017;12(2):e0172540. Epub 2017/02/25. doi: 10.1371/journal.pone.0172540. PubMed PMID: 28235031; PubMed Central PMCID: PMCPMC5325295.
75. Kiely M, El-Mohandes AA, El-Khorazaty MN, Blake SM, Gantz MG. An integrated intervention to reduce intimate partner violence in pregnancy: a randomized controlled trial. Obstetrics and gynecology. 2010;115(2 Pt 1):273-83. Epub 2010/01/23. doi: 10.1097/AOG.0b013e3181cbd482. PubMed PMID: 20093899; PubMed Central PMCID: PMCPMC2917915.
76. Saurel-Cubizolles MJ, Zeitlin J, Lelong N, Papiernik E, Di Renzo GC, Breart G, et al. Employment, working conditions, and preterm birth: results from the Europop case-control survey. J Epidemiol Community Health. 2004;58(5):395-401. Epub 2004/04/15. doi: 10.1136/jech.2003.008029. PubMed PMID: 15082738; PubMed Central PMCID: PMCPMC1732750.
77. Pompeii LA, Savitz DA, Evenson KR, Rogers B, McMahon M. Physical exertion at work and the risk of preterm delivery and small-for-gestational-age birth. Obstetrics and gynecology. 2005;106(6):1279-88. Epub 2005/12/02. doi: 10.1097/01.AOG.0000189080.76998.f8. PubMed PMID: 16319253.
78. Launer LJ, Villar J, Kestler E, de Onis M. The effect of maternal work on fetal growth and duration of pregnancy: a prospective study. Br J Obstet Gynaecol. 1990;97(1):62-70. Epub 1990/01/01. doi: 10.1111/j.1471-0528.1990.tb01718.x. PubMed PMID: 2306429.
79. Barnes DL, Adair LS, Popkin BM. Women's physical activity and pregnancy outcome: a longitudinal analysis from the Philippines. International journal of epidemiology. 1991;20(1):162-72. Epub 1991/03/01. doi: 10.1093/ije/20.1.162. PubMed PMID: 2066216.
80. Lumley J, Chamberlain C, Dowswell T, Oliver S, Oakley L, Watson L. Interventions for promoting smoking cessation during pregnancy. The Cochrane database of systematic reviews. 2009(3):CD001055. Epub 2009/07/10. doi: 10.1002/14651858.CD001055.pub3. PubMed PMID: 19588322; PubMed Central PMCID: PMCPMC4090746.
81. Chamberlain C, O'Mara-Eves A, Porter J, Coleman T, Perlen SM, Thomas J, et al. Psychosocial interventions for supporting women to stop smoking in pregnancy. The Cochrane database of systematic reviews. 2017;2:CD001055. Epub 2017/02/15. doi: 10.1002/14651858.CD001055.pub5. PubMed PMID: 28196405; PubMed Central PMCID: PMCPMC6472671.
82. Collaborators GBDRF, Forouzanfar MH, Alexander L, Anderson HR, Bachman VF, Biryukov S, et al. Global, regional, and national comparative risk assessment of 79 behavioural, environmental and occupational, and metabolic risks or clusters of risks in 188 countries, 1990-2013: a systematic analysis for the Global Burden of Disease Study 2013. Lancet (London, England). 2015;386(10010):2287-323. Epub 2015/09/15. doi: 10.1016/S0140-6736(15)00128-2. PubMed PMID: 26364544; PubMed Central PMCID: PMCPMC4685753.
83. Li X, Huang, Shuqiong, Jiao, Anqi, Yang, Xuhao, Yun, Junfeng, Liu, Yisi. Association between ambient fine particulate matter and preterm birth or term low birth weight: an updated systematic review and meta-analysis. Environmental Pollution. 2017;1(227):596-605.
84. Fleischer NL, Merialdi M, van Donkelaar A, Vadillo-Ortega F, Martin RV, Betran AP, et al. Outdoor air pollution, preterm birth, and low birth weight: analysis of the world health organization global survey on

maternal and perinatal health. Environ Health Perspect. 2014;122(4):425-30. Epub 2014/02/11. doi: 10.1289/ehp.1306837. PubMed PMID: 24508912; PubMed Central PMCID: PMCPMC3984219.
85. Akaraci S, Feng X, Suesse T, Jalaludin B, Astell-Burt T. A Systematic Review and Meta-Analysis of Associations between Green and Blue Spaces and Birth Outcomes. Int J Environ Res Public Health. 2020;17(8). Epub 2020/04/30. doi: 10.3390/ijerph17082949. PubMed PMID: 32344732; PubMed Central PMCID: PMCPMC7215926.
86. Alexander DA, Northcross A, Karrison T, Morhasson-Bello O, Wilson N, Atalabi OM, et al. Pregnancy outcomes and ethanol cook stove intervention: A randomized-controlled trial in Ibadan, Nigeria. Environ Int. 2018;111:152-63. Epub 2017/12/08. doi: 10.1016/j.envint.2017.11.021. PubMed PMID: 29216559.
87. Thakur M, Nuyts PAW, Boudewijns EA, Flores Kim J, Faber T, Babu GR, et al. Impact of improved cookstoves on women's and child health in low and middle income countries: a systematic review and meta-analysis. Thorax. 2018;73(11):1026-40. Epub 2018/06/22. doi: 10.1136/thoraxjnl-2017-210952. PubMed PMID: 29925674.
88. Abdel-Aleem H, Shaaban OM, Abdel-Aleem MA. Cervical pessary for preventing preterm birth. The Cochrane database of systematic reviews. 2013(5):CD007873. Epub 2013/06/04. doi: 10.1002/14651858.CD007873.pub3. PubMed PMID: 23728668; PubMed Central PMCID: PMCPMC6491132.
89. Berghella V, Rafael TJ, Szychowski JM, Rust OA, Owen J. Cerclage for short cervix on ultrasonography in women with singleton gestations and previous preterm birth: a meta-analysis. Obstetrics and gynecology. 2011;117(3):663-71. Epub 2011/03/31. PubMed PMID: 21446209.
90. Jarde A, Lutsiv O, Beyene J, McDonald SD. Vaginal progesterone, oral progesterone, 17-OHPC, cerclage, and pessary for preventing preterm birth in at-risk singleton pregnancies: an updated systematic review and network meta-analysis. BJOG : an international journal of obstetrics and gynaecology. 2019;126(5):556-67. Epub 2018/11/28. doi: 10.1111/1471-0528.15566. PubMed PMID: 30480871.
91. Honest H, Bachmann LM, Gupta JK, Kleijnen J, Khan KS. Accuracy of cervicovaginal fetal fibronectin test in predicting risk of spontaneous preterm birth: systematic review. BMJ (Clinical research ed). 2002;325(7359):301. Epub 2002/08/10. doi: 10.1136/bmj.325.7359.301. PubMed PMID: 12169504; PubMed Central PMCID: PMCPMC117763.
92. French JI, McGregor JA, Draper D, Parker R, McFee J. Gestational bleeding, bacterial vaginosis, and common reproductive tract infections: risk for preterm birth and benefit of treatment. Obstetrics and gynecology. 1999;93(5 Pt 1):715-24. Epub 2000/07/27. PubMed PMID: 10912974.
93. Strobino B, Pantel-Silverman J. Gestational vaginal bleeding and pregnancy outcome. Am J Epidemiol. 1989;129(4):806-15. Epub 1989/04/01. doi: 10.1093/oxfordjournals.aje.a115195. PubMed PMID: 2923126.
94. Yang J, Hartmann KE, Savitz DA, Herring AH, Dole N, Olshan AF, et al. Vaginal bleeding during pregnancy and preterm birth. Am J Epidemiol. 2004;160(2):118-25. Epub 2004/07/06. doi: 10.1093/aje/kwh180. PubMed PMID: 15234932.
95. Fox NS, Roman AS, Stern EM, Gerber RS, Saltzman DH, Rebarber A. Type of congenital uterine anomaly and adverse pregnancy outcomes. The journal of maternal-fetal & neonatal medicine : the official journal of the European Association of Perinatal Medicine, the Federation of Asia and Oceania Perinatal Societies, the International Society of Perinatal Obstet. 2014;27(9):949-53. Epub 2013/09/21. doi: 10.3109/14767058.2013.847082. PubMed PMID: 24050215.
96. Chan YY, Jayaprakasan K, Tan A, Thornton JG, Coomarasamy A, Raine-Fenning NJ. Reproductive outcomes in women with congenital uterine anomalies: a systematic review. Ultrasound Obstet Gynecol. 2011;38(4):371-82. Epub 2011/08/11. doi: 10.1002/uog.10056. PubMed PMID: 21830244.
97. Klatsky PC, Tran ND, Caughey AB, Fujimoto VY. Fibroids and reproductive outcomes: a systematic literature review from conception to delivery. American journal of obstetrics and gynecology. 2008;198(4):357-66. Epub 2008/04/09. doi: 10.1016/j.ajog.2007.12.039. PubMed PMID: 18395031.
98. Group HSCR, Metzger BE, Lowe LP, Dyer AR, Trimble ER, Chaovarindr U, et al. Hyperglycemia and adverse pregnancy outcomes. The New England journal of medicine. 2008;358(19):1991-2002. Epub 2008/05/09. doi: 10.1056/NEJMoa0707943. PubMed PMID: 18463375.

99. Martis R, Crowther CA, Shepherd E, Alsweiler J, Downie MR, Brown J. Treatments for women with gestational diabetes mellitus: an overview of Cochrane systematic reviews. The Cochrane database of systematic reviews. 2018;8:CD012327. Epub 2018/08/14. doi: 10.1002/14651858.CD012327.pub2. PubMed PMID: 30103263; PubMed Central PMCID: PMCPMC6513179.
100. Brown J, Grzeskowiak L, Williamson K, Downie MR, Crowther CA. Insulin for the treatment of women with gestational diabetes. The Cochrane database of systematic reviews. 2017;11:CD012037. Epub 2017/11/06. doi: 10.1002/14651858.CD012037.pub2. PubMed PMID: 29103210; PubMed Central PMCID: PMCPMC6486160.
101. Crowther CA, Hiller JE, Moss JR, McPhee AJ, Jeffries WS, Robinson JS, et al. Effect of treatment of gestational diabetes mellitus on pregnancy outcomes. The New England journal of medicine. 2005;352(24):2477-86. Epub 2005/06/14. doi: 10.1056/NEJMoa042973. PubMed PMID: 15951574.
102. Landon MB, Spong CY, Thom E, Carpenter MW, Ramin SM, Casey B, et al. A multicenter, randomized trial of treatment for mild gestational diabetes. The New England journal of medicine. 2009;361(14):1339-48. Epub 2009/10/03. doi: 10.1056/NEJMoa0902430. PubMed PMID: 19797280; PubMed Central PMCID: PMCPMC2804874.
103. Villar J, Abdel-Aleem H, Merialdi M, Mathai M, Ali MM, Zavaleta N, et al. World Health Organization randomized trial of calcium supplementation among low calcium intake pregnant women. American journal of obstetrics and gynecology. 2006;194(3):639-49. Epub 2006/03/09. doi: 10.1016/j.ajog.2006.01.068. PubMed PMID: 16522392.
104. Barros FC, Bhutta ZA, Batra M, Hansen TN, Victora CG, Rubens CE, et al. Global report on preterm birth and stillbirth (3 of 7): evidence for effectiveness of interventions. BMC pregnancy and childbirth. 2010;10 Suppl 1:S3. Epub 2010/03/27. doi: 10.1186/1471-2393-10-S1-S3. PubMed PMID: 20233384; PubMed Central PMCID: PMCPMC2841444.
105. Martin F, Taylor GP. The safety of highly active antiretroviral therapy for the HIV-positive pregnant mother and her baby: is 'the more the merrier'? The Journal of antimicrobial chemotherapy. 2009;64(5):895-900. Epub 2009/08/27. doi: 10.1093/jac/dkp303. PubMed PMID: 19706669.
106. Kourtis AP, Schmid CH, Jamieson DJ, Lau J. Use of antiretroviral therapy in pregnant HIV-infected women and the risk of premature delivery: a meta-analysis. AIDS. 2007;21(5):607-15. Epub 2007/02/23. doi: 10.1097/QAD.0b013e32802ef2f6. PubMed PMID: 17314523.
107. Steketee RW, Nahlen BL, Parise ME, Menendez C. The burden of malaria in pregnancy in malaria-endemic areas. The American journal of tropical medicine and hygiene. 2001;64(1-2 Suppl):28-35. Epub 2001/06/27. PubMed PMID: 11425175.
108. Nosten F, McGready R, Simpson JA, Thwai KL, Balkan S, Cho T, et al. Effects of Plasmodium vivax malaria in pregnancy. Lancet (London, England). 1999;354(9178):546-9. Epub 1999/09/02. PubMed PMID: 10470698.
109. Radeva-Petrova D, Kayentao K, ter Kuile FO, Sinclair D, Garner P. Drugs for preventing malaria in pregnant women in endemic areas: any drug regimen versus placebo or no treatment. Cochrane Database of Systematic Reviews. 2014(10). doi: ARTN CD000169
10.1002/14651858.CD000169.pub3. PubMed PMID: WOS:000347646000003.
110. Blencowe H, Cousens S, Kamb M, Berman S, Lawn JE. Lives Saved Tool supplement detection and treatment of syphilis in pregnancy to reduce syphilis related stillbirths and neonatal mortality. BMC Public Health. 2011;11 Suppl 3:S9. Epub 2011/04/29. doi: 10.1186/1471-2458-11-S3-S9. PubMed PMID: 21501460; PubMed Central PMCID: PMCPMC3231915.
111. Cluver C, Novikova, N., Eriksson, D. O., Bengtsson, K., & Lingman, G. K. Interventions for treating genital Chlamydia trachomatis infection in pregnancy. Cochrane Database of Systematic Reviews. 2017.
112. Sheiner E, Mazor-Drey E, Levy A. Asymptomatic bacteriuria during pregnancy. The journal of maternal-fetal & neonatal medicine : the official journal of the European Association of Perinatal Medicine, the Federation of Asia and Oceania Perinatal Societies, the International Society of Perinatal Obstet. 2009;22(5):423-7. Epub 2009/06/17. doi: 10.1080/14767050802360783. PubMed PMID: 19530000.

113. Schnarr J, Smaill F. Asymptomatic bacteriuria and symptomatic urinary tract infections in pregnancy. Eur J Clin Invest. 2008;38:50-7. doi: 10.1111/j.1365.2362.2008.02009.x. PubMed PMID: WOS:000262756500008.
114. Smaill FM, Vazquez JC. Antibiotics for asymptomatic bacteriuria in pregnancy. The Cochrane database of systematic reviews. 2019;2019(11). Epub 2019/11/26. doi: 10.1002/14651858.CD000490.pub4. PubMed PMID: 31765489; PubMed Central PMCID: PMCPMC6953361.
115. Lee AC, Mullany LC, Quaiyum M, Mitra DK, Labrique A, Christian P, et al. Effect of population-based antenatal screening and treatment of genitourinary tract infections on birth outcomes in Sylhet, Bangladesh (MIST): a cluster-randomised clinical trial. Lancet Glob Health. 2019;7(1):e148-e59. Epub 2018/12/18. doi: 10.1016/S2214-109X(18)30441-8. PubMed PMID: 30554751; PubMed Central PMCID: PMCPMC6293967.
116. Iheozor-Ejiofor Z, Middleton P, Esposito M, Glenny AM. Treating periodontal disease for preventing adverse birth outcomes in pregnant women. The Cochrane database of systematic reviews. 2017;6:CD005297. Epub 2017/06/13. doi: 10.1002/14651858.CD005297.pub3. PubMed PMID: 28605006; PubMed Central PMCID: PMCPMC6481493.
117. George A, Shamim S, Johnson M, Ajwani S, Bhole S, Blinkhorn A, et al. Periodontal treatment during pregnancy and birth outcomes: a meta-analysis of randomised trials. Int J Evid Based Healthc. 2011;9(2):122-47. Epub 2011/05/24. doi: 10.1111/j.1744-1609.2011.00210.x. PubMed PMID: 21599842.
118. Erchick DJ, Rai B, Agrawal NK, Khatry SK, Katz J, LeClerq SC, et al. Oral hygiene, prevalence of gingivitis, and associated risk factors among pregnant women in Sarlahi District, Nepal. BMC Oral Health. 2019;19(1):2. Epub 2019/01/07. doi: 10.1186/s12903-018-0681-5. PubMed PMID: 30611255; PubMed Central PMCID: PMCPMC6321675.
119. Brocklehurst P, Gordon A, Heatley E, Milan SJ. Antibiotics for treating bacterial vaginosis in pregnancy. The Cochrane database of systematic reviews. 2013(1):CD000262. Epub 2013/02/27. doi: 10.1002/14651858.CD000262.pub4. PubMed PMID: 23440777.
120. Baqui AH, Lee ACC, Koffi AK, Khanam R, Mitra DK, Dasgupta SK, et al. Prevalence of and risk factors for abnormal vaginal flora and its association with adverse pregnancy outcomes in a rural district in north-east Bangladesh. Acta Obstet Gynecol Scand. 2019;98(3):309-19. Epub 2018/10/23. doi: 10.1111/aogs.13492. PubMed PMID: 30346023; PubMed Central PMCID: PMCPMC6389396.
121. Thomsen AC, Mo/rup, L., & Hansen, K. B. Antibiotic elimination of group-B streptococci in urine in prevention of preterm labour. The Lancet. 1987;329(8533):591-3.
122. Klebanoff MA, Regan, J. A., Rao, A. V., Nugent, R. P., Blackwelder, W. C., Eschenbach, D. A., ... & Prematurity Study Group. Outcome of the Vaginal Infections and Prematurity Study: results of a clinical trial of erythromycin among pregnant women colonized with group B streptococci. American journal of obstetrics and gynecology. 1995;172(5):1540-5.
123. Anderson BL, Simhan, H. N., Simons, K. M., & Wiesenfeld, H. C. Untreated asymptomatic group B streptococcal bacteriuria early in pregnancy and chorioamnionitis at delivery. American journal of obstetrics and gynecology. 2007;196(6):524-e1.
124. WHO. WHO recommendations on antenatal care for a positive pregnancy experience. 2011.
125. ACOG. Group B Strep and Pregnancy: ACOG; 2017 [cited 2019 July 24]. Available from: https://www.acog.org/Patients/FAQs/Group-B-Strep-and-Pregnancy?IsMobileSet=false.
126. Bezold KY, Karjalainen MK, Hallman M, Teramo K, Muglia LJ. The genomics of preterm birth: from animal models to human studies. Genome Med. 2013;5(4):34. Epub 2013/05/16. doi: 10.1186/gm438. PubMed PMID: 23673148; PubMed Central PMCID: PMCPMC3707062.
127. Treloar SA, Macones GA, Mitchell LE, Martin NG. Genetic influences on premature parturition in an Australian twin sample. Twin Res. 2000;3(2):80-2. Epub 2000/08/06. PubMed PMID: 10918619.
128. Kistka ZA, DeFranco EA, Ligthart L, Willemsen G, Plunkett J, Muglia LJ, et al. Heritability of parturition timing: an extended twin design analysis. American journal of obstetrics and gynecology. 2008;199(1):43 e1-5. Epub 2008/02/26. doi: 10.1016/j.ajog.2007.12.014. PubMed PMID: 18295169.

129. Clausson B, Lichtenstein P, Cnattingius S. Genetic influence on birthweight and gestational length determined by studies in offspring of twins. BJOG : an international journal of obstetrics and gynaecology. 2000;107(3):375-81. Epub 2000/03/31. PubMed PMID: 10740335.
130. HIV/AIDS JUNPo. UNAIDS data 2018. 2018.
131. Organization WH. World Malaria Report. 2018.
132. Newman L, Rowley J, Vander Hoorn S, Wijesooriya NS, Unemo M, Low N, et al. Global Estimates of the Prevalence and Incidence of Four Curable Sexually Transmitted Infections in 2012 Based on Systematic Review and Global Reporting. PloS one. 2015;10(12):e0143304. Epub 2015/12/10. doi: 10.1371/journal.pone.0143304. PubMed PMID: 26646541; PubMed Central PMCID: PMCPMC4672879.
133. Kanyangarara M, Munos MK, Walker N. Quality of antenatal care service provision in health facilities across sub-Saharan Africa: Evidence from nationally representative health facility assessments. J Glob Health. 2017;7(2):021101. Epub 2017/11/23. doi: 10.7189/jogh.07.021101. PubMed PMID: 29163936; PubMed Central PMCID: PMCPMC5680531 form at www.icmje.org/coi_disclosure.pdf (available on request from the corresponding author) and declare no conflicts of interests.
134. Korenromp EL, Sudaryo MK, de Vlas SJ, Gray RH, Sewankambo NK, Serwadda D, et al. What proportion of episodes of gonorrhoea and chlamydia becomes symptomatic? International journal of STD & AIDS. 2002;13(2):91-101. Epub 2002/02/13. doi: 10.1258/0956462021924712. PubMed PMID: 11839163.
135. GBD results tool [Internet]. 2015. Available from: http://ghdx.healthdata.org/gbd-results-tool.
136. Mukherjee K, Golia, S., Vasudha, C. L., Bhattacharjee, D., & Chakroborti, G. . A study on asymptomatic bacteriuria in pregnancy: prevalence, etiology and comparison of screening methods. International Journal of Research in Medical Sciences. 2017;2(3):1085-91.
137. Torrone EA, Morrison CS, Chen PL, Kwok C, Francis SC, Hayes RJ, et al. Prevalence of sexually transmitted infections and bacterial vaginosis among women in sub-Saharan Africa: An individual participant data meta-analysis of 18 HIV prevention studies. Plos Medicine. 2018;15(2). doi: ARTN e1002511 10.1371/journal.pmed.1002511. PubMed PMID: WOS:000426459700010.
138. Peebles K, Velloza J, Balkus JE, McClelland RS, Barnabas RV. High Global Burden and Costs of Bacterial Vaginosis: A Systematic Review and Meta-Analysis. Sexually transmitted diseases. 2019;46(5):304-11. Epub 2019/01/10. doi: 10.1097/OLQ.0000000000000972. PubMed PMID: 30624309.
139. Kwatra G, Cunnington MC, Merrall E, Adrian PV, Ip M, Klugman KP, et al. Prevalence of maternal colonisation with group B streptococcus: a systematic review and meta-analysis. The Lancet Infectious diseases. 2016;16(9):1076-84. Epub 2016/05/30. doi: 10.1016/S1473-3099(16)30055-X. PubMed PMID: 27236858.
140. Wessells KR, Brown KH. Estimating the global prevalence of zinc deficiency: results based on zinc availability in national food supplies and the prevalence of stunting. PloS one. 2012;7(11):e50568. Epub 2012/12/05. doi: 10.1371/journal.pone.0050568. PubMed PMID: 23209782; PubMed Central PMCID: PMCPMC3510072.
141. Beal T, Massiot E, Arsenault JE, Smith MR, Hijmans RJ. Global trends in dietary micronutrient supplies and estimated prevalence of inadequate intakes. PloS one. 2017;12(4):e0175554. Epub 2017/04/12. doi: 10.1371/journal.pone.0175554. PubMed PMID: 28399168; PubMed Central PMCID: PMCPMC5388500.
142. Balk EM, Adam GP, Langberg VN, Earley A, Clark P, Ebeling PR, et al. Global dietary calcium intake among adults: a systematic review. Osteoporos Int. 2017;28(12):3315-24. Epub 2017/10/14. doi: 10.1007/s00198-017-4230-x. PubMed PMID: 29026938; PubMed Central PMCID: PMCPMC5684325.
143. Cormick G, Betran AP, Romero IB, Lombardo CF, Gulmezoglu AM, Ciapponi A, et al. Global inequities in dietary calcium intake during pregnancy: a systematic review and meta-analysis. BJOG : an international journal of obstetrics and gynaecology. 2019;126(4):444-56. Epub 2018/10/23. doi: 10.1111/1471-0528.15512. PubMed PMID: 30347499; PubMed Central PMCID: PMCPMC6518872.
144. Abalos E, Cuesta C, Grosso AL, Chou D, Say L. Global and regional estimates of preeclampsia and eclampsia: a systematic review. European journal of obstetrics, gynecology, and reproductive biology. 2013;170(1):1-7. Epub 2013/06/12. doi: 10.1016/j.ejogrb.2013.05.005. PubMed PMID: 23746796.

145. Stevens GA, Finucane MM, De-Regil LM, Paciorek CJ, Flaxman SR, Branca F, et al. Global, regional, and national trends in haemoglobin concentration and prevalence of total and severe anaemia in children and pregnant and non-pregnant women for 1995-2011: a systematic analysis of population-representative data. Lancet Glob Health. 2013;1(1):e16-25. Epub 2014/08/12. doi: 10.1016/S2214-109X(13)70001-9. PubMed PMID: 25103581; PubMed Central PMCID: PMCPMC4547326.
146. Mills KT, Bundy JD, Kelly TN, Reed JE, Kearney PM, Reynolds K, et al. Global Disparities of Hypertension Prevalence and Control: A Systematic Analysis of Population-Based Studies From 90 Countries. Circulation. 2016;134(6):441-50. Epub 2016/08/10. doi: 10.1161/CIRCULATIONAHA.115.018912. PubMed PMID: 27502908; PubMed Central PMCID: PMCPMC4979614.
147. United Nations DoEaSA, Population Division World Population Prospects: The 2018 Revision. 2018.
148. Zhu Y, Zhang C. Prevalence of Gestational Diabetes and Risk of Progression to Type 2 Diabetes: a Global Perspective. Curr Diab Rep. 2016;16(1):7. Epub 2016/01/09. doi: 10.1007/s11892-015-0699-x. PubMed PMID: 26742932.
149. Smits J, Monden C. Twinning across the Developing World. PloS one. 2011;6(9):e25239. Epub 2011/10/08. doi: 10.1371/journal.pone.0025239. PubMed PMID: 21980404; PubMed Central PMCID: PMCPMC3182188.
150. Martin JA, Hamilton BE, Osterman MJ, Curtin SC, Matthews TJ. Births: final data for 2012. Natl Vital Stat Rep. 2013;62(9):1-68. Epub 2013/12/01. PubMed PMID: 25671704.
151. ICF. The DHS Program STATcompiler. 2012.
152. Lange S, Probst C, Rehm J, Popova S. National, regional, and global prevalence of smoking during pregnancy in the general population: a systematic review and meta-analysis. Lancet Glob Health. 2018;6(7):e769-e76. Epub 2018/06/04. doi: 10.1016/S2214-109X(18)30223-7. PubMed PMID: 29859815.
153. Thakur M, Mahajan K. Cervical Incompetence. StatPearls. Treasure Island (FL)2019.
154. Shennan A, Jones B. The cervix and prematurity: aetiology, prediction and prevention. Seminars in fetal & neonatal medicine. 2004;9(6):471-9. Epub 2005/02/05. doi: 10.1016/j.siny.2004.09.001. PubMed PMID: 15691785.
155. Z Muhammad SI. Cervical incompetence and pregnancy outcome in Jos University Teaching Hospital: A four -year review. Borno Medical Journal Online. 2006;3(1).
156. AS Adeniran AA, EU Okpara, AA Fawole, KT Adesina Pregnancy outcome in cervical incompetence: Comparison before and after intervention. Tropical Journal of Obstetrics and Gynaecology. 2014;31:23-9.
157. Malipati Maerdan CS, Xiaoxiao Zhang, Lixin Fan. The prevalence of short cervix between 20 and 24 weeks of gestation and vaginal progesterone for prolonging of gestation. The Journal of Maternal-Fetal & Neonatal Medicine. 2017;30:1646-9.
158. Collaborators GBDRF. Global, regional, and national comparative risk assessment of 79 behavioural, environmental and occupational, and metabolic risks or clusters of risks, 1990-2015: a systematic analysis for the Global Burden of Disease Study 2015. Lancet (London, England). 2016;388(10053):1659-724. Epub 2016/10/14. doi: 10.1016/S0140-6736(16)31679-8. PubMed PMID: 27733284; PubMed Central PMCID: PMCPMC5388856.
159. Ritchie H, Roser, M. . Indoor air pollution Published online at OurWorldInData.org2013 [cited 2021]. Available from: https://ourworldindata.org/indoor-air-pollution#citation.
160. Devries KM, Kishor S, Johnson H, Stockl H, Bacchus LJ, Garcia-Moreno C, et al. Intimate partner violence during pregnancy: analysis of prevalence data from 19 countries. Reprod Health Matters. 2010;18(36):158-70. Epub 2010/11/30. doi: 10.1016/S0968-8080(10)36533-5. PubMed PMID: 21111360.
161. Garcia-Moreno CJ, H; Ellsberg, M; Heise, L; Watts C. WHO Multi-country Study on Womens Health and Domestic Violence against Women. Initial results on prevalence, health outcomes, and women's responses. 2005.
162. Organization WH. Global and regional estimates of violence against women: prevalence and health effects of intimate partner violence and nonpartner sexual violence. 2013.
